# The Mediator Subunit MDT-15 Confers Metabolic Adaptation to Ingested Material

**DOI:** 10.1371/journal.pgen.1000021

**Published:** 2008-02-29

**Authors:** Stefan Taubert, Malene Hansen, Marc R. Van Gilst, Samantha B. Cooper, Keith R. Yamamoto

**Affiliations:** 1 Department of Cellular and Molecular Pharmacology, University of California San Francisco, San Francisco, California, United States of America; 2 Department of Biochemistry and Biophysics, University of California San Francisco, San Francisco, California, United States of America; 3 Fred Hutchinson Cancer Research Center, Basic Sciences Division, Seattle, Washington, United States of America; 4 Graduate Program of Biological and Medical Informatics, University of California San Francisco, San Francisco, California, United States of America; Huntsman Cancer Institute, United States of America

## Abstract

In eukaryotes, RNA polymerase II (Pol_II_) dependent gene expression requires accessory factors termed transcriptional coregulators. One coregulator that universally contributes to Pol_II_-dependent transcription is the Mediator, a multisubunit complex that is targeted by many transcriptional regulatory factors. For example, the *Caenorhabditis elegans* Mediator subunit MDT-15 confers the regulatory actions of the sterol response element binding protein SBP-1 and the nuclear hormone receptor NHR-49 on fatty acid metabolism. Here, we demonstrate that MDT-15 displays a broader spectrum of activities, and that it integrates metabolic responses to materials ingested by *C. elegans*. Depletion of MDT-15 protein or mutation of the *mdt-15* gene abrogated induction of specific detoxification genes in response to certain xenobiotics or heavy metals, rendering these animals hypersensitive to toxin exposure. Intriguingly, MDT-15 appeared to selectively affect stress responses related to ingestion, as MDT-15 functional defects did not abrogate other stress responses, *e.g.,* thermotolerance. Together with our previous finding that MDT-15:NHR-49 regulatory complexes coordinate a sector of the fasting response, we propose a model whereby MDT-15 integrates several transcriptional regulatory pathways to monitor both the availability and quality of ingested materials, including nutrients and xenobiotic compounds.

## Author Summary

All organisms adapt their physiology to external input, such as altered food availability or toxic challenges. Many of these responses are driven by changes in gene transcription. In general, sequence specific DNA-binding regulatory factors are considered the specificity determinants of the transcriptional output. Here, we show that, in the roundworm Caenorhabditis elegans, one subunit of a >20 subunit, evolutionarily conserved, non-DNA binding co-factor termed Mediator, specifies a portion of the metabolic responses to a mixture of ingested material. This protein, MDT-15, is required for appropriate expression of genes that protect worms from the effects of toxic compounds and heavy metals. Our previous findings showed that the same protein also cooperates with other regulators to coordinate lipid metabolism. We suggest that MDT-15 may “route” transcriptional responses appropriate to the ingested material. This physiological scope appears broader and more sophisticated than that of any individual regulatory factor, thus coordinating systemic metabolic adaptation with ingestion. Given the evolutionary conservation of MDT-15 and the Mediator, a similar regulatory pathway may ensure health and longevity in mammals.

## Introduction

Eukaryotic gene transcription requires the concerted interplay of many factors. DNA-binding factors nucleate specific regulatory complexes on individual genes, culminating in assembly of functional RNA polymerase II (Pol_II_). These complexes also contain transcriptional cofactors that serve various functions, such as chromatin remodeling and chromatin modification. Within this complex machinery, the sequence specific regulatory factors are generally thought to be the primary determinants that specify transcriptional output in response to a certain signal.

The Mediator is a conserved multi-protein coregulatory complex that, at the minimum, serves a critical linking function between regulatory factors and the transcription initiation machinery [1]–[3]. Some Mediator subunits, such as yeast Med17 are required for essentially all Pol_II_-driven transcription [4]. Similarly, in the nematode *Caenorhabditis elegans* the subunit MDT-14/RGR-1 is broadly required for early embryonic transcription [5]. However, in both organisms, some Mediator subunits are required only for expression of a restricted subset of all Pol_II_-transcribed genes [4]–[7]. Indeed, many Mediator components influence specific physiological and/or developmental processes. For instance, mammalian MED1/TRAP220 is utilized by nuclear hormone receptors (NHRs) to implement programs such as adipogenesis (through peroxisome proliferators activated receptor γ (PPARγ) [8]) and systemic detoxification (through the pregnane-X-receptor (PXR) and the constitutive androstane receptor (CAR) [9],[10]). Likewise, the *C. elegans* Mediator subunits MDT-12/DPY-22, MDT-13/LET-19, and MDT-1.1/SOP-3 participate in vulva or male tail development [7], [11]–[13]. These studies raise the question of how individual components within the same regulatory complex can exert such separable effects. Also, it is unclear whether the specific functions of the coregulators are broader or narrower than those of the sequence specific regulatory factors with which they interact. Moreover, although certain Mediator subunits differentially affect related functions, the relationship between Mediator subunit utilization and a transcriptional mechanism or a physiological process is not known. Deciphering the mechanistic contributions of individual Mediator components to transcription is relevant in view of Mediator's conservation and its capacity to interact with numerous regulatory factors, thus influencing many biological processes [14].

In a previous study we found that the *C. elegans* Mediator subunit MDT-15 integrates expression of certain metabolic genes in NHR-49-dependent and -independent ways [15]. Others found that MDT-15 conveys regulation of fatty acid (FA) desaturases by the basic helix-loop-helix zipper protein SBP-1, the *C. elegans* ortholog of the mammalian sterol regulatory element binding proteins (SREBPs) that regulate FA and cholesterol metabolism [16]. Therefore, NHR-49 and SBP-1 appear to collaborate with MDT-15/MED15 to affect overlapping yet distinct sectors of metabolic genes. Hence, MDT-15 exhibits a broader spectrum of physiologic regulation than either individual regulatory factor, and could be viewed as an important node in a regulatory network that maintains metabolic homeostasis. Thus, analysis of individual Mediator components might reveal both upstream regulatory inputs and downstream regulatory mechanisms within a critical gene network.

To connect the function of MDT-15 in transcription to its precise physiologic role, we sought to more broadly define MDT-15's sphere of influence. As an initial step, we set out to globally discover new MDT-15-dependent genes in an unbiased fashion, and thus to identify previously unrecognized biological processes that lie downstream of MDT-15.

## Results

### Identification of Novel MDT-15 Targets by Gene Expression Profiling

We previously showed that the *C. elegans* Mediator subunit MDT-15 impacts expression of select genes involved in fatty acid (FA) metabolism [15]. To identify MDT-15's physiological targets in an unbiased manner, we used expression microarrays to compare the transcriptional profiles in wild-type (N2) worms that were fed for 40 hr (*i.e.* allowed to synchronously develop to larval stage L4; see Materials and Methods) with bacteria harboring vectors for either control or *mdt-15* RNA-interference (RNAi; reviewed in [17]). To identify statistically significant changes in gene expression following MDT-15 depletion we analyzed the raw data using “linear models for microarray data” (limma [18],[19]; see Materials and Methods). In total we found 187 genes that were downregulated, and 120 genes that were upregulated (*P*-value cutoff 0.05; Table S1). An alternative approach, “significance analysis of microarrays” [20], revealed similar numbers and similar sets of affected genes (data not shown). These numbers support the hypothesis that MDT-15 is essential for expression of only a subset of genes in C. *elegans* [15]. Given that MDT-15 and its mammalian orthologs have thus far been characterized as coactivators [15],[16],[21], we focused this study on genes with reduced mRNA levels in *mdt-15(RNAi)* animals.

The genes identified as downregulated in *mdt-15(RNAi)* animals included 12 previously identified MDT-15 target genes involved in lipid metabolism [15], and *mdt-15* itself, thus validating our experimental approach (Figure S1, and Table S1). In order to verify previously unrecognized candidate MDT-15-targets, we performed quantitative real-time PCR (qPCR) analysis on total RNA from worms grown on either control RNAi or on *mdt-15* RNAi bacteria (see Materials and Methods). In total we tested 85 of the 187 downregulated candidate MDT-15 targets by this method, primarily focusing on genes with orthologs in mammals, and genes predicted to participate in metabolism and detoxification (see below). We found that 63 of 85 genes (74%) were downregulated more than two-fold (Figure S1 and Table S2; note that, in this validation assay, the two-fold cut-off yields false negatives, as our limma analysis assessed significant change without a specific two-fold minimal change threshold). Thus, our microarray-based strategy was an effective tool to identify novel MDT-15 targets.

To corroborate the data obtained with RNAi depletion of endogenous MDT-15 we obtained a strain carrying a mutation in the *mdt-15* gene, *mdt-15(tm2182)* (strain XA7702; see Materials and Methods and Figure S2A). This strain recapitulates the phenotypes evoked by *mdt-15* RNAi, including shortened life span (Figure S2B), reduced fecundity (Figure S2C), clear appearance, and altered fat storage (determined by Nile Red staining [22]; data not shown). We determined by qPCR the relative mRNA levels of 97 MDT-15 targets (85 new candidate genes from the microarrays and the 12 previously known MDT-15 targets [15]) in wild-type N2 and mutant *mdt-15(tm2182)* worms harvested at the L4 stage. We found that 50 of 97 genes (52%) exhibited more than two-fold deregulation in *mdt-15(tm2182)* mutants (Figure S1 and Table S3); 47 of these 50 genes were also deregulated in *mdt-15(RNAi)* worms. In contrast, 28 genes deregulated more than two-fold in *mdt-15(RNAi)* worms were not affected to this extent in *mdt-15(tm2182)* mutants, although most genes were still downregulated to some extent (and only two were upregulated in *mdt-15(tm2182)* worms). Thus, *mdt-15(tm2182)* mutants recapitulate many but not all of the gene expression defects exhibited by *mdt-15(RNAi)* worms. Overall, the gene expression changes in *mdt-15(tm2182)* worms appear less severe than those presented by *mdt-15(RNAi)* worms, suggesting that *mdt-15(tm2182)* may represent a hypomorphic allele. Alternatively, as the mutation is predicted to produce a truncated MDT-15, and as *mdt-15* mRNA levels appear normal in *mdt-15(tm2182)* worms (Table S3), it is possible that truncated MDT-15 dominantly interferes with Mediator complex function. In any case, the data support our results obtained by RNAi depletion of endogenous MDT-15 and also establish the *mdt-15(tm2182)* mutant as a valuable tool to study MDT-15 function.

### Many New MDT-15 Targets Are Involved in Lipid Metabolism and Xenobiotic Detoxification

To identify pathways and molecular functions common to the 187 genes observed by microarray analysis to be downregulated after MDT-15 depletion, we employed the DAVID gene ontology (GO) annotation tool [23]. As expected, we found a significant overrepresentation of GO-terms for functions related to lipid metabolism (Table 1). In addition, we used DAVID to query the same gene set for protein domains. We found that domains involved in lipid metabolism were overrepresented (*e.g.* FA-Δ9-desaturases and FA-oxidases). These results confirm the importance of MDT-15 in regulation of lipid metabolic genes.

**Table 1:** Occurrence of gene families in microarray results, based on GO-terms.

Term	Count	%	*P*-Value
Organic acid metabolism	15	8.2%	4.2×10^−9^
Fatty acid metabolism	9	4.9%	1.2×10^−8^
Cellular lipid metabolism	10	5.5%	8.0×10^−7^
Lipid metabolism	12	6.6%	7.7×10^−6^
Generation of precursor metabolites and energy	16	8.8%	8.8×10^−6^
Electron transport	14	7.7%	9.7×10^−6^
Fatty acid beta-oxidation	4	2.2%	7.9×10^−5^
Fatty acid biosynthesis	4	2.2%	8.5×10^−4^
Cellular metabolism	47	25.8%	1.6×10^−3^
Cell wall catabolism	3	1.6%	5.2×10^−3^
Cellular carbohydrate metabolism	6	3.3%	7.9×10^−3^
Transport	26	14.3%	1.1×10^−2^
Carbohydrate metabolism	7	3.8%	1.4×10^−2^
Catabolism	7	3.8%	1.5×10^−2^
Lipid biosynthesis	4	2.2%	2.1×10^−2^
Amine metabolism	6	3.3%	2.2×10^−2^
Establishment of localization	26	14.3%	2.2×10^−2^
Nitrogen compound metabolism	6	3.3%	2.4×10^−2^
Osmoregulation	6	3.3%	2.8×10^−2^
Peptidoglycan catabolism	2	1.1%	4.0×10^−2^
Aspartate family amino acid metabolism	2	1.1%	4.9×10^−2^
Aldehyde metabolism	2	1.1%	4.9×10^−2^

Occurrence of gene ontology terms in genes that, by microarray analysis, were found to be downregulated in *mdt-15(RNAi)* worms (*vs. control(RNAi)* worms), ranked by lowest *P*-value. 118 genes (of 187 total) were not classified by this analysis. “Count” indicates the number of genes amongst MDT-15 targets that fit the respective term. “%” indicates the percentage of these amongst all MDT-15 targets. “*P*-Value” indicates the statistical significance of the overrepresentation of an individual category; we used a *P*-value of 0.05 as cutoff.

Unexpectedly, we also discovered other enriched domains subject to MDT-15 dependence, specifically UDP-glucuronosyl/UDP-glucosyltransferases (UGTs; 15 genes of the 187 MDT-15 candidate MDT-15 dependent genes), glutathione S-transferases (GSTs; five genes), short-chain dehydrogenase/reductase SDRs (DHSs; five genes), and FAD-domains (two genes; see Table 2). Although proteins containing any of these domains may metabolize lipids, they are typically associated with systemic metabolism and clearance of endo- and xenobiotic compounds (see below). In addition to these predicted detoxification enzymes, the gene set exhibited overrepresentation of proteins containing the DUF227 domain (six genes). *Drosophila melanogaster* proteins harboring DUF227 domains are implicated in insecticide resistance [24], and some *D. melanogaster* DUF227 genes are induced by Phenobarbital [25]. In total, MDT-15 dependent genes are enriched for five protein families associated with detoxification.

**Table 2:** Occurrence of protein domains in microarray results, based on InterPro domains.

Term	Count	%	*P*-Value
UDP-glucuronosyl/UDP-glucosyltransferase	15	8.2%	3.5×10^−13^
Acyl-CoA dehydrogenase, central region	7	3.8%	2.7×10^−7^
Protein of unknown function DUF227	6	3.3%	1.4×10^−5^
Protein of unknown function DUF1679, *Caenorhabditis* sp.	5	2.7%	6.2×10^−5^
Acyl-CoA oxidase, C-terminal	4	2.2%	8.6×10^−5^
Fatty acid desaturase	4	2.2%	1.3×10^−4^
Glucose/ribitol dehydrogenase	6	3.3%	1.1×10^−3^
Glutathione S-transferase, N-terminal	5	2.7%	2.8×10^−3^
Short-chain dehydrogenase/reductase SDR	5	2.7%	1.3×10^−2^
CUB-like region	4	2.2%	2.2×10^−2^
Thioredoxin-like fold	6	3.3%	2.5×10^−2^
FAD linked oxidase, C-terminal	2	1.1%	3.5×10^−2^

Protein domains (based on InterPro annotation) enriched in genes that, by microarray analysis, were found to be downregulated in *mdt-15(RNAi)* worms (*vs. control(RNAi)* worms), ranked by lowest *P*-value. 122 genes (of 187 total genes) were not classified by this analysis. “Count” indicates the number of genes amongst MDT-15 targets that fit the respective term. “%” indicates the percentage of these amongst MDT-15 targets. “*P*-Value” indicates the statistical significance of the overrepresentation of an individual category amongst all MDT-15 targets; we used a *P*-value of 0.05 as cutoff.

In addition to the statistically overrepresented protein families, the MDT-15 dependent genes included three cytochrome P450s (CYP450s), three alcohol and aldehyde dehydrogenases, two ABC-transporters, and two acyl-CoA-synthetases (ACSs). These are all members of protein families associated with detoxification. Interestingly, the ACS proteins represent a class of lipid metabolizing enzymes, some members of which can metabolize xenobiotics [26]. Of note, we previously found that two other *acs* genes, *acs-2* and *acs-11*, respond to short-term fasting in an MDT-15 dependent fashion [15],[27]. Finally, the list of MDT-15-dependent genes included four genes (a metallothionein, *mtl-2*; a cadmium-responsive gene, *cdr-6*; a predicted zinc-transporter, T18D3.3; and a predicted selenium-binding protein, Y37A1B.5) likely involved in heavy metal detoxification. In summary, 47 of 187 genes (25%) that our microarray analysis identified as significantly downregulated in *mdt-15(RNAi)* worms may be involved in metabolism or elimination of toxic substances (Table S1). This number is similar to the number of MDT-15 dependent genes related to energy metabolism (38 genes, *i.e.* 20%), suggesting that both processes compose important sectors within MDT-15's sphere of influence (Table S1).

DAVID analysis of the 120 genes upregulated in *mdt-15(RNAi)* worms revealed fewer enriched functions and domains (Table S4). Among these, upregulation of genes with functions/domains in energy metabolism and in protein folding/degradation may occur as compensation for (metabolic) challenges imposed by MDT-15 depletion. In addition, we noted the enrichment of CUB and saposin domains, both of which have been associated with pathogen defense [28],[29]; it may be interesting in future experiments to examine these genes in the context of MDT-15's biological role.

### Tissue-Restricted Expression of MDT-15 Targets

MDT-15 is highly expressed in the intestine [15],[30], which is an organ important for nutrient ingestion and digestion in *C. elegans*, and is also thought to be the principle organ for detoxification [31]. Thus, one might expect that MDT-15-targets preferentially exhibit intestinal expression. To address this notion we compared the set of genes identified by our microarrays as downregulated in *mdt-15(RNAi)* worms to published sets of genes exhibiting tissue specific expression in *C. elegans* [32],[33]. We found that many of the genes downregulated in *mdt-15(RNAi)* worms are intestine-selective (22%); in contrast, no MDT-15 target genes were enriched in muscle or germ line, and only 2% were pharynx-specific (Table S5). Comparison of our gene set to a *C. elegans* gene expression map integrating 553 individual microarray experiments [34] also revealed enrichment for intestinal genes, and depletion of germline, neuronal, and muscle specific genes (data not shown). Finally, the 120 genes induced in *mdt-15(RNAi)* worms also exhibited enrichment of intestine-selective genes (data not shown). This selective overrepresentation among MDT-15 targets of intestinally expressed genes fits well with the roles of MDT-15 in lipid metabolism [15],[16], and detoxification (see below).

### MDT-15 Is Required for Induction of Detoxification Genes in Response to Toxins

MDT-15 is required for both basal and fasting-induced transcription of certain energy metabolism genes [15]. We reasoned that, because MDT-15 is essential for basal expression of several predicted detoxification genes (Table 3 and Tables S1,S2,S3), it might similarly be critical for toxin-activated gene expression. Consistent with the notion that *C. elegans ugt*, *gst*, *dhs,* and *cyp* genes (encoding CYP450s) are important for detoxification, several of these genes are transcriptionally induced when wild type worms are exposed to toxic chemicals (reviewed in [35]). Interestingly, this includes *ugt-1* and *cyp-35C1* [36] whose basal expression we found to be MDT-15 dependent (Table 3 and Tables S1,S2,S3).

**Table 3:** MDT-15 depletion compromises induction of select detoxification genes by the toxins fluoranthene (FLA) and β-naphthoflavone (NF).

	RNAi	Overall fold change *vs.* control RNAi						Fold change caused by toxin *vs.* solvent (within same RNAi condition)			
	Toxin	*control*	*control*	*control*	*mdt-15*	*mdt-15*	*mdt-15*	*control*	*mdt-15*	*control*	*mdt-15*
Function	Gene	DMSO	FLA	NF	DMSO	FLA	NF	FLA *vs.* DMSO	FLA *vs.* DMSO	NF *vs.* DMSO	NF *vs.* DMSO
DUF227	T16G1.6	1±0	3.12±0.57	1.77±0.57	0.09±0.05	0.33±0.26	0.29±0.19	3.1	3.6	1.8	3.1
Hydrolase	F37H8.3	1±0	2.41±0.36	1.23±0.38	0.16±0.04	0.3±0.06	0.81±0.57	2.4	1.8	1.2	5.0
**ADH**	***alh-5***	1±0	4.27±0.21	3.66±1.32	1.23±0.56	1.25±0.44	2.66±1.82	4.3	1.0	3.7	2.2
**UGT**	***ugt-1***	1±0	4.67±0.64	2.55±0.43	0.33±0.14	0.65±0.38	0.92±0.75	4.7	2.0	2.5	2.8
UGT	*ugt-58*	1±0	2.2±0.3	1.11±0.21	0.48±0.04	0.88±0.04	0.6±0.13	2.2	1.8	1.1	1.2
UGT	*ugt-25*	1±0	7.1±0.32	2.98±0.49	0.27±0.06	1.9±0.23	1.17±0.49	7.1	6.9	3.0	4.3
UGT	*ugt-13*	1±0	4.5±0.9	2.73±0.87	0.22±0.08	1.27±0.57	0.68±0.24	4.5	5.6	2.7	3.0
**GST**	***gst-5***	1±0	10.55±1.31	2.29±0.66	0.73±0.12	1.75±0.11	1.18±0.31	10.6	2.4	2.3	1.6
**UGT**	***ugt-8***	1±0	14.2±0.68	5.96±0.43	0.3±0.04	2.11±0.16	2.71±1.98	14.2	7.1	6.0	9.1
Lipid-Phosphate-phosphatase	T28D9.3	1±0	2.46±0.17	1.08±0.23	0.59±0.03	1.31±0.24	0.58±0.16	2.5	2.2	1.1	1.0
**CYP450**	***cyp-35C1***	1±0	17.81±1.05	23.73±9.79	0.08±0.03	0.39±0.04	3.97±2.02	17.8	5.1	23.7	51.8
TAG Lipase	F14E5.5	1±0	3.96±0.43	1.68±0.57	0.28±0.02	1.28±0.28	0.56±0.02	4.0	4.5	1.7	2.0
CUB-domain	C29F3.7	1±0	3.57±0.85	1.42±0.49	0.35±0.02	1.01±0.09	0.82±0.25	3.6	2.9	1.4	2.4
UDP-N-AcGlcNac transporter	F15B10.1	1±0	2.54±0.5	1.37±0.46	0.5±0.03	0.8±0.14	0.59±0.2	2.5	1.6	1.4	1.2
C-type lectin	Y19D10A.9	1±0	17.19±10.1	3.59±1.69	0.08±0.01	2.76±1.76	0.82±0.52	17.2	35.4	3.6	10.6
Oxidoreductase	T10B5.8	1±0	13.66±7.55	2.54±1.23	0.23±0.06	2.63±1.35	0.75±0.65	13.7	11.4	2.5	3.2
Zn^2+^-Transporter	T18D3.3	1±0	2.61±0.6	1.14±0.42	0.4±0.06	0.9±0.49	0.49±0.05	2.6	2.2	1.1	1.2
**Reductase**	**F25D1.5**	1±0	11.92±5.78	1.64±0.64	0.89±0.44	1.85±0.11	0.66±0.3	11.9	2.1	1.6	0.7
Cytochrome b5	C31E10.7	1±0	2.37±0.26	1.42±0.52	0.27±0.05	0.36±0.01	0.33±0.1	2.4	1.3	1.4	1.2
FAD-domain	F32D8.12	1±0	2.08±0.42	1.11±0.42	0.21±0.03	0.27±0.05	0.25±0.1	2.1	1.3	1.1	1.2
UGT	*ugt-63*	1±0	3.17±1.8	1.14±0.61	0.01±0	0.01±0.06	0.02±0.01	3.2	17.2	1.1	4.1
Β-Actin	*act-1*	1±0	1±0	1±0	1±0	1±0	1±0				

Values represent fold-inductions of indicated detoxification genes relative to DMSO-treated *control(RNAi)* worms. Each value represents the average±SEM calculated from three independent worm growths and RNA isolations from L4 stage animals, as determined by qPCR; mRNA levels were normalized to *act-1*. Columns on the right indicate the fold-induction by toxins *vs.* DMSO in the same RNAi condition. RNAi depletion of *mdt-15* compromises mRNA expression of select genes; genes whose induction by toxins is compromised at least two-fold are highlighted **bold**.

To test whether MDT-15 is required to induce these detoxification genes, we fed N2 wild-type worms for 40 hr (*i.e.* L1–L4) with control or *mdt-15* RNAi bacteria seeded on plates that contained toxic compounds (fluoranthene or β-naphthoflavone). We chose fluoranthene because it is a naturally occurring carcinogen, and β-naphthoflavone because it is a known inducer of numerous CYP450s [36],[37]. In these worms we then determined the relative mRNA levels of 80 MDT-15 targets by qPCR. We found that fluoranthene and/or β-naphthoflavone induced the mRNA levels of 21 MDT-15 target genes greater than two-fold in *control(RNAi)* worms (Table 3). Importantly, six of these genes exhibited reduced induction by toxins in *mdt-15(RNAi)* worms; for example, fluoranthene induced mRNA levels of *gst-5* ten-fold in *control(RNAi)* worms, but only two-fold in *mdt-15(RNAi)* worms (Table 3). However, not all induced genes (*e.g*. *ugt-25*) exhibited MDT-15 dependent activation, suggesting that other regulatory pathways also contribute to gene induction by toxins. Moreover, when *mdt-15(tm2182)* mutants were exposed to fluoranthene for 40 hr (*i.e.* L1–L4), we observed defects in fluoranthene-induced mRNA accumulation for 13 of the 21 genes, corroborating the results obtained with RNAi (Table 4). The selectivity of MDT-15 action on these 21 detoxification genes is summarized in Figure S3, which demonstrates the weak correlation of toxin induction (particularly the response to fluoranthene) between *control(RNAi)* and *mdt-15(RNAi)* worms (panel A) and wild type and *mdt-15(tm2182)* worms (panel C). Taken together, these data demonstrate that MDT-15 is required not only for basal, but also for toxin-activated transcription of select detoxification genes.

**Table 4:** MDT-15 mutation compromises induction of select detoxification genes by fluoranthene (FLA).

	Strain	Overall fold change *vs.* N2 worms				Fold change caused by toxin *vs.* solvent (within same genotype)	
	Toxin	N2	N2	XA7702	XA7702	N2	XA7702
Function	Gene	DMSO	FLA	DMSO	FLA	FLA *vs.* DMSO	FLA *vs.* DMSO
DUF227	T16G1.6	1±0	3.79±0.36	0.07±0.01	0.48±0.19	3.8	6.9
Hydrolase	F37H8.3	1±0	2.47±0.11	0.17±0.03	0.78±0.22	2.5	4.6
**ADH**	***alh-5***	1±0	7.31±4.04	0.8±0.17	1.68±0.45	7.3	2.1
UGT	*ugt-1*	1±0	3.03±0.6	0.27±0.06	0.47±0.1	3.0	1.7
UGT	*ugt-58*	1±0	3.33±0.5	0.91±0.13	1.7±0.39	3.3	1.9
**UGT**	***ugt-25***	1±0	8.99±1.73	0.83±0.28	2.96±1.07	9.0	3.6
**UGT**	***ugt-13***	1±0	7.32±1.81	0.7±0.09	1.9±0.98	7.3	2.7
**GST**	***gst-5***	1±0	7.68±1.77	0.57±0.08	1.42±0.12	7.7	2.5
**UGT**	***ugt-8***	1±0	17.13±4.76	0.83±0.07	6.29±0.95	17.1	7.6
Lipid-Phosphate-phosphatase	T28D9.3	1±0	2.58±0.02	0.6±0.05	1.11±0.23	2.6	1.9
**CYP450**	***cyp-35C1***	1±0	17.97±6.19	0.28±0	0.81±0.17	18	2.9
TAG Lipase	F14E5.5	1±0	5.88±0.76	1.17±0.13	3.62±1.05	5.9	3.1
**CUB-domain**	**C29F3.7**	1±0	6.1±0.92	0.93±0.12	2.51±0.39	6.1	2.7
UDP-N-AcGlcNac transporter	F15B10.1	1±0	4.86±0.91	1.18±0.13	2.91±0.64	4.9	2.5
**C-type lectin**	**Y19D10A.9**	1±0	8.34±1.76	0.74±0.21	2.81±0.48	8.3	3.8
**Oxidoreductase**	**T10B5.8**	1±0	21.73±12.25	0.65±0.11	4.14±1.21	21.7	6.4
**Zn^2+^-Transporter**	**T18D3.3**	1±0	2.76±0.54	0.84±0.07	1.09±0.05	2.8	1.3
**Reductase**	**F25D1.5**	1±0	4.63±2.96	1.41±0.44	1.62±0.55	4.6	1.1
**Cytochrome b5**	**C31E10.7**	1±0	3.74±0.65	0.51±0.07	0.85±0.2	3.7	1.7
FAD-domain	F32D8.12	1±0	2.46±0.51	0.36±0.06	0.5±0.16	2.5	1.4
UGT	*ugt-63*	1±0	4.16±1.17	0.2±0.08	0.52±0.09	4.2	2.6
Β-Actin	*act-1*	1±0	1±0	1±0	1±0		

Values represent fold-inductions of indicated detoxification genes relative to DMSO-treated N2 worms. Each value is an average±SEM calculated from three independent worm growths and RNA isolations from L4 stage N2 or *mdt-15(tm2182)* animals, as determined by qPCR; mRNA levels were normalized to *act-1*. Columns on the right indicate the fold-induction by toxins *vs.* DMSO within the same genetic background. *mdt-15* mutation compromises expression of select detoxification genes; genes whose induction by toxins is compromised at least two-fold are highlighted **bold**.

Surprisingly, our microarray analysis revealed only three *cyp* genes to be MDT-15 dependent (of a total of 80 *C. elegans cyp* genes encoding CYP450s). This may be attributable, at least in part, to the low expression levels of many *cyp* genes. We therefore used qPCR to examine directly the expression of 43 *C. elegans cyp* genes, and to explore a potential role for MDT-15 in their regulation (Table S6). We found that 14 of these 43 genes required MDT-15 for basal expression in N2 L4 worms. Moreover, 15 of the 43 *cyp* genes were induced greater than two-fold by fluoranthene and/or β-naphthoflavone; this selectivity of *cyp* gene induction suggests that some *cyp* genes may respond to distinct toxins, or may function in roles other than detoxification. Importantly, the induction of nine of 15 toxin-responsive *cyp* genes was strongly reduced in *mdt-15(RNAi)* worms (Table S6, Figure S3B). These results show that MDT-15 is critical for both basal and activated transcription of detoxification CYP450s, thus strengthening the notion that it plays a vital role in the toxin response.

MDT-15 depletion reduces worm viability and causes larval arrest [15], raising the possibility that the reduced capability of *mdt-15(RNAi)* worms to express and appropriately induce detoxification genes is an indirect effect associated with arrested development and premature death. To address this issue, we determined relative mRNA levels of toxin-responsive genes in *mdt-6(RNAi)* worms. We focused on MDT-6 because, (i) like MDT-15, it is a Mediator subunit, (ii) it is required for stage- and gene-specific transcription in *C. elegans*, and (iii) *mdt-6* knockdown evokes larval arrest and premature death, phenotypes reminiscent of but distinct from those of MDT-15 depletion [38],[39]. We found that, in N2 L4-stage worms, *mdt-6* RNAi affected neither basal, nor fluoranthene- or β-naphthoflavone-induced expression of detoxification genes (but did reduce mRNA levels of *fat-6* and *fat-7*, as described [15]), suggesting that MDT-6 is largely dispensable for toxin-induced gene transcription (Table S7, Figure S3A). We conclude that larval arrest and premature death alone are not sufficient to prevent the expression of detoxification genes in response to toxins.

To assess whether the defects in gene induction by toxins reflected abnormal growth of *mdt-15(RNAi)* worms, we employed a conditionally sterile strain, CF512 (*fer-15(b26)II; fem-1(hc17)III*) [40]. To assure that the *fer-1* and *fem-15* mutations do not compromise the detoxification *per se*, we first compared the toxin response in CF512 worms to that in N2 worms. We found that, at the L4 stage, CF512 worms exhibited a very similar response to fluoranthene and β-naphthoflavone as did N2 worms (Table S8). Next, we quantified the mRNA abundance of toxin-responsive MDT-15 targets in CF512 worms exposed to *mdt-15* RNAi only after completion of embryonic and larval development (adult only RNAi). Under these conditions, 12 of the 20 tested genes exhibited MDT-15 dependence in the absence of fluoranthene. Furthermore, 13 genes were fluoranthene-responsive in these worms, and for four of these genes the induction was at least partially MDT-15 dependent, resembling our results obtained in N2 L4 worms (Table S9). Thus, we conclude that the defective detoxification gene expression in *mdt-15(RNAi)* and *mdt-15(tm2182)* worms reflects a direct action of MDT-15, rather than a secondary consequence of reduced viability or arrested development.

### NHR-49, SBP-1, and NHR-8 Are Dispensable for MDT-15 Dependent Expression of Detoxification Genes

Given that MDT-15 binds to, and collaborates with NHR-49 and SBP-1 to express certain metabolic genes, it seemed conceivable that these two regulatory factors might also be responsible for expression of MDT-15 dependent detoxification genes. To test this hypothesis, we knocked down endogenous NHR-49 or SBP-1 in wild-type worms. As previously demonstrated, *nhr-49(RNAi)* and *sbp-1(RNAi)* worms exhibited reduced mRNA levels of *fat-5*, *-6*, and *-7* genes (Table S10). In contrast, these worms were unaffected in their expression of the 20 tested detoxification genes, in unchallenged conditions as well as in the presence of fluoranthene or β-naphthoflavone (Table S10). Indeed, and unlike the pattern exhibited by *mdt-15(RNAi)* worms, the overall induction of detoxification genes by toxins strongly correlated with the induction in *control(RNAi)* worms (Figure S3A). This is noteworthy because *sbp-1* RNAi evokes larval arrest and sterility, further strengthening the notion that developmental arrest *per se* does not impair detoxification. Moreover, worms carrying a mutation in the gene encoding a *C. elegans* NHR implicated in detoxification (*nhr-8*; strain AE501 [*nhr-8(ok186)*]; [41]) also failed to deregulate MDT-15 dependent detoxification genes in basal and xenobiotic-challenged conditions (Table S8, Figure S3C). We conclude that NHR-49, SBP-1, and NHR-8 are largely dispensable for MDT-15 dependent expression of detoxification genes, and that MDT-15 likely uses distinct regulatory factors to control expression of detoxification genes.

### MDT-15 Depletion Increases Toxin Sensitivity

**Figure 1:**
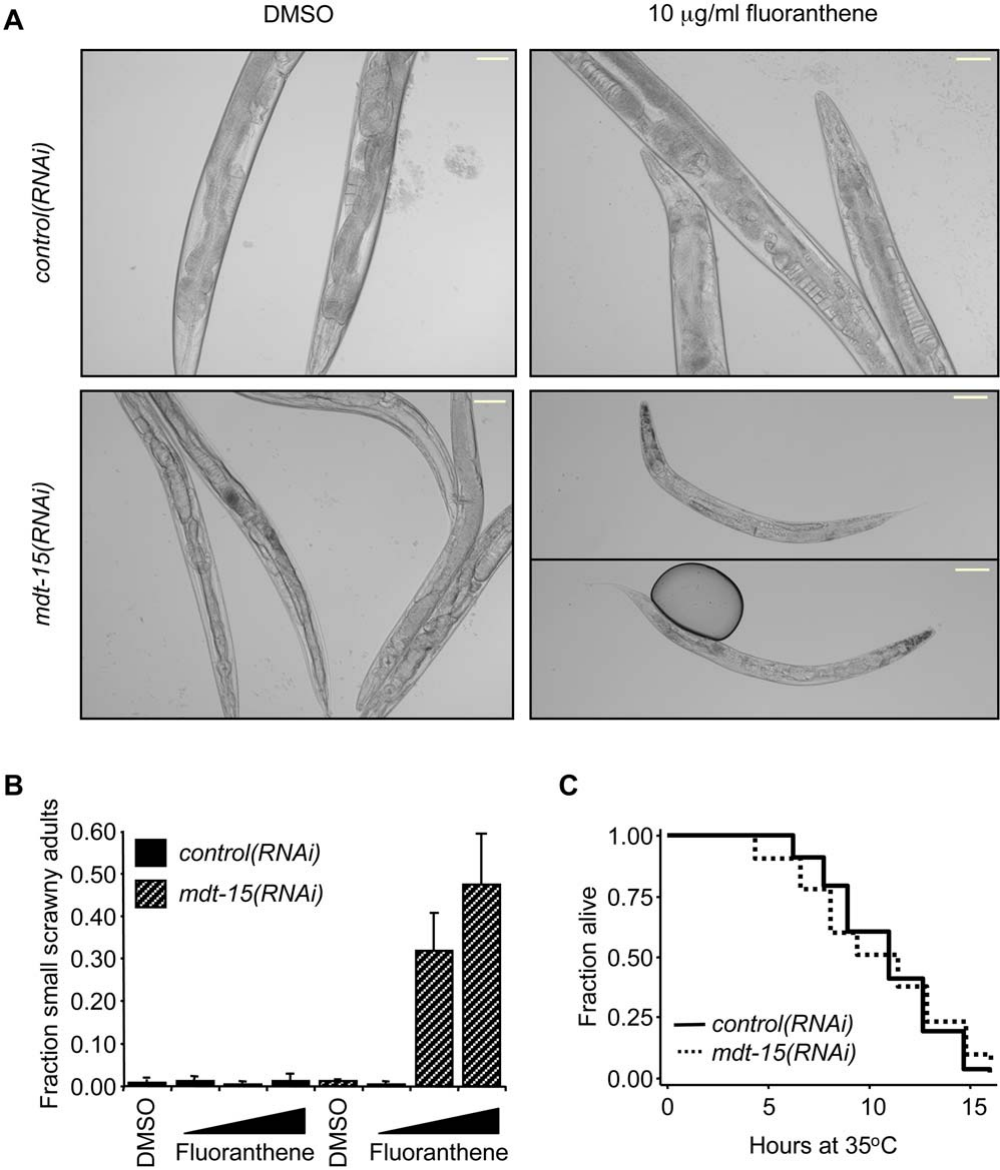
MDT-15 depletion enhances toxin sensitivity, but does not affect thermotolerance. (A) N2 worms were grown on the indicated RNAi bacteria on plates harboring fluoranthene at various concentrations. After four days, animals were scored for morphological defects. Micrographs show representative animals grown on toxin-RNAi combinations, as indicated. The size bar represents 64.5 µm. Exposure of *mdt-15(RNAi)* worms to fluoranthene results in small, scrawny adults; on DMSO, *mdt-15(RNAi)* animals are only slightly thinner than *control(RNAi)* worms. (B) Quantification of adult arrest. Each bar graph represents the fraction of worms scored as arrested; bars represent the average of three individual biological repeats, and errors bars represent the SEM. Fluoranthene was present at 0.4, 2, and 10 µg/ml, respectively, with medium and high concentrations resulting in adult arrest of *mdt-15(RNAi)* but not *control(RNAi)* worms. (C) Survival rate of L4 stage *control(RNAi)* and *mdt-15(RNAi)* worms following exposure to 35°C (starting temperature 20°C). Mean survival time was 11.0 hr for *control(RNAi)* worms (n = 92), and 10.9 hr for *mdt-15(RNAi)* worms (n = 87); *P*-value = 0.22 (log-rank test). The data represent one of five independent experiments with similar outcome, two performed at the L4 stage, and three with day two old adult worms (all exposed to RNAi bacteria from L1 stage on).

As MDT-15 is essential to activate certain detoxification genes, we hypothesized that *mdt-15* depletion or mutation would render worms hypersensitive to toxins. To test this, we grew conditionally sterile CF512 worms on control and *mdt-15* RNAi bacteria (RNAi from the L1 stage on) in the presence of various concentrations of toxins, and monitored their development and morphology until day four of adulthood. After this prolonged exposure to *mdt-15* RNAi, worms were slightly thinner than *control(RNAi)* worms, consistent with previous results [15]. We found that fluoranthene synergized with *mdt-15* RNAi, but not control RNAi, to evoke an arrest as small, scrawny adults (Figure 1A, B). In contrast, this was not the case for β-naphthoflavone (data not shown). Moreover, *mdt-15(tm2182)* mutants exhibited a similar adult arrest phenotype when grown on high concentrations of fluoranthene (Figure S2), whereas wild-type N2 worms showed only a mild developmental delay at the same concentrations of fluoranthene. We conclude that compromised detoxification capability as a result of reduced MDT-15 function causes growth defects in worms challenged with select toxic compounds.

### MDT-15 Is Essential for the Transcriptional Response to Heavy Metals

**Figure 2:**
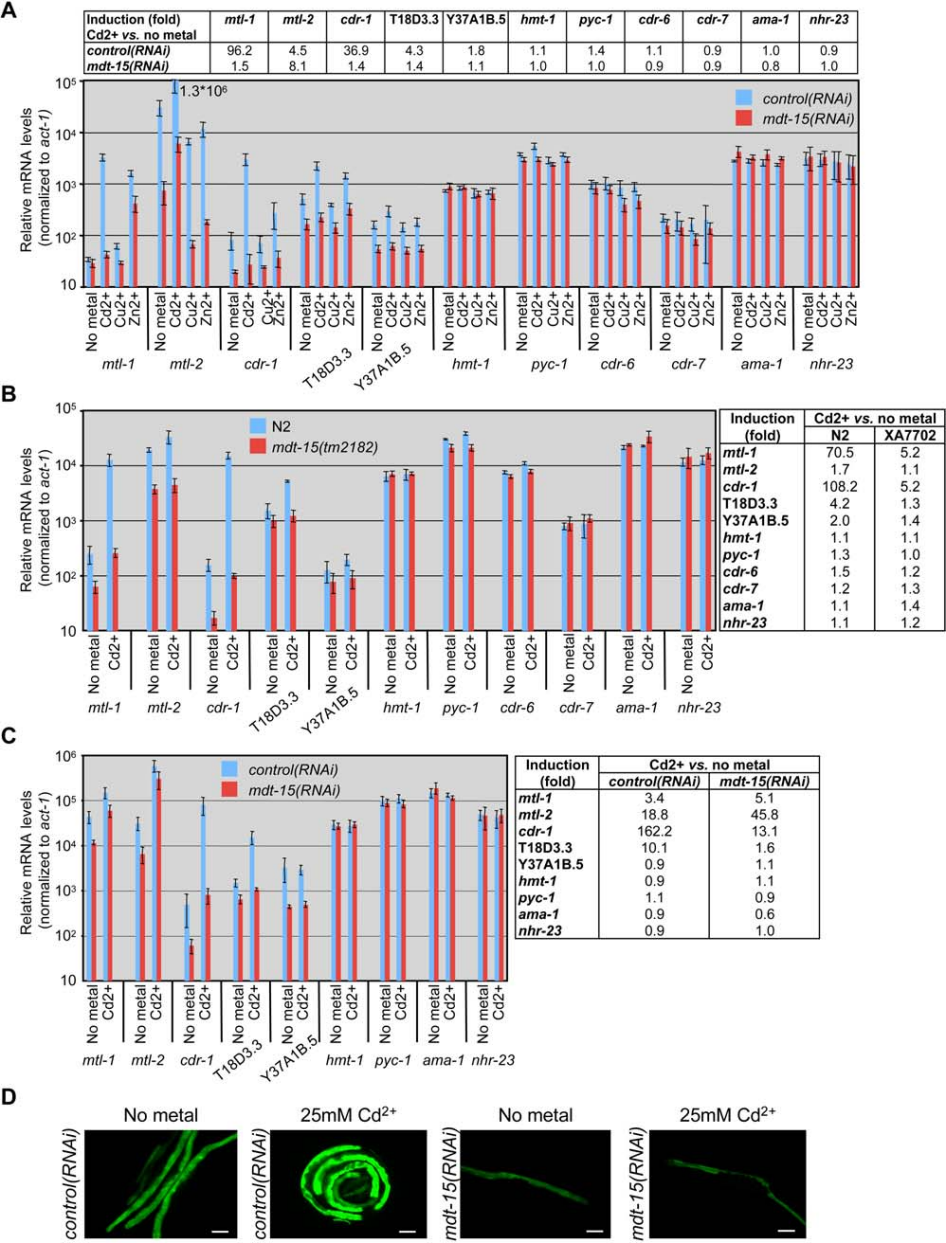
MDT-15 is essential for mRNA induction of heavy metal-induced genes. (A–C) Relative mRNA accumulation (determined by qPCR quantification) from select metal responsive genes in worms grown in the presence of metal ions (as indicated, either 25 µM Cd^2+^, 100 µM Zn^2+^, or 10 µM Cu^2+^). Each bar graph represents the average relative mRNA level from three or more independent worm growths and mRNA isolations; mRNA levels are normalized to *act-1* mRNA. The error bars represent SEM. Note that the scale is logarithmic. Tables above or next to the panels indicate the fold-induction by Cd^2+^ *vs.* no metal in the same genetic condition. RNAi depletion (A, C) or mutation of *mdt-15* (B) compromised mRNA expression of select metal detoxification genes, whereas expression of the control genes *hmt-1* (heavy metal tolerance factor), *pyc-1* (pyruvate carboxylase), *cdr-6*, *cdr-7* (cadmium responsive genes), *ama-1* (Pol_II_, large subunit), and *nhr-23* (nuclear hormone receptor) were unaffected by *mdt-15* depletion or mutation. (A) mRNA abundance in N2 L4 stage animals fed *control* RNAi (blue) or *mdt-15* RNAi (red). (B) mRNA abundance in L4 stage N2 (blue) or *mdt-15(tm2182)* (red) animals. (C) mRNA abundance in day two old sterile adult animals (CF512) exposed to *control* RNAi (blue) or *mdt-15* RNAi (red). (D) Fluorescence micrographs of adult worms carrying an *mtl-2*::GFP promoter fusion. Five hr exposure to Cd^2+^ increased the GFP signal worms fed with control RNAi, but not with *mdt-15* RNAi bacteria. The size bar represents 32.3 µm.

Based on their molecular identity or previous studies, the MDT-15 targets *cdr-6*, *mtl-2,* T18D3.3, and Y37A1B.5 are predicted to contribute to heavy metal detoxification [42]–[45]. Interestingly, transcription of *mtl-2* is induced by cadmium (Cd^2+^) and zinc (Zn^2+^), but not by copper (Cu^2+^) [44],[45]. Thus, we tested whether the previously uncharacterized genes Y37A1B.5 and T18D3.3 are metal-responsive also. We found that, whereas the Y37A1B.5 was not strongly induced by any of the tested metals, T18D3.3 was induced three- to four-fold by Cd^2+^and Zn^2+^, but not by Cu^2+^, thus establishing it as a novel metal-responsive gene (Figure 2A).

Given that two of the four metal detoxification genes are metal-responsive, MDT-15 might contribute both to basal and to metal-induced expression of these genes. This hypothesis is supported by the fact that the list of MDT-15-dependent genes (as determined by microarray analysis) overlaps in statistically significant manner with a list of Cd^2+^-responsive genes ([46]; Table S11). To further characterize MDT-15's possible role in metal detoxification, we fed wild-type N2 worms for 40 hr (*i.e.* L1–L4) with control or *mdt-15* RNAi bacteria seeded on plates containing Cd^2+^, Zn^2+^ or Cu^2+^. We then probed for induction of the aforementioned genes, and other known heavy metal responsive genes (*cdr-1*, *mtl-1*), by qPCR. Consistent with published results, we observed mRNA accumulation of *cdr-1*, *mtl-1*, and *mtl-2* in the presence of Cd^2+^, and of *mtl-1* and *cdr-1* in the presence of Zn^2+^ (Figure 2A). Importantly, the Cd^2+^- and Zn^2+^-dependent induction of *mtl-1, mtl-2, cdr-1,* and T18D3.3 was reduced in *mdt-15(RNAi)* worms (Figure 2A). Moreover, we found that *mdt-15(tm2182)* mutants were also severely defective for both basal and Cd^2+^-induced *mtl-1*, *mtl-2*, *cdr-1*, and T18D3.3 expression, and for basal Y37A1B.5 expression (Figure 2B). Finally, in conditionally sterile worms, adult-only *mdt-15* RNAi resulted in reduced basal *mtl-1*, *mtl-2*, *cdr-1*, T18D3.3 and Y37A1B.5 mRNA levels and in reduced Cd^2+^-dependent accumulation of *cdr-1* and T18D3.3 mRNA (Figure 2C). Together, these data demonstrate that MDT-15 plays a critical role in the transcriptional response to heavy metals in *C. elegans*.

In the above experiments, worms were exposed for ∼40 hr to heavy metals, corresponding to chronic conditions. In order to test an acute response to heavy metal, we grew adult worms for 48 hr on control and *mdt-15* RNAi, and then challenged them with high concentrations of Cd^2+^ for five hr. We used a worm strain harboring a transcriptional P_mtl-2_::GFP reporter, CL2122 [47]. This allowed us to rapidly assess *mtl-2* promoter activity *in vivo*. Reminiscent of the results obtained with chronic metal exposure, Cd^2+^ caused induction of GFP-fluorescence. Importantly, the GFP signal was weaker in worms grown on *mdt-15* RNAi, and Cd^2+^-dependent induction was not observed in these worms (Figure 2D). Thus, MDT-15 is apparently essential for both chronic and acute Cd^2+^ induced *mtl-2* transcription. Moreover, MDT-15 was critical to induce *mtl-2* promoter activity specifically in intestinal cells, further supporting the hypothesis that MDT-15 is particularly critical for intestinal gene expression.

### MDT-15 Is Dispensable for the Heat-Shock Response in C. elegans

**Figure 3:**
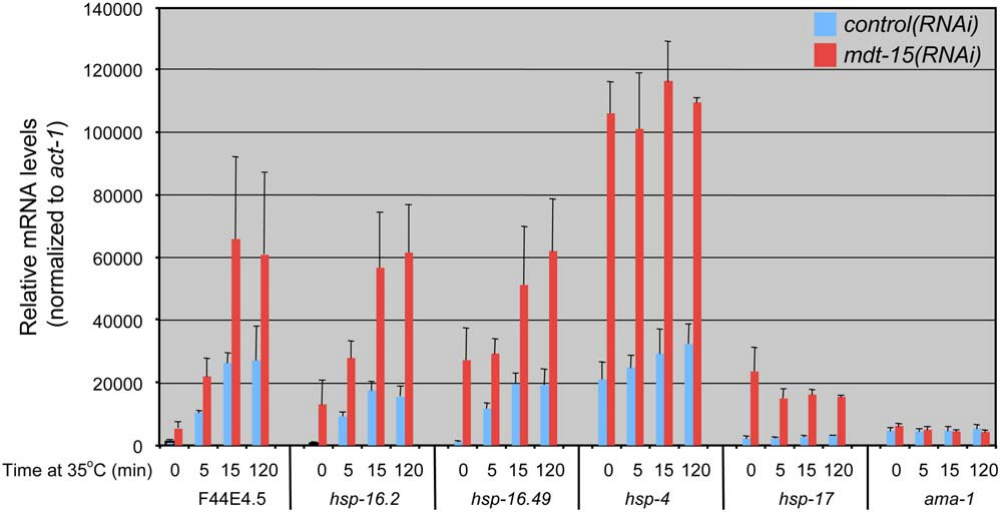
MDT-15 is dispensable for the heat-shock response. mRNA accumulation from select heat-shock protein genes in L4-stage *control(RNAi)* worms (blue) and *mdt-15(RNAi)* worms (red) exposed to 35°C for various times, as indicated. Each bar represents the average relative mRNA level of the indicated gene (average from three independent worm growths and RNA isolations from N2 L4 stage animals). Relative mRNA levels are normalized to *act-1* mRNA levels; the error bars represent SEM.

We have previously reported that a specific nutritional condition, short-term fasting, relies on MDT-15 to induce select genes [15]. This raises the possibility that MDT-15 represents a general stress coregulator. However, our microarrays revealed that *mdt-15(RNAi)* worms upregulate known and putative heat-shock proteins (*hsp-17* and *hsp-16.2* are α-crystallins, T05E11.3 is related to HSP90). Thus, in contrast to the aforementioned ingestion-related stresses, MDT-15 might play a negative regulatory role in heat-induced transcription. To test this possibility we grew wild-type N2 worms for 40 hr (*i.e.* L1–L4) on control and *mdt-15* RNAi bacteria at ambient temperature (20°C), subsequently applied heat-shock (*i.e.* five min, 15 min, and two hr at 35°C), and then quantified the mRNA levels of several *hsp* genes. At 20°C *mdt-15(RNAi)* worms exhibited increased mRNA levels of T05E11.3, *hsp-16.2*, and *hsp-17* compared to *control(RNAi)* worms; we detected similar increases for the mRNAs of the HSPs F44E5.4 (HSP70), *hsp-16.1,* and *hsp-16.49* (Figure 3). Heat-shock failed to affect *hsp-4*, *hsp-17*, and T05E11.3 mRNA levels, but induced F44E5.4, *hsp-16.1, hsp16.2,* and *hsp-16.49* mRNA levels in *control(RNAi)* worms (Figure 3 and data not shown); in contrast, mRNA accumulation was not blocked in *mdt-15(RNAi)* worms. Instead, *mdt-15(RNAi)* worms induced these genes somewhat above the levels exhibited by *control(RNAi)* worms. Moreover, *mdt-15(tm2182)* mutants exhibited a qualitatively similar, yet milder gene expression defect (Figure S4). Thus, our data indicate that MDT-15 is dispensable for heat-shock gene activation, and that it instead suppresses expression of select *hsp* genes. We do not fully understand the upregulation of both basal and induced expression of these genes upon *mdt-15* depletion or mutation, but it seems likely that this is an indirect effect, and that MDT-15 does not act equivalently in all stress responses. Indeed, the increased *hsp* expression may be an indirect consequence of the detrimental effects of *mdt-15* RNAi (which evokes growth arrest and death) rather than a direct effect of deregulation of *hsp* gene modulators. Taken together, these results demonstrate that MDT-15 is not generally required for stress-activated gene expression, but rather may constitute an element in a regulatory system that specifically monitors availability and integrity of ingested material (see Discussion).

As *mdt-15* depletion and mutation cause increased expression of heat-shock protein genes, we hypothesized that they might render animals resistant to high temperatures. To test this hypothesis we assessed the survival rate in *control(RNAi)* and *mdt-15(RNAi)* worm populations at 35°C. We found that both populations exhibited similar thermotolerance phenotypes. This was the case in N2 worms exposed to *mdt-15* RNAi bacteria from L1–L4 stage (Figure 1C), and also in two-day old conditionally sterile worms exposed to *mdt-15* RNAi from the L1 stage on (data not shown). Therefore, the altered heat-shock protein gene expression following *mdt-15* depletion is not sufficient to increase resistance to elevated temperatures. This may be due to other gene expression defects in *mdt-15(RNAi)* worms. Alternatively, abnormally high heat-shock protein expression in *mdt-15(RNAi)* worms may not confer a marginal benefit over heat-shock protein in *control(RNAi)* animals.

## Discussion

A prevailing view concerning transcriptional regulation is that DNA-binding regulatory factors are the primary determinants of specificity and coordination within transcriptional regulatory networks. Our results support an additional mechanism in which a specific component of the Mediator complex serves such functions. Specifically, we demonstrate that the *C. elegans* Mediator subunit MDT-15 specifies a portion of the alternative metabolic responses to a complex mixture of ingested material.

### MDT-15 Affects Systemic Detoxification Pathways

Like most soil-dwelling animals *C. elegans* experiences a diversity of conditions, such as insufficient nutrient supply, potentially detrimental substances (endo- and xenobiotics), and harmful microorganisms. Toxic hydrophobic compounds and heavy metals represent a complex challenge as they can produce their adverse effects both acutely, and during chronic accumulation over prolonged exposure periods. Therefore, efficient detoxification mechanisms have evolved to ensure metabolism and elimination of harmful molecules. Here, we provide several lines of evidence that the conserved Mediator subunit MDT-15 is a key contributor towards regulation of systemic detoxification. First, even in the absence of toxic challenge, *mdt-15* depletion or mutation results in downregulation of many detoxification-related genes. In addition, MDT-15 is required to induce select detoxification genes in response to hydrophobic toxins and heavy metals. Finally, depletion or mutation of MDT-15 renders worms hypersensitive to at least one toxic compound. Of note, MDT-15 affects expression of all three classes of detoxification genes, *i.e.* activation enzymes such as CYP450s (class I), transferase enzymes such as the UGTs and GSTs (class II), and transporters such as *pgp-7* and *pmp-5* (class III [48]). This highlights MDT-15's broad influence on detoxification and suggests that MDT-15 coordinates entire gene expression programs in response to individual toxic challenges. These data likely reflect a direct contribution by MDT-15, and not an indirect feature of sick/arrested *mdt-15(RNAi)* worms, because (i) larvally arrested *mdt-6(RNAi)* and *sbp-1(RNAi)* worms did not deregulate detoxification genes, (ii) the heat-shock response was unaffected, suggesting that stress-driven transcription can function, and (iii) numerous fluoranthene and cadmium-induced genes were also deregulated in worms exposed to *mdt-*15 RNAi only after the completion of larval development. Taken together, our data strongly suggest that MDT-15 critically contributes to at least two detoxification pathways in *C. elegans*.

### MDT-15 May Integrate Regulation of Xenobiotic Detoxification

MDT-15 integrates expression of metabolic genes by interacting with at least two metabolic regulatory factors, NHR-49 and SBP-1 [15],[16]. Our findings suggest that MDT-15 may similarly integrate the transcriptional response to environmental stressors such as xenobiotics and heavy metals by employing a different set of regulatory factors. Indeed, MDT-15, and not any single regulatory factor, may constitute the key determinant of the detoxification response in *C. elegans*. Analogous to metabolic regulation, MDT-15 may collaborate with NHR family regulatory factors to induce specific detoxification programs. Strikingly, the *C. elegans* genome is lavishly equipped with candidate NHRs [49]. Moreover, NHRs figure prominently in detoxification in metazoans [25], [41], [50]–[52]. In mammals, the NHRs PXR and CAR are particularly critical: by binding structurally diverse compounds [53], they induce expression of appropriate detoxification genes. Notably, both NHRs target the Mediator subunit MED1, which is necessary for induction of detoxification genes in response to drugs and/or toxins [9],[10]. Given that MDT-15 associates with NHRs [15], and modulates transcription in response to xenobiotic compounds (this study), hMED15, the human MDT-15 ortholog, may also contribute to systemic detoxification by CAR and PXR.

### A “Specific” Role for MDT-15: Implications for Mediator Structure and Function

As the Mediator complex contributes to most Pol_II_ transcription, a simple view is that it might function through a single functional domain, or alternatively, that it may somehow operate with little specificity. Instead, the emerging view is that individual Mediator subunits appear to exhibit restricted specificity: For example, in *D. melanogaster* S2 cells, MED23 is specifically required for heat-induction of *hsp26*, whereas MED16 is critical to upregulate genes in response to LPS [6]. Similarly, serine 208 phosphorylation of yeast MED2 controls expression of select genes assuring growth under low-iron conditions [54]. More remarkably, our findings demonstrate that a single Mediator subunit appears to “route” transcription, selecting a physiological output appropriate for a given input, in this case energy metabolism in response to nutrient ingestion, or detoxification/transport in response to xenobiotic or heavy metal ingestion. To do so, MDT-15 likely interacts with a spectrum of sequence-specific regulators, as well as particular factors that directly or indirectly control polymerase activity. By incorporating this routing function, MDT-15 takes on a physiological scope that is broader and more sophisticated than any specific regulatory factor. Moreover, for an individual Mediator subunit to communicate with distinct regulatory factors and transcription components under different conditions (while being dispensable in still other circumstances), implies that it may itself receive signaling inputs that drive these distinct behaviors. Therefore, although the composition of Mediator may be similar in all cells and conditions, mere presence of a certain subunit within Mediator may not reflect its specific contributions to regulatory action. Indeed, it seems likely that many Mediator subunits may be subject to various post-translational modifications that could affect their functions. It would be interesting to assess systematically the relative mechanistic contributions of MDT-15 to Mediator's function under different physiologic conditions.

### Regulatory Crosstalk between Detoxification and Energy Metabolism through Mdt-15

The apparent routing between detoxification and energy metabolism via MDT-15 is notable as others have found that contaminants such as cadmium affect the expression of energy metabolism genes [44],[46],[55]. Although deregulation of metabolic genes may result from perturbed signal transduction [56],[57], regulation of these two processes might conceivably have evolved in a coordinated manner. For example, responses to toxins may impose increased energy expenditure, as detoxification enzymes use ATP, NADH and other energy carriers as co-factors. Similarly, short-term fasting and long-term starvation could cause accumulation of harmful metabolic side-products that necessitate expression of detoxification genes. Accordingly, modulating the activity of a factor such as MDT-15 could ensure appropriate gene expression. Such functional regulation has been described for the mammalian PPARγ-coactivator 1 (PGC-1), which is upregulated by stimuli such as fasting, exercise, and cold exposure. Upon this induction, PGC-1 collaborates with several regulatory factors to confer a switch from reductive to oxidative metabolism [50],[58]. Similarly, detailed characterization of the network that defines MDT-15 action will provide new insights into metabolic homeostasis.

### MDT-15 Affects Energy Metabolism and Detoxification – Implications for Aging

It is intriguing that many of the biological processes susceptible to MDT-15 regulation are linked to aging. We previously found that MDT-15 depletion dramatically shortens *C. elegans* life span, a phenotype partially attributable to reduced FA desaturation [15]. In our present study, we demonstrate a role for MDT-15 in regulation of detoxification, suggesting that compromised toxin elimination might also contribute to the short life span of these animals. Intriguingly, the expression of several genes that we identified as MDT-15 targets is affected by mutations that alter worm life span [59],[60]; moreover, stress tolerance is believed to contribute to life span extension in *C. elegans* [61],[62]. In summary, we speculate that MDT-15 is an integral part of a regulatory system that coordinates systemic adaptation to ingestion-related stress, thus ensuring maintenance of health and longevity.

### MDT-15 Participates in Routing Ingestional Responses

**Figure 4:**
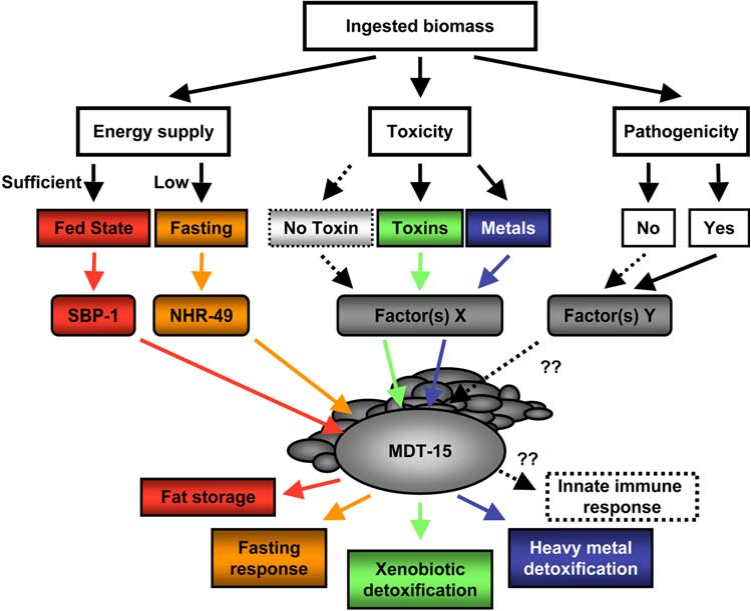
A model for MDT-15 function. Model depicting the role of MDT-15 in monitoring availability and quality of ingested material. Note that in each case, we propose that MDT-15 confers regulatory effects both in “unchallenged” (sufficient energy supply, no xenobiotics or metals, no pathogens) and “challenged” (fasting, xenobiotic or metal exposure, pathogenic infection) conditions. For further explanations, see text.

MDT-15 affects expression of energy metabolism genes that respond to food supply [15],[27], as well as detoxification genes that respond to environmental contaminants (this study); in *C. elegans,* both activities are directly linked to eating. Although we cannot rule out that some toxins may be absorbed through the worm cuticle, this barrier is generally impervious [63]; moreover, metals are primarily taken up by feeding [64]. Thus, we suggest that MDT-15 is a component of an ingestion-related control system that monitors both the energy availability, as well as the integrity of the ingested material (Figure 4). Such a control system may be particularly beneficial for the soil-dwelling *C. elegans*, as its feeding during the growth periods may be accompanied by unavoidable co-ingestion of unfavorable substances. As worms may be unable to evade potential harm, or physically separate detrimental compounds, the need for an efficient defense system arises. We speculate that MDT-15 might be an active component of such a regulatory network. Accordingly, in fed worms MDT-15 may cooperate with SBP-1 to drive fat storage and adipogenesis [16], whereas in fasted worms MDT-15 would collaborate with NHR-49 to efficiently cope with short-term fasting [15]. In feeding worms, this same system might invoke “quality control”: hence, MDT-15 and yet unidentified regulatory factors would implement expression of proteins that appropriately metabolize and/or eliminate harmful xenobiotic substances. Moreover, MDT-15 targets such as lectins, GSTs, and lipid metabolism genes have been implicated in the response to pathogen exposure [28], suggesting that MDT-15 may also participate in host defense against microbial infection, another process resident in the worm gut [31]. In summary, we propose that MDT-15 is an essential component of a regulatory network that governs screening and routing of the ingested material to achieve its appropriate utilization, metabolism, and elimination (Figure 4).

## Materials and Methods

### Nematode Strains and Growth Conditions

*C. elegans* strains N2-Bristol (WT), CF512 [*fer-15(b26)II; fem-1(hc17)III)*] [40]), and CL2122 *dvIs15* [*mtl-2*::GFP, pPD30.38 (*unc-54* expression vector)] [47], and AE501 (*nhr-8(ok186)* [41]) were maintained as described [65]. The strain XA7702 was generated by out-crossing *mdt-15(tm2182)* worms (a gift from Dr. S. Mitani, Tokyo Women's Medical University) four times with wild-type N2 worms; after isolating homozygotes we verified the presence of the original genomic deletion by PCR with the following primers: 5′-aatgttgctgctcaacgtgc-3′ (forward primer), and 5′-cgatctcttccaattggtcc-3′ (reverse primer). For total mRNA isolation, worm embryos were allowed to hatch on unseeded nematode growth media (NGM)-lite plates overnight at 20°C, and then grown from L1–L4 stages (for 40 hr) on NGM-lite plates containing 25 µg/mL carbenicillin, 4 mM IPTG (Alexis 582-600), and 12.5 µg/mL tetracycline seeded with the appropriate RNAi bacteria. For validation of candidate MDT-15 targets, we grew worms as follows: control RNAi for 36 hr, control RNAi 24 hr followed by *mdt-15* RNAi for 12 hr, control RNAi 12 hr followed by *mdt-15* RNAi for 24 hr, or *mdt-15* RNAi for 36 hr. Toxins were dissolved in DMSO and added at the following final concentrations (unless indicated otherwise): fluoranthene (Sigma F4418) at 5 µg/ml, and β-naphthoflavone (Sigma N3633) at 6 µg/ml. Heavy metals were dissolved in water and added at the following final concentrations: 3CdSO_4_*8H_2_O (Sigma C3266) at 25 µM, ZnCl_2_ at 100 µM, and CuSO_4_ at 10 µM. Life span and thermotolerance assays were performed as described [66],[67].

### RNAi Constructs

The *mdt-15*, *mdt-6*, *nhr-49* and *sbp-1* RNAi clones have been described [68],[69].

### DIC and Fluorescence Microscopy

Worms were grown on NGM-lite RNAi plates seeded with *E. coli* strain HT115 carrying the appropriate RNAi vector. Worms were transferred onto 2% (w/v) agarose pads for microscopic examination. We captured images on a Retiga EXi Fast1394 CCD digital camera (QImaging, Burnaby, BC, Canada) attached to a Zeiss Axioplan 2 compound microscope (Zeiss Corporation, Jena, Germany), and used Openlab 4.0.2 software (Improvision, Coventry, U.K.) for image acquisition.

### Microarrays and Data Analysis

Starting with developmentally synchronized L1 stage larvae, N2 worms were grown on control or *mdt-15* RNAi for 36 hr to yield synchronized L4 stage populations. Notably, this RNAi protocol did not result in visible death or sickness at the very stage worms were harvested for RNAi isolation. L4 larvae were harvested, washed 5 times in M9, and frozen in liquid nitrogen. RNA was isolated by the Trizol method [69], labeled with Cy3 or Cy5, and hybridized to the WUSTL *C. elegans* microarrays. We performed five independent biological repeats; one of the arrays was a dye swap. We extracted spot intensities with SpotReader (Niles Scientific), using the default parameters to flag bad spots. To normalize the data, we used RMA background correction and the “NormalizeWithinArrays” function of limma with the print tip loess correction [19]. For a gene to be considered in the final analysis, it had to be unflagged and have a background-subtracted intensity (mean of red and green channels) greater than 64 on at least three of the five arrays. These criteria are non-stringent, particularly the intensity criterion, and may have introduced some false positives, but enabled us to capture expression changes in genes with relatively low expression levels. We used empirical Bayes fitting in limma with multiple hypothesis correction method “BH”, and considered genes with a *P*-value<0.05 to be differentially expressed [18]. The microarray data have been deposited in the Gene Expression Omnibus database (http://www.ncbi.nlm.nih.gov/geo/) under the accession number GSE9720.

To determine overlaps between our set of MDT-15 dependent genes and the set of Cd^2+^-responsive genes [46], we proceeded as follows: As only 10,909 genes pass our stringent spot quality filter in the array analysis, we used this number to estimate the significance of the overlap. This produces a more conservative *P*-value than using the entire set of genes on the array. The expected fraction of genes that overlap is the fraction of MDT-15 regulated genes multiplied with the fraction of Cd^2+^-reponsive genes. We used the binomial distribution to calculate statistical significance [70].

### Preparation of Total Nematode mRNA and qPCR Analysis

Isolation, purification, and reverse transcription of *C. elegans* RNA have been described ([69] and http://www.ucsf.edu/krylab/Stefan-WormRNA_isolationqPCR.pdf). QPCR was performed in an ABI7300 PCR machine, and the data analyzed using the Ct method (Applied Biosystems Prism 7700 Users Bulletin No. 2 http://docs.appliedbiosystems.com/pebiodocs/04303859.pdf). Relative mRNA levels were normalized to *act-1* mRNA levels. Primers for qPCR were designed using Primer3 [71]. Primers were tested on dilution series of cDNA, and analyzed for PCR efficiency [72]; primer sequences are available on request.

## Supporting Information

Figure S1 Flowchart outlining identification and confirmation of new MDT-15 targets, and overlap between genes deregulated following MDT-15 depletion vs. mutation.
(A) The flowchart depicts the workflow regarding identification (by microarrays) and confirmation (by qPCR) of MDT-15 targets. Validation by qPCR was performed in both *mdt-15(RNAi)* worms and *mdt-15(tm2182)* mutants. (B) Overlap and separation of genes deregulated in *mdt-15(RNAi)* worms and *mdt-15(tm2182)* mutants.
(3.99 MB TIF)

Figure S2 Worms carrying a mutation in the MDT-15 gene exhibit short lifespan, reduced brood size, and toxin hypersensitivity.
(A) Structure of the MDT-15 gene, and its three mRNA and two protein products. For mRNAs, non-coding exons are black, coding exons are white and introns are indicated as lines. The box labeled *tm2182* indicates the location of the genomic *tm2182* mutation. For protein products, light grey indicates the ORF, with the dark grey box highlighting the KIX domain. The *tm2182* mutation is predicted to result in the production of a truncated protein, as indicated. (B) Life span analysis of wild-type N2 and mutant *mdt-15(tm2182)* worms reveals short life span of the mutant worms. Mean life spans (measured in days after reaching adulthood) were 19.7 for N2 worms (filled line; n = 78), and 13.7 days for *mdt-15(tm2182)* mutants (dotted line; n = 76); P-value <0.0001 (log rank test). The data shown here were obtained with worms cultured at 20°C; similar results were obtained when worms were grown at 25°C (data not shown). (C) Total brood count analysis of wild-type N2 and mutant *mdt-15(tm2182)* worms reveals reduced brood size of the mutant worms. The *mdt-15(tm2182)* mutants gave rise to fewer eggs, and most eggs failed to hatch. Total brood size for mutant worms is the sum of unhatched eggs and larvae. Bar graphs represent the total number of progeny±SEM (n = 17 individual worms). (D) N2 and *mdt-15(tm2182)* worms were grown on plates harboring fluoranthene at 10 µg/ml. After four days, animals were scored for developmental defects. Micrographs show representative animals grown on toxin or DMSO (solvent), as indicated. The size bar represents 64.5 µm. Exposure of *mdt-15(tm2182)* worms to fluoranthene results in small, scrawny adults; on DMSO, *mdt-15(tm2182)* animals are only slightly thinner than N2 worms.
(5.81 MB TIF)

Figure S3. MDT-15 is required to induce select detoxification genes.
The scatter plots depict pairwise comparisons of detoxification gene inductions by fluoranthene or β-naphtoflavone in different worm strains. Each data point represents the log of the fold-induction of an individual gene in *control(RNAi)* (A,B) or wild-type N2 worms (C) on the X-axis vs. the log of the fold-induction of the same gene in worms exposed to the indicated RNAi bacteria (A,B), or in mutant worms (C) on the Y-axis. R-values indicate the correlation coefficients between the two samples. Panel A represents the detoxification genes characterized in Tables 3, S7, and S10. Panel B represents the 15 toxin-inducible cyp genes described in Table S6. Panel C represents the detoxification genes described in Tables 4 and S8. Correlation is generally poorer between wild-type/*control(RNAi)* worms and worms with mutated or depleted MDT-15 (R<0.6 for fluoranthene exposure) than between wild-type/*control(RNAi)* worms and worms with either mutated or depleted MDT-6, NHR-49 or NHR-8 (R>0.8 for fluoranthene exposure).
(4.30 MB TIF)

Figure S4 MDT-15 is dispensable for the heat-shock response.
Quantification of the mRNA levels of select heat-shock protein genes in L4-stage N2 worms (blue) and *mdt-15(tm2182)* worms (red) exposed to 35oC for various times, as indicated. Each bar represents the average relative mRNA level of the indicated gene (average from three independent worm growths and RNA isolations from N2 L4 stage animals). Relative mRNA levels are normalized to act-1 mRNA levels; the error bars represent SEM.
(2.89 MB TIF)

Table S1 List of genes deregulated in *mdt-15(RNAi)* worms (compared to *control(RNAi)* worms).
The data represent the output of “limma” analysis from five independent mRNA isolations and microarray hybridizations. “ID” refers to the identity of individual spots on the arrays, “M” represents the log of the fold change, “A” represents the averaged spot intensity, and “Function” refers to the predicted or actual function according to WormBase (WS157). In the “Name” column, detoxification related genes are bold; previously recognized MDT-15-dependent genes are italic; HSP-like proteins are underlined. Table S1A lists genes downregulated in *mdt-15(RNAi)* animals, whereas Table S1B lists genes upregulated in *mdt-15(RNAi)* animals. Genes are ordered by highest statistical significance of over/underexpression in *mdt-15(RNAi)* animals vs. *control(RNAi)* animals.
(0.59 MB DOC)

Table S2 Validation of candidate MDT-15 targets.
QPCR quantification of mRNA levels of 85 candidate MDT-15-dependent genes. Values represent fold changes±SEM in *mdt-15(RNAi)* worms vs. *control(RNAi)* worms, calculated from the average relative mRNA levels from three independent biological replicates (mRNA levels normalized to act-1). Genes whose MDT-15 dependence (as judged from the microarray experiments) was confirmed by this qPCR analysis are in bold (a gene was classified as MDT-15-dependent if its mRNA level was reduced at least two-fold at any of the three time points). The control genes ama-1 and nhr-23 are not MDT-15 dependent. Note that genes are classified into groups with similar biological functions (left column).
(0.20 MB DOC)

Table S3 Validation of candidate MDT-15 targets in mutant *mdt-15(tm2182)* worms.
QPCR quantification of mRNA levels of 97 known and candidate MDT-15-dependent genes. Values represent fold changes±SEM in mutant *mdt-15(tm2182)* worms vs. N2 worms, calculated from the average relative mRNA levels from three independent biological replicates (mRNA levels normalized to act-1). 50 genes downregulated more than two-fold in *mdt-15(tm2182)* worm are in bold. The hacd-1 and cpt-3 genes are upregulated in mutant *mdt-15(tm2182)* worms, whereas MDT-15 itself and control genes ama-1 and nhr-23 are not significantly regulated, consistent with data from our previous study [14]. Genes are classified into groups with similar biological functions (left column).
(0.18 MB DOC)

Table S4 Occurrence of gene families and protein domains in microarray results based on GO terms.
Occurrence of gene ontology terms and protein domains (based on InterPro annotation) in genes that, by microarray analysis, were found to be upregulated in *mdt-15(RNAi)* worms (vs. *control(RNAi)* worms), ranked by lowest P-value. 100 genes (of 120 total) were not classified by gene functional category analysis; 79 genes were not classified by the protein domain analysis. “Count” indicates the number of genes amongst MDT-15 targets that fit the respective term. “%” indicates the percentage of these amongst all MDT-15 targets. “P-value” indicates the statistical significance of the overrepresentation of an individual category; we used a P-value of 0.05 as cutoff.
(0.05 MB DOC)

Table S5 Many genes downregulated after MDT-15 depletion are intestine-enriched.
Comparison of genes downregulated after MDT-15 depletion (as determined by microarray analysis) and groups of genes expressed in tissue-restricted manner. Columns contain WormBase accession numbers of MDT-15 target genes with respective tissue specific expression. Intestine-, muscle-, and germline-enriched genes are from Pauli et al. [31], and pharynx-enriched genes from Gaudet et al. [32].
(0.09 MB DOC)

Table S6 Basal and toxin-induced expression of some *cyp* genes (encoding CYP450s) is reduced in *mdt-15(RNAi)* worms.
QPCR quantification of mRNA levels of cyp genes. Values represent fold changes±SEM in *mdt-15(RNAi)* worms vs. *control(RNAi)* worms, calculated from the average relative mRNA levels from three independent biological replicates (mRNA levels normalized to act-1). FLA = fluoranthene; NF = β-naphthoflavone; D = DMSO. Columns on the right indicate the fold-induction by toxins vs. DMSO in the same genetic condition; several genes exhibit reduced fold-induction upon MDT-15 depletion. cyp genes whose expression is toxin-induced are listed in the top section, unresponsive cyp genes in the middle section; in both sections, bold font indicates MDT-15 dependence. Note that some genes are MDT-15-dependent for basal and induced transcription.
(0.16 MB DOC)

Table S7 Expression of toxin-induced MDT-15 targets is largely unaffected in mdt-6(RNAi) worms.
QPCR quantification of mRNA levels of MDT-15-dependent detoxification genes. Values represent fold changes±SEM in mdt-6(RNAi) worms vs. *control(RNAi)* worms, calculated from the average relative mRNA levels from three independent biological replicates (mRNA levels normalized to act-1). FLA = fluoranthene; NF = β-naphtoflavone.
(0.08 MB DOC)

Table S8 The toxin response is largely unaffected in L4 stage CF512 and nhr-8(ok186) worms.
QPCR quantification of mRNA levels of MDT-15 dependent detoxification genes. Values represent fold changes±SEM in CF512 or nhr-8(ok186) worms vs. N2 worms, calculated from the average relative mRNA levels from three independent biological replicates (mRNA levels normalized to act-1). FLA = fluoranthene; NF = β-naphtoflavone.
(0.10 MB DOC)

Table S9 Expression of some toxin-induced MDT-15 targets is affected by MDT-15 depletion in adult worms.
QPCR quantification of mRNA levels of MDT-15-dependent detoxification genes in conditionally sterile CF512 worms. Values represent fold changes±SEM in *mdt-15(RNAi)* worms vs. *control(RNAi)* worms, calculated from the average relative mRNA levels from three independent biological replicates (mRNA levels normalized to act-1). FLA = fluoranthene. Genes whose induction is compromised more than two-fold in *mdt-15(RNAi)* worms are highlighted bold.
(0.08 MB DOC)

Table S10 Expression of toxin-induced MDT-15 targets is largely unaffected in nhr-49(RNAi) and sbp-1(RNAi) worms.
QPCR quantification of mRNA levels of MDT-15-dependent detoxification genes in N2 L4 stage worms fed with nhr-49 or sbp-1 RNAi. Values represent fold changes±SEM in nhr-49(RNAi) or sbp-1(RNAi) worms vs. *control(RNAi)* worms, calculated from the average relative mRNA levels from three independent biological replicates (mRNA levels normalized to act-1). FLA = fluoranthene, NF = β-napthtoflavone.
(0.11 MB DOC)

Table S11 A statistically significant overlap exists between MDT-15 dependent genes and Cd2+-dependent genes.
To determine in unbiased fashion whether MDT-15 is involved in regulation of heavy metal detoxification, we determined the overlap between the genes that are deregulated following MDT-15 depletion and genes responsive to Cd2+ (taken from Cui et al. [45]). For further details, see Materials and Methods.
(0.04 MB DOC)
