# Supplementary figures and images for: The Mediator Subunit MDT-15 Confers Metabolic Adaptation to Ingested Material
Source: PLoS Genet. 2008 Feb 29;4(2):e1000021. doi: 10.1371/journal.pgen.1000021 (PMC2265483; doi:10.1371/journal.pgen.1000021)

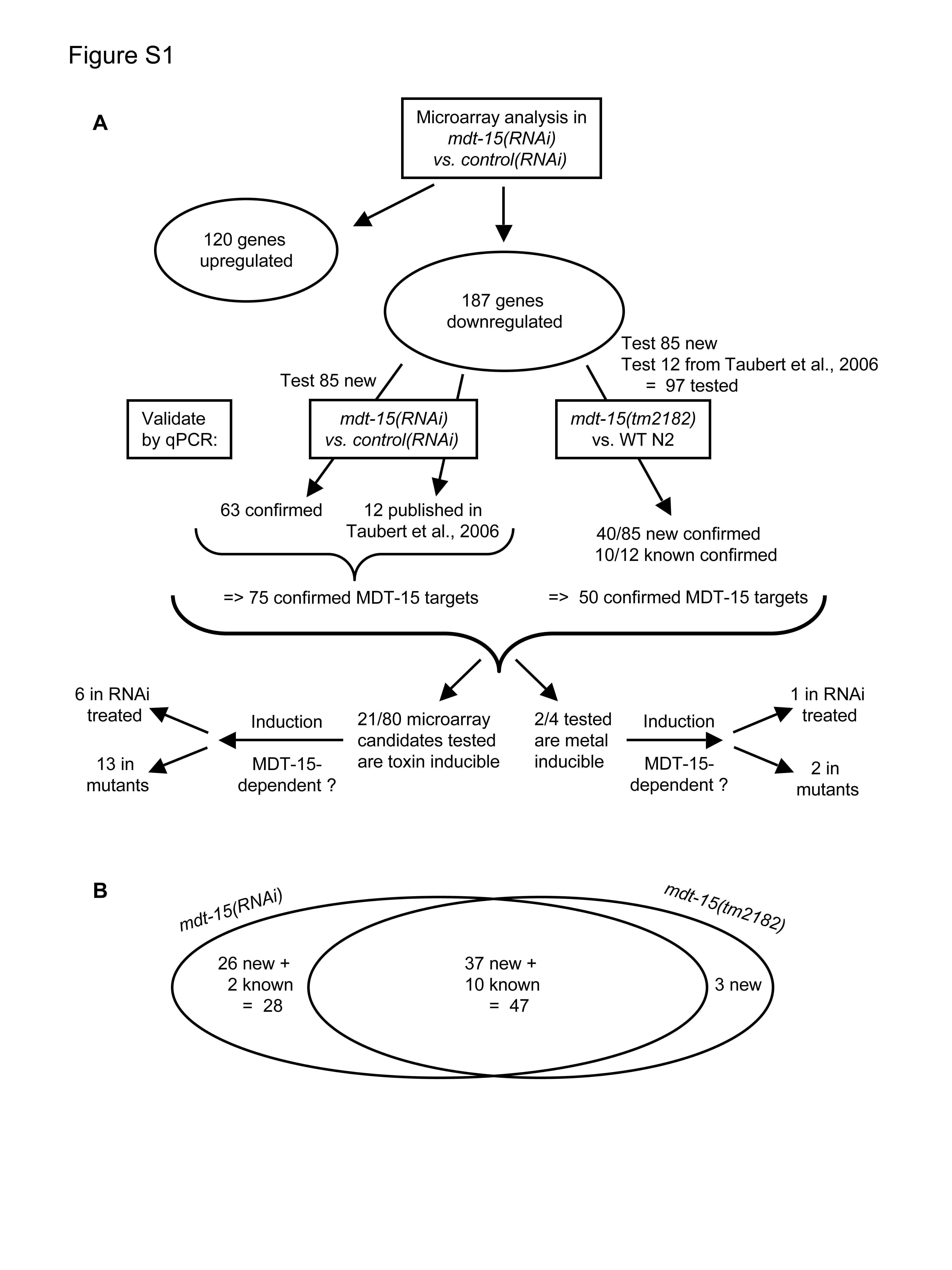

Supplement: Figure S1 — Flowchart outlining identification and confirmation of new MDT-15 targets, and overlap between genes deregulated following MDT-15 depletion vs. mutation. (A) The flowchart depicts the workflow regarding identification (by microarrays) and confirmation (by qPCR) of MDT-15 targets. Validation by qPCR was performed in both mdt-15(RNAi) worms and mdt-15(tm2182) mutants. (B) Overlap and separation of genes deregulated in mdt-15(RNAi) worms and mdt-15(tm2182) mutants. (3.99 MB TIF) [file pgen.1000021.s001.tif]

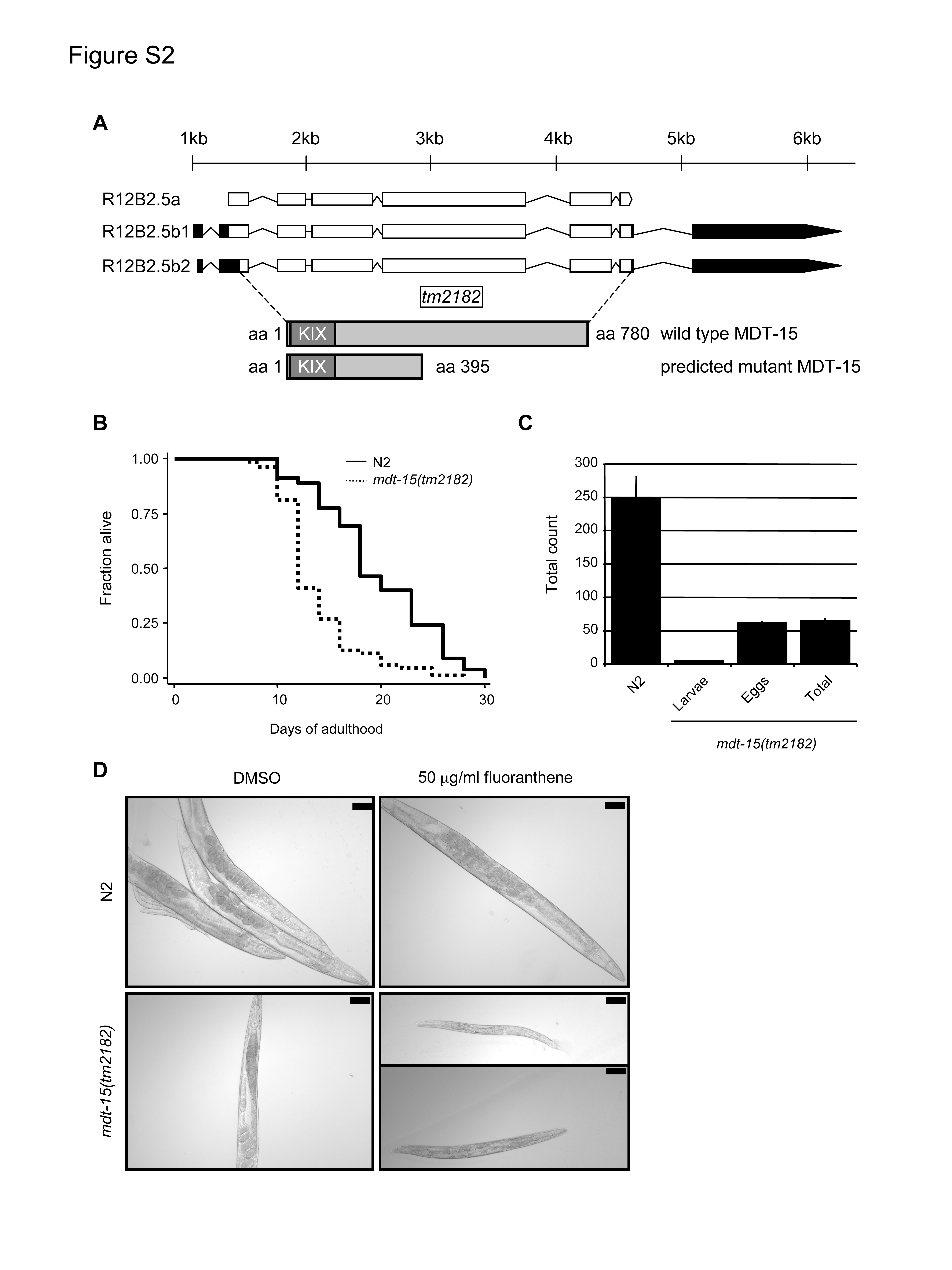

Supplement: Figure S2 — Worms carrying a mutation in the MDT-15 gene exhibit short lifespan, reduced brood size, and toxin hypersensitivity. (A) Structure of the MDT-15 gene, and its three mRNA and two protein products. For mRNAs, non-coding exons are black, coding exons are white and introns are indicated as lines. The box labeled tm2182 indicates the location of the genomic tm2182 mutation. For protein products, light grey indicates the ORF, with the dark grey box highlighting the KIX domain. The tm2182 mutation is predicted to result in the production of a truncated protein, as indicated. (B) Life span analysis of wild-type N2 and mutant mdt-15(tm2182) worms reveals short life span of the mutant worms. Mean life spans (measured in days after reaching adulthood) were 19.7 for N2 worms (filled line; n = 78), and 13.7 days for mdt-15(tm2182) mutants (dotted line; n = 76); P-value <0.0001 (log rank test). The data shown here were obtained with worms cultured at 20°C; similar results were obtained when worms were grown at 25°C (data not shown). (C) Total brood count analysis of wild-type N2 and mutant mdt-15(tm2182) worms reveals reduced brood size of the mutant worms. The mdt-15(tm2182) mutants gave rise to fewer eggs, and most eggs failed to hatch. Total brood size for mutant worms is the sum of unhatched eggs and larvae. Bar graphs represent the total number of progeny±SEM (n = 17 individual worms). (D) N2 and mdt-15(tm2182) worms were grown on plates harboring fluoranthene at 10 µg/ml. After four days, animals were scored for developmental defects. Micrographs show representative animals grown on toxin or DMSO (solvent), as indicated. The size bar represents 64.5 µm. Exposure of mdt-15(tm2182) worms to fluoranthene results in small, scrawny adults; on DMSO, mdt-15(tm2182) animals are only slightly thinner than N2 worms. (5.81 MB TIF) [file pgen.1000021.s002.tif]

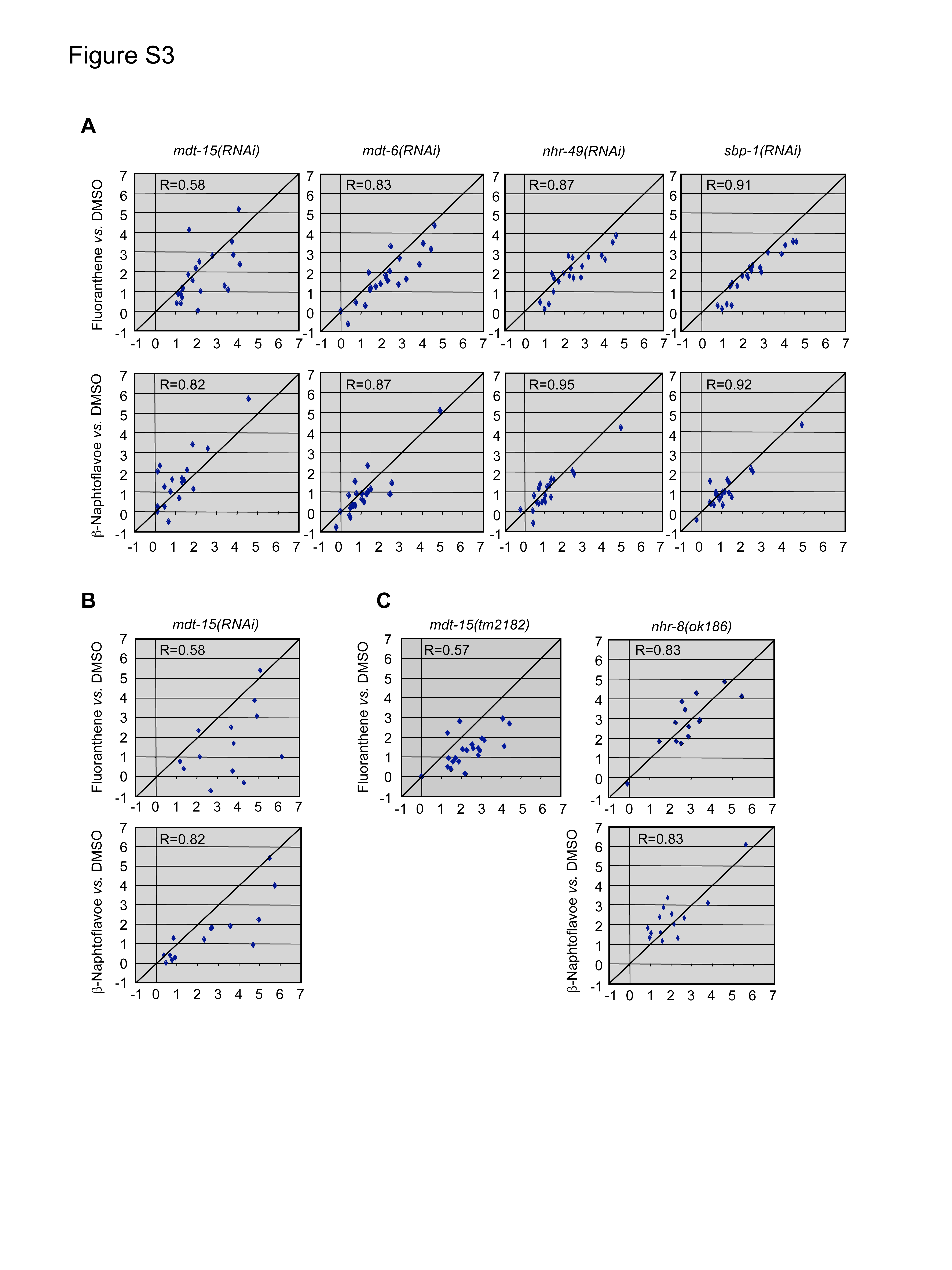

Supplement: Figure S3. — MDT-15 is required to induce select detoxification genes. The scatter plots depict pairwise comparisons of detoxification gene inductions by fluoranthene or β-naphtoflavone in different worm strains. Each data point represents the log of the fold-induction of an individual gene in control(RNAi) (A,B) or wild-type N2 worms (C) on the X-axis vs. the log of the fold-induction of the same gene in worms exposed to the indicated RNAi bacteria (A,B), or in mutant worms (C) on the Y-axis. R-values indicate the correlation coefficients between the two samples. Panel A represents the detoxification genes characterized in Tables 3, S7, and S10. Panel B represents the 15 toxin-inducible cyp genes described in Table S6. Panel C represents the detoxification genes described in Tables 4 and S8. Correlation is generally poorer between wild-type/control(RNAi) worms and worms with mutated or depleted MDT-15 (R<0.6 for fluoranthene exposure) than between wild-type/control(RNAi) worms and worms with either mutated or depleted MDT-6, NHR-49 or NHR-8 (R>0.8 for fluoranthene exposure). (4.30 MB TIF) [file pgen.1000021.s003.tif]

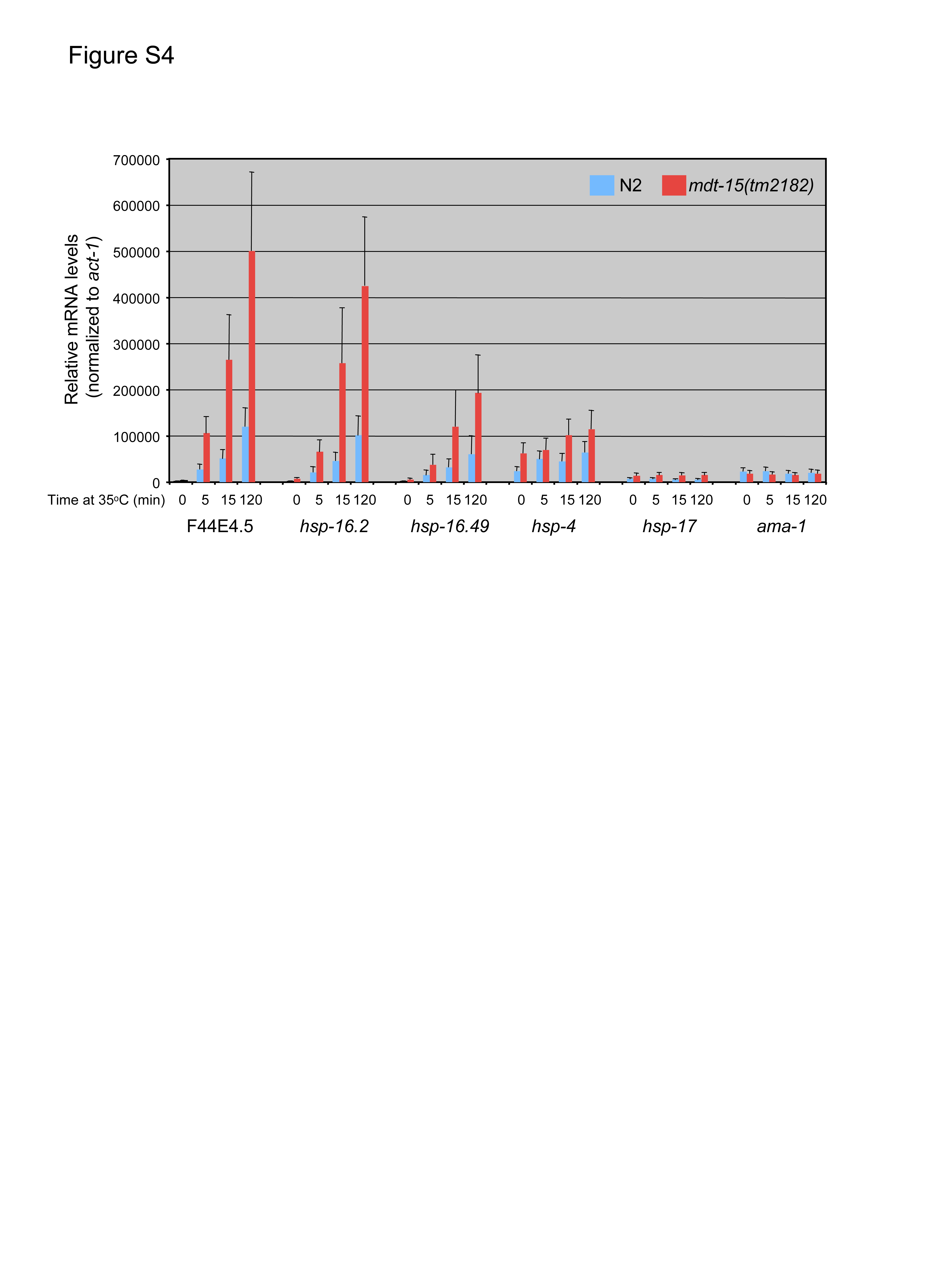

Supplement: Figure S4 — MDT-15 is dispensable for the heat-shock response. Quantification of the mRNA levels of select heat-shock protein genes in L4-stage N2 worms (blue) and mdt-15(tm2182) worms (red) exposed to 35oC for various times, as indicated. Each bar represents the average relative mRNA level of the indicated gene (average from three independent worm growths and RNA isolations from N2 L4 stage animals). Relative mRNA levels are normalized to act-1 mRNA levels; the error bars represent SEM. (2.89 MB TIF) [file pgen.1000021.s004.tif]
