# Supplementary material for: The Mediator Subunit MDT-15 Confers Metabolic Adaptation to Ingested Material
Source: PLoS Genet. 2008 Feb 29;4(2):e1000021. doi: 10.1371/journal.pgen.1000021 (PMC2265483; doi:10.1371/journal.pgen.1000021)
Supplement: Table S1 — List of genes deregulated in mdt-15(RNAi) worms (compared to control(RNAi) worms). The data represent the output of “limma” analysis from five independent mRNA isolations and microarray hybridizations. “ID” refers to the identity of individual spots on the arrays, “M” represents the log of the fold change, “A” represents the averaged spot intensity, and “Function” refers to the predicted or actual function according to WormBase (WS157). In the “Name” column, detoxification related genes are bold; previously recognized MDT-15-dependent genes are italic; HSP-like proteins are underlined. Table S1A lists genes downregulated in mdt-15(RNAi) animals, whereas Table S1B lists genes upregulated in mdt-15(RNAi) animals. Genes are ordered by highest statistical significance of over/underexpression in mdt-15(RNAi) animals vs. control(RNAi) animals. (0.59 MB DOC) [file pgen.1000021.s005.doc]

*Supporting Table S1: List of genes deregulated* in mdt-15(RNAi) *worms (compared to*

control(RNAi) *worms).*

The data represent the output of “limma” analysis from five independent mRNA isolations and microarray hybridizations. “ID” refers to the identity of individual spots on the arrays, “M” represents the log of the fold change, “A” represents the averaged spot intensity, and “Function” refers to the predicted or actual function according to WormBase (WS157). In the “Name” column, detoxification related genes are in **bold**; previously recognized MDT-15-dependent genes are *italic*; HSP-like proteins are underlined. Table S1A lists genes downregulated in *mdt-15(RNAi)* animals, whereas Table S1B lists genes upregulated in *mdt-15(RNAi)* animals. Genes are ordered by highest statistical significance of over/underexpression in *mdt-15(RNAi)* animals *vs. control(RNAi)* animals.

| **Table S1A: Genes downregulated in *mdt-15(RNAi)* animals** | | | | | | |
| --- | --- | --- | --- | --- | --- | --- |
| **ID** | **Name** | **M** | **A** | ***P*-value** | **CGC name** | **Function** |
| cea2.c.15186 | **Y38F1A.6** | 3.00 | 10.16 | 0.00 |  | Alcohol dehydrogenase, class IV |
| cea2.d.03465 | F44G3.2 | 5.39 | 8.09 | 0.00 |  | Creatine kinases |
| cea2.d.07775 | C23H5.8a | 2.82 | 9.63 | 0.00 |  | Peptidyl-prolyl cis-trans isomerase |
| cea2.d.20845 | F15E11.1 | 4.52 | 11.28 | 0.00 |  |  |
| cea2.p.114207 | F21C10.9 | 5.45 | 8.70 | 0.00 |  |  |
| cea2.c.31436 | *VZK822L.1* | *3.47* | 9.26 | 0.00 | *fat-6* | Fatty acid desaturase |
| cea2.p.123614 | T05E12.6 | 2.19 | 8.41 | 0.00 |  |  |
| cea2.c.22651 | T05G5.6 | 1.91 | 11.98 | 0.00 | *ech-6* | Enoyl-CoA hydratase |
| cea2.c.38326 | **T08G5.10** | 3.23 | 10.90 | 0.00 | *mtl-2* | Predicted metallothionein |
| cea2.p.40725 | F54D5.12 | 2.48 | 8.37 | 0.00 |  | Proteins containing the FAD binding domain |
| cea2.p.125267 | **T19H12.9** | 2.71 | 6.82 | 0.00 | *ugt-12* | UDP-glucuronosyl and UDP-glucosyl transferase |
| cea2.d.21652 | F22B8.6 | 2.64 | 9.72 | 0.00 |  | Cystathionine beta-lyases/cystathionine gamma-synthases |
| cea2.p.135278 | **C29F7.2** | 2.35 | 8.22 | 0.00 |  | Predicted small molecule kinas |
| cea2.d.01138 | **C33A12.6** | 1.53 | 8.18 | 0.00 | *ugt-21* | UDP-glucuronosyl and UDP-glucosyl transferase |
| cea2.c.38575 | **T10H9.5** | 3.11 | 9.22 | 0.00 | *pmp-5* | Long-chain acyl-CoA transporter, ABC superfamily (involved in peroxisome organization and biogenesis) |
| cea2.d.23024 | **F32D8.12** | 2.83 | 7.16 | 0.00 |  | Proteins containing the FAD binding domain |
| cea2.i.32631 | **C04F5.7** | 3.30 | 6.75 | 0.00 | *ugt-63* | UDP-glucuronosyl and UDP-glucosyl transferase |
| cea2.c.15603 | Y57A10C.6 | 1.70 | 8.50 | 0.00 |  | Peroxisomal 3-ketoacyl-CoA-thiolase P-44/SCP2 |
| cea2.p.128763 | Y45G5AM.1 | 2.26 | 9.01 | 0.00 | *nhr-114* | Hormone receptors |
| cea2.i.45011 | *T22G5.2* | 1.57 | 8.99 | 0.00 | *lbp-7* | Fatty acid-binding protein FABP |
| cea2.d.21251 | F18E3.7a | 2.12 | 7.45 | 0.00 |  | D-aspartate oxidase |
| cea2.d.21252 | F18E3.7a | 2.03 | 8.04 | 0.00 |  | D-aspartate oxidase |
| cea2.i.58987 | *T22G5.6* | 5.37 | 5.75 | 0.00 | *lbp-8* | Fatty acid-binding protein FABP |
| cea2.p.158386 | F15E11.14 | 4.07 | 9.82 | 0.00 |  |  |
| cea2.d.22041 | F25E5.8a | 1.92 | 8.93 | 0.00 |  |  |
| cea2.p.135471 | C31E10.7 | 1.56 | 12.24 | 0.00 |  | Cytochrome b5 |
| cea2.d.21651 | F22B8.6 | 2.85 | 9.46 | 0.00 |  | Cystathionine beta-lyases/cystathionine gamma-synthases |
| cea2.i.44641 | **T16G1.6** | 3.51 | 7.10 | 0.00 |  | Predicted small molecule kinase |
| cea2.c.39182 | *W06D12.3* | 3.05 | 7.02 | 0.00 | *fat-5* | Fatty acid desaturase |
| cea2.c.45124 | F46G10.6 | 2.21 | 8.97 | 0.00 | *mxl-3* | Upstream transcription factor 2/L-myc-2 protein |
| cea2.d.43447 | Y38E10A.12 | 1.96 | 6.81 | 0.00 |  |  |
| cea2.c.35201 | *F10D2.9* | 3.43 | 6.22 | 0.00 | *fat-7* | Fatty acid desaturase |
| cea2.d.49450 | ZK6.7b | 1.70 | 9.18 | 0.00 |  | Triglyceride lipase-cholesterol esterase |
| cea2.i.41485 | K09C6.9 | 2.16 | 6.55 | 0.00 |  |  |
| cea2.p.119636 | **F59B1.8** | 1.80 | 7.31 | 0.00 |  | Predicted small molecule kinase |
| cea2.d.12587 | C14A6.1 | 2.96 | 6.01 | 0.00 | *clec-48* | C-type lectin |
| cea2.p.139508 | **F09E10.3** | 1.56 | 8.72 | 0.00 | *dhs-25* | Mitochondrial/plastidial beta-ketoacyl-ACP reductase |
| cea2.p.69884 | R02F2.4 | 2.47 | 8.08 | 0.00 |  |  |
| cea2.c.31464 | *W02A2.1* | 1.20 | 10.30 | 0.00 | *fat-2* | Fatty acid desaturase |
| cea2.c.31678 | **Y37A1B.5** | 1.93 | 9.32 | 0.00 |  | Selenium-binding protein |
| cea2.d.01807 | *C55B7.4a* | 4.49 | 10.94 | 0.00 | *acdh-1* | Short-chain acyl-CoA dehydrogenase |
| cea2.c.32957 | ZK550.6 | 1.18 | 9.06 | 0.00 |  | Peroxisomal phytanoyl-CoA hydroxylase |
| cea2.i.25168 | F08G5.6 | 2.32 | 9.35 | 0.01 |  |  |
| cea2.p.112208 | F08H9.7 | 1.10 | 7.67 | 0.01 | *clec-56* | C-type lectin |
| cea2.p.39115 | **F37H8.3** | 1.78 | 6.67 | 0.01 |  | Predicted hydrolase (HAD superfamily) |
| cea2.d.34418 | R01E6.3b | 1.29 | 11.81 | 0.01 | *cah-4* | Carbonic anhydrase |
| cea2.d.26120 | F45D11.14 | 2.71 | 7.52 | 0.01 |  |  |
| cea2.d.35575 | *R12B2.5a* | 1.39 | 8.80 | 0.01 | *mdt-15* | Positive cofactor 2 (PC2), subunit of a multiprotein coactivator of RNA polymerase II |
| cea2.3.08745 | F09F7.4b | 1.44 | 8.17 | 0.01 |  | Enoyl-CoA hydratase |
| cea2.p.119757 | H12C20.3 | 2.48 | 7.24 | 0.01 | *nhr-68* | Hormone receptors |
| cea2.c.50267 | Y38E10A.13 | 2.92 | 8.90 | 0.01 |  |  |
| cea2.p.131872 | B0272.3 | 0.92 | 7.73 | 0.01 |  | 3-hydroxyacyl-CoA dehydrogenase |
| cea2.i.16017 | T28D9.3 | 1.16 | 8.20 | 0.01 |  | Lipid phosphate phosphatase and related enzymes of the PAP2 family |
| cea2.p.116259 | F37B4.7 | 1.80 | 11.44 | 0.01 |  | Micronutrient transporters (folate transporter family) |
| cea2.p.113212 | F15E11.12 | 2.97 | 8.52 | 0.01 |  |  |
| cea2.p.107923 | **C06B3.3** | 2.86 | 9.70 | 0.01 | *cyp-35C1* | Cytochrome P450 CYP2 subfamily |
| cea2.c.38324 | **T08B1.3** | 1.21 | 9.01 | 0.01 | *alh-5* | Aldehyde dehydrogenase |
| cea2.i.17130 | Y49F6C.6 | 1.40 | 8.19 | 0.01 |  |  |
| cea2.c.21904 | **M88.1** | 1.99 | 9.46 | 0.01 | *ugt-62* | UDP-glucuronosyl and UDP-glucosyl transferase |
| cea2.d.07846 | F09F7.4a | 1.35 | 12.60 | 0.01 |  | Enoyl-CoA hydratase |
| cea2.c.29283 | F58F9.7 | 1.91 | 6.81 | 0.01 |  | Pristanoyl-CoA/acyl-CoA oxidase |
| cea2.p.106691 | **AC3.7** | 2.40 | 7.81 | 0.01 | *ugt-1* | UDP-glucuronosyl and UDP-glucosyl transferase |
| cea2.i.17200 | Y51H7C.12 | 1.32 | 7.17 | 0.01 |  | Defense-related protein containing SCP domain |
| cea2.i.42290 | **R04B5.9** | 1.14 | 9.15 | 0.01 | *ugt-47* | UDP-glucuronosyl and UDP-glucosyl transferase |
| cea2.i.41998 | M02H5.8 | 0.93 | 11.99 | 0.01 |  |  |
| cea2.p.73702 | T17A3.8 | 1.40 | 7.26 | 0.01 | *ver-2* | FGF/PDGF-receptor and related receptor tyrosine kinases; Uncharacterized conserved protein, contains ZZ-type Zn-finger |
| cea2.d.43448 | Y38E10A.12 | 1.29 | 8.20 | 0.01 |  |  |
| cea2.p.126530 | W02H5.8 | 1.45 | 8.10 | 0.01 |  | Dihydroxyacetone kinase/glycerone kinase |
| cea2.p.113380 | F17A9.6 | 1.58 | 6.70 | 0.01 | *ceh-49* | CCAAT displacement protein and related homeoproteins |
| cea2.i.47201 | Y45G12C.1 | 0.89 | 8.77 | 0.01 |  |  |
| cea2.i.00408 | **C10H11.6** | 1.56 | 6.84 | 0.01 | *ugt-26* | UDP-glucuronosyl and UDP-glucosyl transferase |
| cea2.p.35503 | F07H5.9 | 1.32 | 6.10 | 0.01 |  | Lysosomal & prostatic acid phosphatases |
| cea2.d.20465 | F13G11.1a | 1.35 | 7.28 | 0.01 | *tag-193* | Transcription factor Doublesex |
| cea2.c.10738 | E04F6.3 | 1.64 | 6.93 | 0.01 | *maoc-1* | Peroxisomal multifunctional beta-oxidation protein and related enzymes |
| cea2.p.115954 | F35E12.6 | 0.84 | 8.70 | 0.01 |  |  |
| cea2.p.36156 | **F11G11.1** | 1.14 | 7.47 | 0.02 | *gst-8* | Glutathione S-transferase |
| cea2.d.10890 | C05E4.9b | 1.12 | 10.49 | 0.02 | *gei-7* | Malate synthase; Isocitrate lyase |
| cea2.i.36871 | F17C11.6 | 1.76 | 8.83 | 0.02 |  |  |
| cea2.c.10694 | E01G4.3 | 1.09 | 10.69 | 0.02 |  |  |
| cea2.d.08018 | *R12B2.5b* | 1.45 | 6.84 | 0.02 | *mdt-15* | Positive cofactor 2 (PC2), subunit of a multiprotein coactivator of RNA polymerase II |
| cea2.p.48630 | W05H5.3 | 2.07 | 7.23 | 0.02 |  | Na+/Pi symporter |
| cea2.d.19995 | **F11G11.2** | 1.04 | 10.03 | 0.02 | *gst-7* | Glutathione S-transferase |
| cea2.p.36472 | F14E5.5 | 1.54 | 8.47 | 0.02 |  | Triacylglycerol lipase |
| cea2.c.37679 | R08E5.3 | 1.71 | 6.65 | 0.02 |  | SAM-dependent methyltransferases |
| cea2.p.110139 | C29F3.7 | 1.05 | 7.13 | 0.02 |  |  |
| cea2.c.09507 | C05C10.3 | 1.60 | 9.80 | 0.02 |  | Succinyl-CoA:alpha-ketoacid-CoA transferase |
| cea2.p.38960 | **F35H8.6** | 0.84 | 8.32 | 0.02 | *ugt-58* | UDP-glucuronosyl and UDP-glucosyl transferase |
| cea2.c.44881 | F41E7.4 | 1.17 | 9.53 | 0.02 |  |  |
| cea2.c.16065 | ZK1307.1 | 1.07 | 11.36 | 0.02 |  |  |
| cea2.p.119287 | **F58B4.5** | 1.32 | 10.08 | 0.02 |  | Predicted small molecule kinase |
| cea2.3.08441 | *F08A8.2* | 2.79 | 7.36 | 0.02 |  | Acyl-CoA oxidase |
| cea2.p.126942 | W06A7.4 | 1.44 | 6.04 | 0.02 |  |  |
| cea2.i.25327 | **F15B10.1** | 1.03 | 9.60 | 0.02 |  | UDP-N-acetylglucosamine transporter [ |
| cea2.i.23035 | B0212.2 | 1.10 | 8.11 | 0.02 | *sre-5* | Serpentine Receptor, class E (epsilon) |
| cea2.d.42799 | Y19D10A.9 | 2.46 | 8.62 | 0.02 |  | C-type lectin |
| cea2.i.44546 | T15B7.12 | 2.05 | 6.89 | 0.02 |  |  |
| cea2.d.38519 | T10B5.8 | 1.36 | 6.35 | 0.02 |  | NADH:flavin oxidoreductase/12-oxophytodienoate reductase |
| cea2.d.07880 | F26B1.2b | 0.94 | 9.22 | 0.02 |  | PolyC-binding hnRNP-K protein HRB57A/hnRNP, contains KH domain |
| cea2.i.00393 | **C10H11.3** | 1.98 | 6.32 | 0.02 | *ugt-25* | UDP-glucuronosyl and UDP-glucosyl transferase |
| cea2.i.57281 | **T18D3.3** | 1.03 | 7.42 | 0.02 |  | Zn^2+^ transporter |
| cea2.3.08464 | *F08A8.3* | 1.68 | 8.08 | 0.02 |  | Acyl-CoA oxidase |
| cea2.p.28829 | **C01G6.7** | 1.02 | 8.05 | 0.02 |  | Acyl-CoA synthetase |
| cea2.p.131236 | ZK6.11 | 0.80 | 10.88 | 0.02 |  |  |
| cea2.i.38062 | F31F4.15 | 0.99 | 9.44 | 0.02 | *fbxa-72* | F-box A protein |
| cea2.3.08729 | F09F7.4b | 1.63 | 6.42 | 0.02 |  | Enoyl-CoA hydratase |
| cea2.i.59584 | **T21E8.2** | 1.69 | 5.50 | 0.02 | *pgp-7* | Multidrug/pheromone exporter, ABC superfamily |
| cea2.3.05578 | C38D9.9 | 1.77 | 6.19 | 0.02 |  | F-box & FTH/DUF38 domains |
| cea2.i.41646 | K10G4.2 | 1.55 | 7.49 | 0.02 | *srw-47* | 7-transmembrane olfactory receptor |
| cea2.p.116409 | **F39G3.1** | 1.65 | 7.23 | 0.02 | *ugt-61* | UDP-glucuronosyl and UDP-glucosyl transferase |
| cea2.i.07060 | Y71F9B.1 | 0.87 | 9.18 | 0.02 |  |  |
| cea2.i.49060 | ZK262.6 | 1.64 | 6.95 | 0.02 | *srw-83* | 7-transmembrane olfactory receptor |
| cea2.d.00807 | C23H5.8b | 1.68 | 7.73 | 0.02 |  |  |
| cea2.c.47012 | M02D8.4a | 1.16 | 7.29 | 0.02 |  | Asparagine synthase (glutamine-hydrolyzing) |
| cea2.c.15872 | ZK945.1 | 1.16 | 7.85 | 0.02 |  | Predicted esterase |
| cea2.c.11194 | **F11G11.3** | 0.71 | 9.33 | 0.02 | *gst-6* | Glutathione S-transferase |
| cea2.p.76278 | **Y39A1A.19** | 0.78 | 7.23 | 0.02 | *fmo-3* | Flavin-containing monooxygenase |
| cea2.d.33151 | K09H11.7 | 1.36 | 8.92 | 0.02 |  | p-Nitrophenyl phosphatase |
| cea2.p.115910 | **F35B12.2** | 2.49 | 5.84 | 0.02 | *dhs-20* | Short-chain dehydrogenase predicted to be mitochondrial |
| cea2.p.37875 | F28A10.6 | 0.87 | 8.52 | 0.02 |  | Medium-chain acyl-CoA dehydrogenase |
| cea2.d.02807 | F26A1.8 | 0.99 | 7.81 | 0.02 |  |  |
| cea2.3.08483 | *F08A8.4* | 1.55 | 9.09 | 0.02 |  | Acyl-CoA oxidase |
| cea2.c.05364 | R11A5.4 | 0.67 | 11.83 | 0.02 |  | Phosphoenolpyruvate carboxykinase |
| cea2.p.113511 | F18E2.1 | 0.66 | 8.82 | 0.02 |  | Purple acid phosphatase |
| cea2.d.21967 | **F25D1.5** | 1.99 | 8.48 | 0.02 |  | Reductases with broad range of substrate specificities |
| cea2.3.04489 | C29F9.3b | 1.58 | 7.13 | 0.02 |  |  |
| cea2.d.38988 | **T16G1.7** | 1.25 | 8.20 | 0.02 |  | Predicted small molecule kinase |
| cea2.i.11050 | F12E12.5 | 0.78 | 9.90 | 0.02 | *sdz-12* | SKN-1 Dependent Zygotic transcript, Zn-Finger |
| cea2.d.13978 | C24B9.13 | 0.92 | 8.38 | 0.02 | *srx-25* | Serpentine Receptor, class X |
| cea2.d.07740 | *C05E4.9a* | 1.07 | 11.77 | 0.02 | *gei-7* | Malate synthase; Isocitrate lyase |
| cea2.c.34112 | C24B9.3 | 0.74 | 10.36 | 0.02 |  |  |
| cea2.i.05368 | W06D4.5 | 1.19 | 6.87 | 0.03 | *tag-157* | Sorting nexin SNX11 |
| cea2.p.108173 | **C08B6.1** | 2.93 | 6.69 | 0.03 | *ugt-17* | UDP-glucuronosyl and UDP-glucosyl transferase |
| cea2.i.39132 | F44E7.8 | 1.33 | 7.02 | 0.03 | *nhr-142* | Hormone receptors |
| cea2.c.26820 | C39E9.8 | 1.03 | 9.02 | 0.03 |  |  |
| cea2.c.16090 | ZK1320.9 | 0.77 | 7.23 | 0.03 |  | Acetyl-CoA hydrolase |
| cea2.i.53060 | F21E9.3 | 1.58 | 5.93 | 0.03 |  | Uncharacterized protein with conserved cysteine |
| cea2.d.36038 | R151.2a | 0.91 | 9.20 | 0.03 |  | Ribose-phosphate pyrophosphokinase |
| cea2.i.40699 | **H23N18.1** | 2.11 | 6.72 | 0.03 | *ugt-13* | UDP-glucuronosyl and UDP-glucosyl transferase |
| cea2.d.21244 | F18E3.7a | 1.63 | 5.97 | 0.03 |  | D-aspartate oxidase |
| cea2.i.34701 | **C49G7.8** | 1.41 | 8.19 | 0.03 | *cyp-35A4* | Cytochrome P450 CYP2 subfamily |
| cea2.p.43984 | **R03D7.6** | 0.86 | 11.06 | 0.03 | *gst-5* | Glutathione S-transferase [ |
| cea2.3.30865 | ZC455.6a | 1.48 | 6.37 | 0.03 | *ugt-5* | UDP-glucuronosyl and UDP-glucosyl transferase |
| cea2.c.03324 | F36A2.3 | 1.30 | 8.32 | 0.03 |  | Predicted malate dehydrogenase |
| cea2.p.120263 | **K01D12.12** | 0.87 | 7.27 | 0.03 | *cdr-6* | Failed axon connections (fax) protein/glutathione S-transferase-like protein |
| cea2.c.18644 | F09G8.6 | 1.14 | 7.70 | 0.04 | *col-91* | Collagens (type IV and type XIII), and related proteins |
| cea2.p.131138 | **ZC455.4** | 1.66 | 7.29 | 0.03 | *ugt-6* | UDP-glucuronosyl and UDP-glucosyl transferase |
| cea2.i.18499 | C02C2.4 | 0.91 | 7.96 | 0.03 |  | Permease of the major facilitator superfamily |
| cea2.i.25360 | F17E9.11 | 1.49 | 6.05 | 0.04 | *lys-10* | N-acetylmuraminidase/lysozyme |
| cea2.3.01831 | C06A8.1b | 0.84 | 9.27 | 0.03 |  | 5,10-methylenetetrahydrofolate reductase |
| cea2.c.18783 | F23F12.12 | 1.22 | 9.14 | 0.04 |  |  |
| cea2.p.40038 | F46C5.10 | 0.98 | 9.00 | 0.03 |  |  |
| cea2.3.08458 | *F08A8.3* | 1.17 | 8.79 | 0.04 |  | Acyl-CoA oxidase |
| cea2.d.19994 | **F11G11.2** | 1.10 | 9.22 | 0.03 | *gst-7* | Glutathione S-transferase |
| cea2.i.23471 | C09G12.3 | 0.86 | 9.42 | 0.04 | *srz-78* | 7-transmembrane receptor |
| cea2.i.51625 | C43H6.8 | 1.12 | 7.30 | 0.04 | *hlh-15* | Transcription factor HAND2/Transcription factor TAL1/TAL2/LYL1 |
| cea2.3.07413 | D1009.1b | 0.99 | 8.17 | 0.04 |  | Very long-chain acyl-CoA synthetase/fatty acid transporter |
| cea2.d.29105 | F56B3.11b | 0.78 | 7.54 | 0.04 |  | Uncharacterized conserved protein |
| cea2.i.57763 | T26C11.5 | 1.13 | 8.11 | 0.04 | *ceh-41* | ONECUT class CUT homeobox protein |
| cea2.i.41530 | **K09D9.2** | 1.76 | 7.59 | 0.04 | *cyp-35A3* | Cytochrome P450 CYP2 subfamily |
| cea2.i.39288 | F47B8.2 | 0.92 | 7.87 | 0.04 |  |  |
| cea2.i.45763 | W04E12.1 | 1.07 | 8.12 | 0.04 | *fbxa-131* | F-box A protein |
| cea2.c.45102 | F46F2.3 | 0.96 | 11.99 | 0.04 |  |  |
| cea2.i.40709 | **H23N18.3** | 0.76 | 8.13 | 0.04 | *ugt-8* | UDP-glucuronosyl and UDP-glucosyl transferase |
| cea2.i.16003 | T27A1.5 | 0.81 | 8.32 | 0.04 |  | Amino acid transporters |
| cea2.d.05237 | T04C9.1c | 0.67 | 9.50 | 0.04 |  | Oligophrenin-1 and related Rho GTPase-activating proteins |
| cea2.p.16080 | **T02E1.5** | 0.81 | 9.40 | 0.04 | *dhs-3* | Hydroxysteroid 17-beta dehydrogenase 11 |
| cea2.d.22788 | F31D5.3c | 0.82 | 8.51 | 0.04 | *tag-149* | Copine |
| cea2.d.46470 | Y69H2.3d | 0.69 | 8.29 | 0.04 |  | Fibrillins and related proteins containing Ca2+-binding EGF-like domains Uncharacterized protein |
| cea2.c.26589 | C29E6.5 | 1.29 | 7.55 | 0.04 | *nhr-43* | Hormone receptors |
| cea2.d.49436 | ZK6.7a | 1.32 | 11.02 | 0.04 |  | Triglyceride lipase-cholesterol esterase |
| cea2.i.00012 | B0041.6 | 0.65 | 8.31 | 0.04 | *ptps-1* | 6-pyruvoyl tetrahydrobiopterin synthase |
| cea2.p.119579 | F59A1.10 | 0.74 | 8.97 | 0.04 |  | Acyl-CoA:diacylglycerol acyltransferase (DGAT) |
| cea2.p.93077 | **F49C12.7** | 1.63 | 8.05 | 0.04 |  | Predicted small molecule kinase |
| cea2.d.34276 | M162.4 | 1.43 | 7.44 | 0.04 | *srt-46* | Serpentine Receptor, class T |
| cea2.i.37933 | F28H7.4 | 0.68 | 7.36 | 0.04 |  | Selenoprotein T |
| cea2.d.07855 | F15A4.8b | 1.85 | 7.07 | 0.04 |  | Chitinase |
| cea2.d.13146 | *C17C3.12b* | 1.21 | 9.13 | 0.04 | *acdh-2* | Short-chain acyl-CoA dehydrogenase |
| cea2.d.00934 | C27A2.2b | 0.80 | 9.57 | 0.04 | *rpl-22* | 60S ribosomal protein L22 |
| cea2.i.61248 | F01D5.2 | 1.43 | 7.30 | 0.04 |  | Secreted surface protein |
| cea2.p.13796 | K04F10.6 | 0.71 | 8.68 | 0.04 | *rde-3* | S-M checkpoint control protein CID1 and related nucleotidyltransferases |
| cea2.d.30166 | F58B3.1 | 1.25 | 10.99 | 0.04 | *lys-4* | N-acetylmuraminidase/lysozyme |
| cea2.c.16776 | C05D11.5 | 1.02 | 7.28 | 0.05 |  | Hydroxypyruvate isomerase |
| cea2.p.85801 | C09B9.3 | 0.91 | 7.69 | 0.05 |  | Bestrophin (Best vitelliform macular dystrophy-associated protein) |
| cea2.d.49447 | ZK6.7b | 1.26 | 10.16 | 0.04 |  | Triglyceride lipase-cholesterol esterase |
| cea2.i.28990 | Y105C5B.10 | 1.20 | 7.86 | 0.04 | *srv-16* | Serpentine Receptor, class V |
| cea2.d.08292 | 6R55.1b | 0.60 | 7.77 | 0.04 |  | Beta-2-glycoprotein I |
| cea2.p.115587 | F31F7.1 | 0.68 | 8.06 | 0.04 |  |  |
| cea2.c.04632 | K02B12.1 | 0.61 | 8.73 | 0.04 | *ceh-6* | Transcription factor OCT-1, contains POU and HOX domains |
| cea2.p.48040 | **T26C5.1** | 0.86 | 11.02 | 0.04 | *gst-13* | Glutathione S-transferase |
| cea2.p.61872 | F17C8.2 | 0.64 | 9.76 | 0.05 | *col-89* | Collagens (type IV and type XIII), and related proteins |
| cea2.d.02481 | F16H6.3 | 0.88 | 8.93 | 0.05 |  | Secreted surface protein |
| cea2.p.158213 | F23A7.4 | 0.62 | 13.75 | 0.05 |  |  |
| cea2.p.127610 | Y32F6B.1 | 1.17 | 6.94 | 0.05 |  | Organic anion transporter |
| cea2.3.22662 | T04C9.1c | 0.70 | 8.89 | 0.05 |  | Oligophrenin-1 and related Rho GTPase-activating proteins |
| cea2.c.23575 | W09D10.3 | 0.55 | 10.89 | 0.05 |  | Mitochondrial/chloroplast ribosomal protein L12 |
| cea2.p.126273 | **W01A11.1** | 0.83 | 8.91 | 0.05 |  | Predicted hydrolases or acyltransferases (alpha/beta hydrolase superfamily) |
| cea2.c.35152 | F08F3.3 | 0.73 | 11.44 | 0.05 | *rhr-1* | Ammonium transporter RHBG |
| cea2.i.47466 | **Y50D4C.2** | 0.83 | 7.44 | 0.05 |  | Alcohol dehydrogenase, class III; Leucine-rich acidic nuclear protein |
| cea2.i.26744 | F58G6.2 | 1.01 | 10.68 | 0.05 | *srm-3* | Serpentine Receptor, class M |
| cea2.i.20314 | R02F2.9 | 0.62 | 8.01 | 0.05 |  | Predicted peptidyl-tRNA hydrolase |
| cea2.c.47062 | **M03A8.1** | 0.66 | 11.29 | 0.05 | *dhs-28* | 17-beta-hydroxysteroid dehydrogenase |

| **Table S1B: Genes upregulated in *mdt-15(RNAi)* animals** | | | | | | |
| --- | --- | --- | --- | --- | --- | --- |
| **ID** | **Name** | **M** | **A** | ***P*-Value** | **CGC name** | **Function** |
| cea2.p.86443 | C17H12.8 | -1.58 | 11.52 | 0.00 |  |  |
| cea2.p.117536 | F52E1.5 | -1.52 | 11.92 | 0.00 |  |  |
| cea2.d.06884 | Y51A2D.13a | -1.59 | 9.25 | 0.00 |  | Predicted phospholipase D |
| cea2.p.108682 | C14C6.5 | -1.68 | 10.49 | 0.00 |  | Secreted surface protein |
| cea2.p.138493 | D1005.1 | -1.32 | 11.05 | 0.00 |  | ATP-citrate lyase |
| cea2.p.78186 | Y49E10.18 | -1.20 | 10.20 | 0.00 |  | Predicted lipase |
| cea2.d.04017 | F55G11.5 | -1.73 | 8.12 | 0.00 | *dod-22* |  |
| cea2.p.115359 | F28H7.3 | -1.14 | 11.51 | 0.00 |  | Predicted lipase |
| cea2.c.34782 | C52E4.1 | -1.40 | 12.97 | 0.00 | *cpr-1* | Cysteine proteinase Cathepsin L |
| cea2.c.33918 | C14C6.2 | -1.09 | 10.50 | 0.00 |  | Secreted surface protein |
| cea2.c.42945 | F08C6.4 | -1.93 | 8.70 | 0.00 | *sto-1* | Prohibitins and stomatins of the PID superfamily |
| cea2.p.05075 | C47B2.6 | -1.51 | 10.89 | 0.00 |  | UDP-glucose 4-epimerase/UDP-sulfoquinovose synthase |
| cea2.p.114229 | F21F8.4 | -1.97 | 7.68 | 0.00 |  | Aspartyl protease |
| cea2.i.59402 | F45E4.1 | -2.40 | 7.52 | 0.01 | *arf-1.1* | GTP-binding ADP-ribosylation factor Arf1 |
| cea2.c.36464 | F52E1.7 | -1.39 | 10.81 | 0.01 | *hsp-17* | Alpha Crystallins |
| cea2.p.41971 | H41C03.1 | -0.96 | 7.86 | 0.01 |  | Phosphatidylinositol transfer protein SEC14 and related proteins |
| cea2.3.29112 | Y51A2D.13a | -1.35 | 10.50 | 0.01 |  | Predicted phospholipase D |
| cea2.i.25609 | F27C8.4 | -1.04 | 10.95 | 0.01 | *spp-18* | SaPosin-like Protein family |
| cea2.d.44779 | Y51A2D.13b | -1.45 | 7.86 | 0.01 |  | Predicted phospholipase D |
| cea2.d.08013 | R102.5a | -1.20 | 9.73 | 0.01 |  |  |
| cea2.i.32048 | B0348.2 | -2.67 | 6.99 | 0.01 |  |  |
| cea2.c.32682 | Y105C5B.28 | -1.42 | 12.81 | 0.01 | *gln-3* | Glutamine synthetase |
| cea2.p.151720 | R08E3.1 | -0.79 | 10.11 | 0.01 |  |  |
| cea2.i.20401 | R10E4.6 | -1.00 | 7.17 | 0.01 |  |  |
| cea2.c.15361 | **Y48E1B.10** | -1.18 | 8.95 | 0.01 | *gst-20* | Glutathione S-transferase |
| cea2.c.35316 | F21F8.7 | -0.79 | 13.32 | 0.01 | *asp-6* | Aspartyl protease |
| cea2.c.33402 | B0365.6 | -1.42 | 8.53 | 0.01 | *clec-41* | C-type lectin |
| cea2.i.56970 | T08A9.8 | -0.88 | 10.05 | 0.01 | *spp-4* | Prosaposin |
| cea2.p.150690 | M60.4 | -0.91 | 10.21 | 0.01 |  |  |
| cea2.i.24514 | C46A5.1 | -0.99 | 7.46 | 0.01 |  | Protein tyrosine phosphatase |
| cea2.d.35044 | R102.5b | -0.95 | 10.02 | 0.01 |  |  |
| cea2.c.47627 | T08A9.12 | -0.82 | 10.95 | 0.02 | *spp-2* | orthologous to the human gene INTERFERON GAMMA RECEPTOR 2 |
| cea2.p.103660 | Y67D8C.10b | -0.79 | 8.02 | 0.02 | *mca-3* | Calcium transporting ATPase |
| cea2.i.22810 | ZK1058.6 | -1.14 | 7.96 | 0.02 | *nit-1* | Carbon-nitrogen hydrolase |
| cea2.p.87235 | C32H11.12 | -1.87 | 8.42 | 0.02 | *dod-24* |  |
| cea2.p.117493 | F47H4.10 | -1.71 | 5.60 | 0.02 | *skr-5* | SCF ubiquitin ligase, Skp1 component |
| cea2.i.22830 | ZK112.5 | -1.12 | 6.08 | 0.02 |  |  |
| cea2.c.40645 | B0403.4 | -0.85 | 12.74 | 0.02 | *tag-320* | Thioredoxin/protein disulfide isomerase |
| cea2.i.55167 | H02F09.3 | -0.84 | 7.58 | 0.02 |  |  |
| cea2.p.156984 | ZC373.1 | -1.39 | 9.51 | 0.02 |  | Cystathionine beta-synthase and related enzymes |
| cea2.p.117793 | F53C11.1 | -1.11 | 9.24 | 0.02 |  |  |
| cea2.d.07844 | F09A5.4d | -1.34 | 7.06 | 0.02 |  | Cell cycle-associated protein Mob1-1 |
| cea2.p.95101 | K02D7.1 | -0.78 | 10.64 | 0.02 |  | Purine nucleoside phosphorylase |
| cea2.d.21719 | F22E5.6 | -3.64 | 6.47 | 0.02 |  | Polymerase delta-interacting protein PDIP1 and related proteins, contain BTB/POZ domain |
| cea2.i.39003 | F41F3.7 | -1.43 | 5.77 | 0.02 | *srx-73* | Serpentine Receptor, class X |
| cea2.p.116734 | F43H9.4 | -1.23 | 8.01 | 0.02 |  |  |
| cea2.p.95138 | K02D7.4 | -0.93 | 9.09 | 0.02 | *dsc-4* | Microsomal triglyceride transfer protein |
| cea2.d.07729 | C01B10.6a | -0.70 | 10.10 | 0.02 |  |  |
| cea2.p.111863 | D1086.3 | -1.06 | 9.61 | 0.02 |  |  |
| cea2.i.58859 | F28C12.6 | -1.24 | 6.42 | 0.03 |  | Transposase-encoded protein linked to peptidase |
| cea2.d.06296 | W04D2.1b | -1.47 | 6.48 | 0.03 | *atn-1* | Ca2+-binding actin-bundling protein (actinin), alpha chain (EF-Hand protein superfamily) |
| cea2.d.03397 | F42G2.4 | -1.12 | 7.65 | 0.02 |  | F-box & FTH/DUF38 |
| cea2.p.107596 | C02A12.4 | -1.13 | 9.31 | 0.02 | *lys-7* | N-acetylmuraminidase/lysozyme |
| cea2.c.06822 | W06D4.1 | -0.87 | 10.53 | 0.02 | *hgo-1* | Homogentisate 1,2-dioxygenase |
| cea2.d.08580 | B0228.4b | -0.92 | 6.80 | 0.02 | *tag-308* | Copine |
| cea2.p.88718 | C49C3.9 | -1.10 | 9.04 | 0.02 |  |  |
| cea2.c.37691 | *R09B5.6* | -0.84 | 9.85 | 0.02 | *hacd-1* | 3-hydroxyacyl-CoA dehydrogenase |
| cea2.d.26119 | F44G3.6 | -0.72 | 11.89 | 0.02 | *skr-3* | SCF ubiquitin ligase, Skp1 component |
| cea2.c.22395 | **R107.7** | -1.20 | 10.27 | 0.02 | *gst-1* | Glutathione S-transferase |
| cea2.d.28849 | F55C9.6 | -1.77 | 7.16 | 0.02 |  |  |
| cea2.i.36235 | F10G2.3 | -1.93 | 6.33 | 0.03 | *clec-7* | C-type lectin |
| cea2.p.82439 | ZK520.2 | -1.08 | 7.98 | 0.02 |  |  |
| cea2.i.30268 | Y45F10D.6 | -1.04 | 6.98 | 0.02 |  |  |
| cea2.d.41084 | W05G11.6a | -0.74 | 11.57 | 0.02 |  | Phosphoenolpyruvate carboxykinase |
| cea2.c.12310 | F43G6.8 | -1.82 | 6.56 | 0.03 |  | Predicted E3 ubiquitin ligase |
| cea2.d.45990 | Y60A3A.18 | -0.73 | 10.75 | 0.03 | *skr-4* | SCF ubiquitin ligase, Skp1 component |
| cea2.c.25497 | ZK637.13 | -0.80 | 8.74 | 0.03 |  |  |
| cea2.c.47097 | M60.2 | -0.63 | 11.65 | 0.03 |  | Placental protein 11 |
| cea2.i.02676 | F46A9.2 | -0.96 | 8.04 | 0.03 |  |  |
| cea2.i.08550 | C03H5.1 | -0.89 | 7.64 | 0.03 | *clec-10* | C-type lectin |
| cea2.p.158066 | C28A5.6 | -1.50 | 5.83 | 0.03 |  | Casein kinase (serine/threonine/tyrosine protein kinase); Unnamed protein |
| cea2.c.18983 | F28F5.6 | -1.00 | 8.43 | 0.03 |  |  |
| cea2.d.44273 | Y46H3A.3 | -3.16 | 9.65 | 0.03 | *hsp-16.2* | Alpha Crystallins |
| cea2.c.46743 | K06G5.1 | -0.64 | 11.40 | 0.03 |  |  |
| cea2.c.28617 | F38A5.3 | -1.15 | 9.63 | 0.03 | *lec-11* | Galectin, galactose-binding lectin |
| cea2.d.20817 | F14H3.12 | -0.77 | 7.17 | 0.03 |  | Serine/threonine protein kinase |
| cea2.c.41502 | C16B8.3 | -0.72 | 12.00 | 0.03 |  |  |
| cea2.i.23625 | C17H12.6 | -1.27 | 7.95 | 0.03 |  |  |
| cea2.d.03668 | F49E10.4b | -1.03 | 6.66 | 0.03 |  |  |
| cea2.p.121971 | R04B5.5 | -0.99 | 7.08 | 0.03 |  | Sorbitol dehydrogenase |
| cea2.d.24755 | F40F12.1 | -0.76 | 7.72 | 0.03 | *sdz-17* | Uncharacterized protein with conserved cysteine |
| cea2.p.159335 | F22A3.6 | -0.71 | 12.60 | 0.03 |  |  |
| cea2.i.48833 | ZK1037.7 | -1.27 | 6.35 | 0.04 | *srw-70* | 7-transmembrane olfactory receptor |
| cea2.d.04663 | K12H4.7a | -0.69 | 12.01 | 0.03 |  | Hydrolytic enzymes of the alpha/beta hydrolase fold |
| cea2.d.01404 | C45B2.2 | -0.78 | 11.72 | 0.03 |  |  |
| cea2.3.15332 | F49H12.6a | -0.75 | 8.07 | 0.04 | *acl-4* | Predicted phosphate acyltransferase, contains PlsC domain |
| cea2.d.49215 | ZK488.7 | -0.76 | 6.89 | 0.04 | *pqn-98* | Predicted alpha-helical protein |
| cea2.i.48857 | ZK105.1 | -1.14 | 7.72 | 0.04 |  | Predicted secreted cysteine rich protein found only in *C.elegans* |
| cea2.p.144481 | F42D1.2 | -0.69 | 11.68 | 0.04 |  | Tyrosine aminotransferase |
| cea2.p.65566 | F54C8.7 | -0.88 | 9.75 | 0.04 |  | Arfaptin and related proteins |
| cea2.p.145396 | F47A4.5 | -1.35 | 7.66 | 0.04 |  | Ca^2+^-independent phospholipase A2 |
| cea2.p.105780 | ZK550.2 | -0.64 | 7.34 | 0.04 |  | Predicted transporter/transmembrane protein |
| cea2.d.07989 | K07B1.5a | -0.74 | 10.19 | 0.04 | *acl-14* | Lysophosphatidic acid acyltransferase LPAAT and related acyltransferases |
| cea2.p.130816 | ZC250.1 | -0.70 | 8.52 | 0.04 | *cyn-17* | Cyclophilin type peptidyl-prolyl cis-trans isomerase |
| cea2.i.20505 | R10F2.2 | -0.97 | 6.56 | 0.04 | *cdh-1* | Cadherin repeats |
| cea2.c.30804 | T05E11.3 | -0.71 | 13.53 | 0.04 |  | Endoplasmic reticulum glucose-regulated protein (GRP94/endoplasmin), HSP90 family |
| cea2.d.08055 | T21D12.9a | -0.64 | 8.06 | 0.04 |  | Membrane glycoprotein LIG-1; Bestrophin (Best vitelliform macular dystrophy-associated protein) |
| cea2.i.59597 | T25C12.2 | -0.77 | 8.40 | 0.04 | *spp-9* | SaPosin-like Protein family |
| cea2.p.158278 | K08D8.5 | -1.01 | 6.94 | 0.05 |  |  |
| cea2.c.16089 | ZK1320.3 | -0.75 | 10.84 | 0.04 |  |  |
| cea2.p.120622 | K07C5.3 | -0.75 | 6.80 | 0.04 |  | ATP-dependent DNA ligase III |
| cea2.c.22331 | R74.3 | -0.70 | 10.20 | 0.04 | *xbp-1* | Transcription factor XBP-1 |
| cea2.d.33588 | K12H4.7b | -0.64 | 12.98 | 0.04 |  | Hydrolytic enzymes of the alpha/beta hydrolase fold |
| cea2.c.42782 | E02H4.1 | -0.79 | 8.26 | 0.04 | *del-1* | Non voltage-gated ion channels (DEG/ENaC family) |
| cea2.c.05418 | R12E2.2 | -0.84 | 8.61 | 0.04 |  | Uncharacterized conserved protein |
| cea2.p.99957 | W03D2.6 | -1.48 | 6.63 | 0.05 |  | C-type lectin |
| cea2.i.31736 | ZK617.2 | -1.51 | 8.27 | 0.05 |  | Triacylglycerol lipase |
| cea2.d.47680 | ZC376.8 | -0.80 | 8.04 | 0.04 |  |  |
| cea2.p.67629 | K02D10.5 | -0.59 | 9.53 | 0.04 |  | SNAP-25 (synaptosome-associated protein) component of SNARE complex |
| cea2.p.158093 | F48G7.5 | -0.76 | 9.60 | 0.04 |  | Secreted surface protein |
| cea2.c.02256 | F15C11.2a | -0.57 | 12.48 | 0.04 |  | Ubiquitin-like protein |
| cea2.3.26562 | W07G4.3 | -0.57 | 8.15 | 0.04 |  | Protein kinase |
| cea2.c.24419 | Y55B1AR.1 | -0.65 | 13.57 | 0.04 | *lec-6* | Galectin, galactose-binding lectin |
| cea2.i.31755 | ZK795.1 | -0.73 | 6.97 | 0.05 |  | Inositol polyphosphate multikinase, component of the ARGR transcription regulatory complex |
| cea2.i.21804 | Y41C4A.11 | -0.68 | 14.54 | 0.04 |  | Vesicle coat complex COPI, beta' subunit |
| cea2.c.32016 | Y54G2A.18 | -0.83 | 11.31 | 0.04 |  | B-cell receptor-associated protein and related proteins |
| cea2.p.46273 | T06D8.9 | -0.89 | 8.61 | 0.05 |  |  |
| cea2.p.83773 | B0001.1 | -0.72 | 10.26 | 0.05 |  |  |
| cea2.p.77026 | Y42G9A.4 | -0.59 | 11.96 | 0.05 |  | Mevalonate kinase MVK/ERG12 |
| cea2.c.30511 | M18.2 | -0.64 | 8.41 | 0.05 | *dlc-2* | Dynein light chain type 1 |
| cea2.p.30671 | C17G10.5 | -0.62 | 12.71 | 0.05 | *lys-8* | N-acetylmuraminidase/lysozyme |
| cea2.p.143602 | F38E9.1 | -0.65 | 8.29 | 0.05 |  | Glycosylphosphatidylinositol-specific phospholipase C |
| cea2.c.11251 | F13D12.6 | -0.62 | 12.15 | 0.05 |  | Serine carboxypeptidases (lysosomal cathepsin A) |
| cea2.i.37325 | **F21H7.1** | -1.35 | 7.01 | 0.05 | *gst-22* | Glutathione S-transferase |
