# Supplementary material for: The Mediator Subunit MDT-15 Confers Metabolic Adaptation to Ingested Material
Source: PLoS Genet. 2008 Feb 29;4(2):e1000021. doi: 10.1371/journal.pgen.1000021 (PMC2265483; doi:10.1371/journal.pgen.1000021)
Supplement: Table S2 — Validation of candidate MDT-15 targets. QPCR quantification of mRNA levels of 85 candidate MDT-15-dependent genes. Values represent fold changes±SEM in mdt-15(RNAi) worms vs. control(RNAi) worms, calculated from the average relative mRNA levels from three independent biological replicates (mRNA levels normalized to act-1). Genes whose MDT-15 dependence (as judged from the microarray experiments) was confirmed by this qPCR analysis are in bold (a gene was classified as MDT-15-dependent if its mRNA level was reduced at least two-fold at any of the three time points). The control genes ama-1 and nhr-23 are not MDT-15 dependent. Note that genes are classified into groups with similar biological functions (left column). (0.20 MB DOC) [file pgen.1000021.s006.doc]

*Supporting Table S2: Validation of candidate MDT-15 targets.*

QPCR quantification of mRNA levels of 85 candidate MDT-15-dependent genes. Values represent fold changes ± SEM in *mdt-15(RNAi)* worms *vs.* *control(RNAi)* worms, calculated from the average relative mRNA levels from three independent biological replicates (mRNA levels normalized to *act-1*). Genes whose MDT-15 dependence (as judged from the microarray experiments) was confirmed by this qPCR analysis are in **bold** (a gene was classified as MDT-15-dependent if its mRNA level was reduced at least two-fold at any of the three time points). The control genes *ama-1* and *nhr-23* are not MDT-15 dependent. Note that genes are classified into groups with similar biological functions (left column).

| **Gene class** | **Gene function** | **Gene name** | **RNAi treatment**  **(time)** | | |
| --- | --- | --- | --- | --- | --- |
|  |  |  | ***mdt-15***  **(12 hr)** | ***mdt-15***  **(24 hr)** | ***mdt-15***  **(36 hr)** |
| **DETOXIFICATION** | ABC-transporter | ***pgp-7*** | 1.39±0.66 | 0.24±0.05 | 0.34±0.09 |
|  | ABC-transporter | ***pmp-5*** | 0.68±0.03 | 0.44±0.09 | 0.21±0 |
|  | Aldehyde dehydrogenase | **Y38F1A.6** | 0.38±0.04 | 0.16±0.04 | 0.11±0.02 |
|  | Aldehyde dehydrogenase | *alh-5* | 1.24±0.27 | 0.74±0.24 | 1±0.7 |
|  | CYP450 | ***cyp-35C1*** | 0.99±0.22 | 0.36±0.05 | 0.15±0.03 |
|  | DHS | *dhs-3* | 0.8±0.11 | 0.71±0.08 | 0.62±0.08 |
|  | DHS | ***dhs-25*** | 0.63±0.06 | 0.48±0.01 | 0.36±0.03 |
|  | DHS | *dhs-28* | 0.8±0.1 | 0.6±0.04 | 0.68±0.09 |
|  | DHS | ***dhs-20*** | 0.7±0.21 | 0.32±0.05 | 0.24±0.06 |
|  | FAD binding domain | **F54D5.12** | 0.58±0.05 | 0.38±0.07 | 0.31±0.04 |
|  | FAD binding domain | **F32D8.12** | 0.61±0.19 | 0.53±0.1 | 0.33±0.06 |
|  | NADH:flavin oxidoreductase/12-oxophytodienoate reductase | **T10B5.8** | 0.65±0.16 | 0.26±0.08 | 0.21±0.05 |
|  | FMO | ***fmo-3*** | 0.99±0.21 | 0.67±0.11 | 0.49±0.11 |
|  | GST | ***gst-5*** | 0.86±0.24 | 0.49±0.1 | 0.5±0.12 |
|  | GST | ***gst-6*** | 0.82±0.21 | 0.67±0.12 | 0.44±0.08 |
|  | GST | ***gst-7*** | 0.72±0.23 | 0.39±0.04 | 0.49±0.05 |
|  | GST | *gst-13* | 0.94±0.14 | 0.61±0.06 | 0.54±0.1 |
|  | UGT | ***ugt-1*** | 0.94±0.26 | 0.27±0.08 | 0.13±0.02 |
|  | UGT | *ugt-5* | 1.03±0.11 | 1.37±0.63 | 2.04±1.24 |
|  | UGT | *ugt-6* | 0.95±0.19 | 0.69±0.12 | 0.81±0.22 |
|  | UGT | ***ugt-8*** | 0.76±0.17 | 0.45±0.05 | 0.39±0.01 |
|  | UGT | ***ugt-12*** | 0.6±0.04 | 0.32±0.02 | 0.2±0 |
|  | UGT | ***ugt-13*** | 0.6±0.11 | 0.32±0.02 | 0.22±0.03 |
|  | UGT | ***ugt-17*** | 0.54±0.09 | 0.28±0.12 | 0.21±0.15 |
|  | UGT | ***ugt-25*** | 0.77±0.14 | 0.47±0.02 | 0.36±0.03 |
|  | UGT | ***ugt-26*** | 1.14±0.54 | 0.34±0.03 | 0.37±0.01 |
|  | UGT | *ugt-47* | 0.83±0.07 | 1.11±0.49 | 0.56±0.11 |
|  | UGT | ***ugt-58*** | 0.81±0.04 | 0.6±0.05 | 0.49±0.04 |
|  | UGT | ***ugt-61*** | 1.28±0.15 | 0.36±0.1 | 0.2±0.04 |
|  | UGT | ***ugt-62*** | 0.78±0.23 | 0.31±0.05 | 0.35±0.06 |
|  | UGT | ***ugt-63*** | 0.22±0.12 | 0.01±0 | 0.01±0 |
|  | Reductase | **F25D1.5** | 1.59±1.28 | 0.21±0.09 | 0.31±0.18 |
|  | Small molecule kinase (DUF227, DUF1679) | **T16G1.6** | 0.93±0.47 | 0.19±0.07 | 0.07±0.02 |
|  | Small molecule kinase (DUF227) | **T16G1.7** | 0.9±0.38 | 0.07±0.01 | 0.04±0.01 |
|  | Small molecule kinase (DUF227, DUF1679) | F58B4.5 | 0.77±0.08 | 0.52±0.06 | 0.54±0.13 |
|  | MTL | ***mtl-2*** | 0.46±0.08 | 0.16±0.01 | 0.08±0.04 |
|  | Zn^2+^-Transporter | T18D3.3 | 0.97±0.08 | 0.75±0.07 | 0.6±0.08 |
|  | Se^2+^-binding | **Y37A1B.5** | 0.59±0.08 | 0.43±0.07 | 0.35±0.08 |
|  | ACS | **C01G6.7** | 0.88±0.13 | 0.46±0.03 | 0.42±0.03 |
|  | ACS/FA-transporter | **D1009.1** | 0.71±0.09 | 0.42±0.01 | 0.34±0.09 |
| **METABOLISM** | Cystathionine beta-lyases | **F22B8.6** | 0.39±0.08 | 0.09±0.02 | 0.08±0.03 |
|  | Creatine kinase | **F44G3.2** | 0.36±0.14 | 0.02±0 | 0.01±0.01 |
|  | 3-hydroxyacyl-CoA dehydrogenase | **B0272.3** | 0.82±0.16 | 0.6±0.03 | 0.49±0.05 |
|  | Lipid phosphate phosphatase (PAP) | **T28D9.3** | 0.79±0.17 | 0.49±0.04 | 0.57±0.06 |
|  | Micronutrient transporters (folate transporter family) | **F37B4.7 (*folt-2*)** | 0.33±0.02 | 0.09±0.02 | 0.07±0.01 |
|  | ECH | ***ech-6*** | 0.63±0.05 | 0.32±0.01 | 0.28±0.03 |
|  | TAG-cholesterol-esterase | ZK6.7 | 0.73±0.1 | 0.66±0.06 | 0.54±0.04 |
|  | Phytanoyl-CoA-Hydroxylase | **ZK550.6** | 0.68±0.12 | 0.46±0.16 | 0.38±0.1 |
|  | Hydrolase | **F37H8.3** | 0.59±0.29 | 0.21±0.05 | 0.19±0.07 |
|  | Carbonic anhydrase | ***cah-4*** | 0.69±0.1 | 0.41±0.05 | 0.28±0.09 |
|  | Peroxisoma beta oxidation | *maoc-1* | 0.73±0.09 | 0.52±0.05 | 0.67±0.22 |
|  | Pristanoyl-CoA/acyl-CoA oxidase | F58F9.7 | 0.89±0.23 | 0.66±0.12 | 0.51±0.05 |
|  | Dihydroxyacetone kinase/ glycerone kinase | **W02H5.8** | 0.81±0.05 | 0.56±0.13 | 0.44±0.11 |
|  | TAG-lipase | **F14E5.5** | 0.81±0.02 | 0.45±0.06 | 0.43±0.04 |
|  | Succinyl-CoA:alpha-ketoacid-CoA transferase | **C05C10.3** | 0.77±0.06 | 0.56±0.08 | 0.42±0.04 |
|  | UDP-N-acetylglucosamine transporter | **F15B10.1** | 0.91±0.15 | 0.57±0.01 | 0.49±0.02 |
|  | Medium-chain acyl-CoA dehydrogenase | F28A10.6 | 0.97±0.11 | 1.28±0.44 | 0.61±0.03 |
|  | PEP-CK | **R11A5.4** | 0.83±0.06 | 0.59±0.05 | 0.48±0.04 |
|  | Acetyl-CoA hydrolase | ZK1320.9 | 0.92±0.06 | 0.75±0.02 | 0.68±0.03 |
|  | Peroxisomal 3-ketoacyl-CoA-thiolase P-44/SCP2 | **Y57A10C.6** | 0.67±0.13 | 0.42±0.04 | 0.41±0.05 |
|  | Cytochrome b5 | **C31E10.7** | 0.78±0.13 | 0.48±0.03 | 0.36±0.03 |
| **OTHER FUNCTIONS** | CUB-domain | **T05E12.6** | 0.71±0.04 | 0.49±0.12 | 0.32±0.06 |
|  | CUB-like domain | **C29F3.7** | 0.81±0.13 | 0.32±0.01 | 0.38±0.02 |
|  | D-aspartate oxidase | **F18E3.7** | 0.65±0.06 | 0.37±0.09 | 0.36±0.08 |
|  | Lysozyme | *lys-10* | 1.23±1.06 | 2.13±1.89 | 1.85±1.72 |
|  | Lysozyme | *lys-4* | 0.68±0.04 | 1.02±0.13 | 0.97±0.2 |
|  | Chitin binding Peritrophin-A domain | **R02F2.4** | 0.75±0.27 | 0.49±0.12 | 0.7±0.15 |
|  | DUF141 | **Y38E10A.13** | 0.41±0.06 | 0.19±0.09 | 0.13±0.09 |
|  | DUF1412 | **Y38E10A.12** | 0.49±0.2 | 0.57±0.35 | 0.49±0.2 |
|  | Defense-related protein containing SCP domain | **F15E11.12** | 0.4±0.12 | 0.02±0.01 | 0.01±0.01 |
|  | Defense-related protein containing SCP domain | Y51H7C.12 | 1.24±0.33 | 1.08±0.05 | 1.26±0.31 |
|  | Lysosomal & prostatic acid phosphatases | **F07H5.9** | 1.11±0.03 | 0.76±0.08 | 0.48±0.07 |
|  | Transmembrane olfactory receptor | ZK262.6 *(srw-83)* | 0.88±0.24 | 1.63±0.78 | 1.1±0.55 |
|  | DUF274 | ZK6.11 | 0.9±0.15 | 0.63±0.09 | 0.56±0.06 |
|  | NHR | ***nhr-114*** | 0.81±0.09 | 0.67±0.12 | 0.44±0.05 |
|  | C-type lectin | **Y19D10A.9** | 1.11±0.65 | 0.2±0.09 | 0.14±0.09 |
|  | F-box protein | *fbxa-72* | 1.11±0.05 | 0.84±0.05 | 0.51±0.06 |
| **UNKNOWN** |  | **F45D11.14** | 0.79±0.38 | 0.09±0.01 | 0.05±0.01 |
|  |  | **C23H5.8** | 0.36±0.08 | 0.15±0.04 | 0.09±0.02 |
|  |  | **F21C10.9** | 0.43±0.09 | 0.06±0.01 | 0.03±0.01 |
|  |  | Y71F9B.1 | 0.73±0.09 | 0.58±0.04 | 0.51±0.09 |
|  |  | **M02H5.8** | 0.58±0.09 | 0.24±0.04 | 0.17±0.04 |
|  |  | Y45G12C.1 | 1.12±0.47 | 5.98±5.24 | 4.48±3.76 |
|  |  | **F17C11.6** | 0.68±0.13 | 0.25±0.02 | 0.11±0.03 |
| **CON-TROL** | Actin | *act-1* | 1±0 | 1±0 | 1±0 |
|  | Pol_II_ | *ama-1* | 0.83±0.16 | 0.84±0.04 | 0.88±0.13 |
|  | NHR | *nhr-23* | 1.14±0.04 | 1.35±0.13 | 1.35±0.05 |
