# Supplementary material for: The Mediator Subunit MDT-15 Confers Metabolic Adaptation to Ingested Material
Source: PLoS Genet. 2008 Feb 29;4(2):e1000021. doi: 10.1371/journal.pgen.1000021 (PMC2265483; doi:10.1371/journal.pgen.1000021)
Supplement: Table S3 — Validation of candidate MDT-15 targets in mutant mdt-15(tm2182) worms. QPCR quantification of mRNA levels of 97 known and candidate MDT-15-dependent genes. Values represent fold changes±SEM in mutant mdt-15(tm2182) worms vs. N2 worms, calculated from the average relative mRNA levels from three independent biological replicates (mRNA levels normalized to act-1). 50 genes downregulated more than two-fold in mdt-15(tm2182) worm are in bold. The hacd-1 and cpt-3 genes are upregulated in mutant mdt-15(tm2182) worms, whereas MDT-15 itself and control genes ama-1 and nhr-23 are not significantly regulated, consistent with data from our previous study [14]. Genes are classified into groups with similar biological functions (left column). (0.18 MB DOC) [file pgen.1000021.s007.doc]

*Supporting Table S3: Validation of candidate MDT-15 targets in mutant* mdt-15(tm2182) *worms.*

QPCR quantification of mRNA levels of 97 known and candidate MDT-15-dependent genes. Values represent fold changes ± SEM in mutant *mdt-15(tm2182)* worms *vs.* N2 worms, calculated from the average relative mRNA levels from three independent biological replicates (mRNA levels normalized to *act-1*). 50 genes downregulated more than two-fold in *mdt-15(tm2182)* worm are in **bold**. The *hacd-1* and *cpt-3* genes are upregulated in mutant *mdt-15(tm2182)* worms, whereas *mdt-15* itself and control genes *ama-1* and *nhr-23* are not significantly regulated, consistent with data from our previous study [14]. Genes are classified into groups with similar biological functions (left column).

| **Gene Class** | **Gene function** | **Gene name** | **mRNA level in**  ***mdt-15(tm2182)***  **[*vs.* N2 worms]** |
| --- | --- | --- | --- |
| **KNOWN METABOLISM TARGETS** | Lipid binding protein | ***lbp-8*** | 0.01±0 |
|  | ACS | ***acs-2*** | 0.13±0.05 |
|  | Malate synthase; Isocitrate lyase | ***gei-7*** | 0.32±0.07 |
|  | Fatty acid desaturase | *fat-2* | 0.66±0.13 |
|  | Fatty acid desaturase | ***fat-5*** | 0.13±0.1 |
|  | Fatty acid desaturase | ***fat-6*** | 0.35±0.05 |
|  | Fatty acid desaturase | ***fat-7*** | 0.07±0.05 |
|  | Acyl-CoA oxidase | **F08A8.2** | 0.43±0.04 |
|  | Acyl-CoA oxidase | F08A8.3 | 0.55±0.08 |
|  | Acyl-CoA dehydrogenase | ***acdh-1/dod-12*** | 0.01±0 |
|  | Acyl-CoA dehydrogenase | ***acdh-2*** | 0.05±0.01 |
|  | Carnitine palmitoyl transferase | ***cpt-5*** | 0.05±0.01 |
|  | Hydroxy-acyl CoA dehydrogenase | ***hacd-1*** | 2.03±0.26 |
|  | Carnitine palmitoyl transferase | ***cpt-3*** | 5.00±0.6 |
| **DETOXIFICATION** | ABC-transporter | *pgp-7* | 0.63±0.22 |
|  | ABC-transporter | ***pmp-5*** | 0.24±0.08 |
|  | Aldehyde dehydrogenase | **Y38F1A.6** | 0.09±0.02 |
|  | Aldehyde dehydrogenase | *alh-5* | 0.5±0.1 |
|  | CYP450 | ***cyp-35C1*** | 0.23±0.05 |
|  | DHS | *dhs-3* | 0.98±0.07 |
|  | DHS | ***dhs-25*** | 0.26±0.05 |
|  | DHS | *dhs-28* | 0.67±0.05 |
|  | DHS | *dhs-20* | 2.94±1.4 |
|  | FAD binding domain | **F54D5.12** | 0.26±0.06 |
|  | FAD binding domain | **F32D8.12** | 0.28±0.06 |
|  | NADH:flavin oxidoreductase/12-oxophytodienoate reductase | T10B5.8 | 0.5±0.11 |
|  | FMO | *fmo-3* | 0.59±0.12 |
|  | GST | ***gst-5*** | 0.42±0.07 |
|  | GST | ***gst-6*** | 0.32±0.17 |
|  | GST | *gst-7* | 0.54±0.06 |
|  | GST | *gst-13* | 0.59±0.18 |
|  | GST | *gst-13* | 0.63±0.1 |
|  | UGT | ***ugt-1*** | 0.14±0.09 |
|  | UGT | *ugt-5* | 0.71±0.13 |
|  | UGT | *ugt-6* | 0.61±0.21 |
|  | UGT | ***ugt-8*** | 0.34±0.04 |
|  | UGT | ***ugt-12*** | 0.39±0.1 |
|  | UGT | *ugt-13* | 0.56±0.15 |
|  | UGT | ***ugt-17*** | 0.32±0.16 |
|  | UGT | ***ugt-25*** | 0.36±0.02 |
|  | UGT | ***ugt-26*** | 0.47±0.06 |
|  | UGT | *ugt-47* | 0.83±0.09 |
|  | UGT | *ugt-58* | 0.66±0.01 |
|  | UGT | *ugt-61* | 0.81±0.21 |
|  | UGT | *ugt-62* | 0.63±0.13 |
|  | UGT | ***ugt-63*** | 0.16±0.05 |
|  | Reductase | F25D1.5 | 1.16±0.25 |
|  | Small molecule kinase (DUF227, DUF1679) | **T16G1.6** | 0.08±0.03 |
|  | Small molecule kinase (DUF227) | **T16G1.7** | 0.1±0.03 |
|  | Small molecule kinase (DUF227, DUF1679) | **F58B4.5** | 0.43±0.07 |
|  | MTL | ***mtl-2*** | 0.16±0.04 |
|  | Zn^2+^-Transporter | T18D3.3 | 0.7±0.06 |
|  | Se^2+^-binding | **Y37A1B.5** | 0.13±0.06 |
|  | ACS | C01G6.7 | 0.54±0.08 |
|  | ACS/FA-transporter | **D1009.1** | 0.48±0.02 |
| **METABOLISM** | Cystathionine beta-lyases | **F22B8.6** | 0.07±0 |
|  | Creatine kinase | **F44G3.2** | 0.01±0.01 |
|  | 3-hydroxyacyl-CoA dehydrogenase | B0272.3 | 0.55±0.09 |
|  | Lipid phosphate phosphatase (PAP) | **T28D9.3** | 0.34±0.12 |
|  | Micronutrient transporters (folate transporter family) | **F37B4.7(*folt-2*)** | 0.19±0.02 |
|  | ECH | ***ech-6*** | 0.2±0.01 |
|  | TAG-cholesterol-esterase | ZK6.7 | 0.87±0.12 |
|  | Phytanoyl-CoA-Hydroxylase | ZK550.6 | 0.58±0.07 |
|  | Hydrolase | **F37H8.3** | 0.2±0.05 |
|  | Carbonic anhydrase | ***cah-4*** | 0.27±0.07 |
|  | Peroxisomal beta oxidation | *maoc-1* | 0.59±0.07 |
|  | Pristanoyl-CoA/acyl-CoA oxidase | F58F9.7 | 0.62±0.15 |
|  | Dihydroxyacetone kinase/glycerone kinase | W02H5.8 | 0.7±0.02 |
|  | TAG-lipase | F14E5.5 | 0.61±0.1 |
|  | Succinyl-CoA:alpha-ketoacid-CoA transferase | **C05C10.3** | 0.49±0.1 |
|  | UDP-N-acetylglucosamine transporter | F15B10.1 | 0.83±0.12 |
|  | Medium-chain acyl-CoA dehydrogenase | F28A10.6 | 1.16±0.39 |
|  | PEP-CK | R11A5.4 | 0.73±0.12 |
|  | Acetyl-CoA hydrolase | ZK1320.9 | 0.62±0.09 |
|  | Peroxisomal 3-ketoacyl-CoA-thiolase P-44/SCP2 | **Y57A10C.6** | 0.41±0.09 |
|  | Cytochrome b5 | **C31E10.7** | 0.4±0.06 |
| **OTHER** **FUNCTION** | CUB-domain | **T05E12.6** | 0.41±0.06 |
|  | CUB-like domain | C29F3.7 | 0.57±0.21 |
|  | D-aspartate oxidase | **F18E3.7** | 0.23±0.01 |
|  | Lysozyme | *lys-10* | 1.46±0.82 |
|  | Lysozyme | ***lys-4*** | 0.48±0.32 |
|  | Chitin binding Peritrophin-A domain | R02F2.4 | 1.47±0.29 |
|  | DUF141 | **Y38E10A.13** | 0.09±0.04 |
|  | DUF1412 | **Y38E10A.12** | 0.11±0.05 |
|  | Defense-related protein containing SCP domain | **F15E11.12** | 0.04±0.03 |
|  | Defense-related protein containing SCP domain | Y51H7C.12 | 1.58±0.7 |
|  | Lysosomal & prostatic acid phosphatases | F07H5.9 | 0.55±0.09 |
|  | Transmembrane olfactory receptor | ZK262.6 | 1.08±0.24 |
|  | DUF274 | ZK6.11 | 1.13±0.03 |
|  | NHR | *nhr-114* | 0.71±0.21 |
|  | C-type lectin | Y19D10A.9 | 0.95±0.28 |
|  | F-box protein | ***fbxa-72*** | 0.34±0.09 |
| **UNKNOWN** |  | **F45D11.14** | 0.13±0.06 |
|  |  | **C23H5.8** | 0.16±0.05 |
|  |  | **F21C10.9** | 0.06±0.01 |
|  |  | Y71F9B.1 | 0.83±0.22 |
|  |  | M02H5.8 | 0.85±0.17 |
|  |  | Y45G12C.1 | 0.68±0.08 |
|  |  | F17C11.6 | 0.63±0.14 |
| **CONTROLS** | Mediator subunit | *mdt-15* | 1.01±0.13 |
|  | Actin | *act-1* | 1±0 |
|  | Pol_II_ | *ama-1* | 1.12±0.23 |
|  | NHR | *nhr-23* | 1.13±0.08 |
