# Supplementary material for: The Mediator Subunit MDT-15 Confers Metabolic Adaptation to Ingested Material
Source: PLoS Genet. 2008 Feb 29;4(2):e1000021. doi: 10.1371/journal.pgen.1000021 (PMC2265483; doi:10.1371/journal.pgen.1000021)
Supplement: Table S4 — Occurrence of gene families and protein domains in microarray results based on GO terms. Occurrence of gene ontology terms and protein domains (based on InterPro annotation) in genes that, by microarray analysis, were found to be upregulated in mdt-15(RNAi) worms (vs. control(RNAi) worms), ranked by lowest P-value. 100 genes (of 120 total) were not classified by gene functional category analysis; 79 genes were not classified by the protein domain analysis. “Count” indicates the number of genes amongst MDT-15 targets that fit the respective term. “%” indicates the percentage of these amongst all MDT-15 targets. “P-value” indicates the statistical significance of the overrepresentation of an individual category; we used a P-value of 0.05 as cutoff. (0.05 MB DOC) [file pgen.1000021.s008.doc]

*Supporting Table S4: Occurrence of gene families and protein domains in microarray results based on GO terms.*

Occurrence of gene ontology terms and protein domains (based on InterPro annotation) in genes that, by microarray analysis, were found to be upregulated in *mdt-15(RNAi)* worms (*vs.* *control(RNAi)* worms), ranked by lowest *P*-value. 100 genes (of 120 total) were not classified by gene functional category analysis; 79 genes were not classified by the protein domain analysis. “Count” indicates the number of genes amongst MDT-15 targets that fit the respective term. “%” indicates the percentage of these amongst all MDT-15 targets. “*P*-Value” indicates the statistical significance of the overrepresentation of an individual category; we used a *P*-value of 0.05 as cutoff.

| **Gene functional category** | **Count** | **%** | ***P*-Value** |
| --- | --- | --- | --- |
| Lipid metabolism | 5 | 4.7% | 1.2x10^-2^ |
| Lipid catabolism | 3 | 2.8% | 1.3x10^-2^ |
| Primary metabolism | 20 | 18.9% | 2.4x10^-2^ |
| Protein folding | 3 | 2.8% | 4.0x10^-2^ |
| **Protein domain** | **Count** | **%** | ***P*-Value** |
| CUB | 10 | 9.4% | 5.6x10^-9^ |
| Saposin B | 4 | 3.8% | 5.9x10^-4^ |
| SKP1 component | 3 | 2.8% | 1.0x10^-2^ |
| Lipase, active site | 3 | 2.8% | 1.9x10^-2^ |
| Glutathione S-transferase, N-terminal | 3 | 2.8% | 3.8x10^-2^ |
