# Supplementary material for: The Mediator Subunit MDT-15 Confers Metabolic Adaptation to Ingested Material
Source: PLoS Genet. 2008 Feb 29;4(2):e1000021. doi: 10.1371/journal.pgen.1000021 (PMC2265483; doi:10.1371/journal.pgen.1000021)
Supplement: Table S5 — Many genes downregulated after MDT-15 depletion are intestine-enriched. Comparison of genes downregulated after MDT-15 depletion (as determined by microarray analysis) and groups of genes expressed in tissue-restricted manner. Columns contain WormBase accession numbers of MDT-15 target genes with respective tissue specific expression. Intestine-, muscle-, and germline-enriched genes are from Pauli et al. [31], and pharynx-enriched genes from Gaudet et al. [32]. (0.09 MB DOC) [file pgen.1000021.s009.doc]

*Supporting Table S5: Many genes downregulated after MDT-15 depletion are intestine-enriched.*

Comparison of genes downregulated after MDT-15 depletion (as determined by microarray analysis) and groups of genes expressed in tissue-restricted manner. Columns contain WormBase accession numbers of MDT-15 target genes with respective tissue specific expression. Intestine-, muscle-, and germline-enriched genes are from Pauli et al. [31], and pharynx-enriched genes from Gaudet et al. [32].

| **Intestine-enriched genes (624 total)** | **Muscle-enriched genes (230 total)** | **Germline-enriched genes (1135 total)** | **Pharynx-specific genes (339 total)** |
| --- | --- | --- | --- |
| **43 in *mdt-15(RNAi)*** | **None in *mdt-15(RNAi)*** | **None in *mdt-15(RNAi)*** | **Four in *mdt-15(RNAi)*** |
| T05E12.6 |  |  | W06D12.3 |
| AC3.7 |  |  | F11G11.2 |
| F58B4.5 |  |  | R03D7.6 |
| K09H11.7 |  |  | T26C5.1 |
| F17E9.11 |  |  |  |
| T19H12.9 |  |  |  |
| R04B5.9 |  |  |  |
| F08A8.2 |  |  |  |
| F08A8.4 |  |  |  |
| C43H6.8 |  |  |  |
| C29F7.2 |  |  |  |
| E04F6.3 |  |  |  |
| F15B10.1 |  |  |  |
| F18E2.1 |  |  |  |
| D1009.1 |  |  |  |
| C33A12.6 |  |  |  |
| F35E12.6 |  |  |  |
| C01G6.7 |  |  |  |
| F25D1.5 |  |  |  |
| B0041.6 |  |  |  |
| T22G5.2 |  |  |  |
| F17C11.6 |  |  |  |
| ZK6.11 |  |  |  |
| C29F9.3 |  |  |  |
| F31F7.1 |  |  |  |
| F18E3.7 |  |  |  |
| F14E5.5 |  |  |  |
| F31F4.15 |  |  |  |
| T16G1.7 |  |  |  |
| Y32F6B.1 |  |  |  |
| F10D2.9 |  |  |  |
| C29F3.7 |  |  |  |
| F39G3.1 |  |  |  |
| R151.2 |  |  |  |
| F58G6.2 |  |  |  |
| F59B1.8 |  |  |  |
| F41E7.4 |  |  |  |
| ZK945.1 |  |  |  |
| F36A2.3 |  |  |  |
| F58F9.7 |  |  |  |
| ZK1307.1 |  |  |  |
| F11G11.3 |  |  |  |
| C02C2.4 |  |  |  |
