# Supplementary material for: The Mediator Subunit MDT-15 Confers Metabolic Adaptation to Ingested Material
Source: PLoS Genet. 2008 Feb 29;4(2):e1000021. doi: 10.1371/journal.pgen.1000021 (PMC2265483; doi:10.1371/journal.pgen.1000021)
Supplement: Table S6 — Basal and toxin-induced expression of some cyp genes (encoding CYP450s) is reduced in mdt-15(RNAi) worms. QPCR quantification of mRNA levels of cyp genes. Values represent fold changes±SEM in mdt-15(RNAi) worms vs. control(RNAi) worms, calculated from the average relative mRNA levels from three independent biological replicates (mRNA levels normalized to act-1). FLA = fluoranthene; NF = β-naphthoflavone; D = DMSO. Columns on the right indicate the fold-induction by toxins vs. DMSO in the same genetic condition; several genes exhibit reduced fold-induction upon MDT-15 depletion. cyp genes whose expression is toxin-induced are listed in the top section, unresponsive cyp genes in the middle section; in both sections, bold font indicates MDT-15 dependence. Note that some genes are MDT-15-dependent for basal and induced transcription. (0.16 MB DOC) [file pgen.1000021.s010.doc]

*Supporting Table S6: Basal and toxin-induced expression of some* cyp *genes (encoding CYP450s) is reduced in* mdt-15(RNAi) *worms.*

QPCR quantification of mRNA levels of *cyp* genes. Values represent fold changes ± SEM in *mdt-15(RNAi)* worms *vs.* *control(RNAi)* worms, calculated from the average relative mRNA levels from three independent biological replicates (mRNA levels normalized to *act-1*). FLA = fluoranthene; NF = β-naphtoflavone; D = DMSO. Columns on the right indicate the fold-induction by toxins *vs.* DMSO in the same genetic condition; several genes exhibit reduced fold-induction upon MDT-15 depletion. *cyp* genes whose expression is toxin-induced are listed in the top section, unresponsive *cyp* genes in the middle section; in both sections, **bold** font indicates MDT-15 dependence. Note that some genes are MDT-15-dependent for basal and induced transcription.

|  |  | **Overall fold change *vs.* control RNAi** | | | | | | **Fold change caused by toxin *vs.* solvent (within RNAi condition)** | | | |
| --- | --- | --- | --- | --- | --- | --- | --- | --- | --- | --- | --- |
|  | **RNAi** | ***con-trol*** | ***control*** | ***control*** | ***mdt-15*** | ***mdt-15*** | ***mdt-15*** | ***con-trol*** | ***mdt-15*** | ***con-trol*** | ***mdt-15*** |
|  | **Toxin** | **DM-SO** | **FLA** | **NF** | **DMSO** | **FLA** | **NF** | **FLA**  ***vs.* D** | **FLA**  ***vs.* D** | **NF**  ***vs.* D** | **NF**  ***vs.* D** |
| TOXIN-RESPONSIVE; **BOLD** = MDT-15 DEPENDENT INDUCTION | ***cyp-13B1*** | 1±0 | 31.1±14.7 | 6.6±2.4 | 0.1±0.05 | 0.9±0.3 | 0.4±0.2 | 31.1 | 8.3 | 6.6 | 3.5 |
|  | ***cyp-14A3*** | 1±0 | 14.1±4.5 | 1.4±1.1 | 0.7±0.2 | 2.1±1.1 | 0.6±0.2 | 14.1 | 3.2 | 1.4 | 1.0 |
|  | *cyp-25A5* | 1±0 | 2±0.6 | 1.4±0.7 | 1.3±0.3 | 2.3±0.8 | 1.4±0.7 | 2.0 | 1.8 | 1.4 | 1.0 |
|  | *cyp-29A2* | 1±0 | 2.6±1 | 1.6±0.4 | 0.3±0.05 | 0.3±0.1 | 0.3±0.1 | 2.6 | 1.3 | 1.6 | 1.3 |
|  | ***cyp-33B1*** | 1±0 | 20.0±9.4 | 5.0±0.5 | 3.2±1.2 | 2.7±0.1 | 7.4±4.5 | 20.0 | 0.8 | 5.0 | 2.3 |
|  | *cyp-34A9* | 1±0 | 4.3±1.4 | 1.8±0.3 | 0.1±0.1 | 0.4±0.1 | 0.2±0.2 | 4.3 | 5.0 | 1.8 | 2.4 |
|  | *cyp-34A10* | 1±0 | 34.8±7.2 | 1.7±0.8 | 0.1±0.1 | 3.4±2.8 | 0.1±0.04 | 34.8 | 42.1 | 1.7 | 1.1 |
|  | ***cyp-35A1*** | 1±0 | 154.3±52.9 | 31.6±15. 6 | 0.2±0.2 | 5.8±5.1 | 0.9±0.3 | 154.3 | 29.1 | 31.6 | 4.6 |
|  | ***cyp-35A2*** | 1±0 | 6.5±2.5 | 12.2±3.3 | 0.6±0.2 | 0.4±0.1 | 2.1±1.2 | 6.5 | 0.6 | 12.2 | 3.7 |
|  | ***cyp-35A3*** | 1±0 | 13.6±6.2 | 54.1±31.5 | 0.2±0.1 | 0.2±0.1 | 2.4±1.1 | 13.6 | 1.2 | 54.1 | 15.7 |
|  | ***cyp-35B1*** | 1±0 | 12.9±4.7 | 6.3±1.0 | 0.2±0.1 | 1.4±0.3 | 0.8±0.2 | 12.9 | 5.6 | 6.3 | 3.4 |
|  | ***cyp-35B2*** | 1±0 | 72.2±38.6 | 26.3±4.7 | 0.6±0.4 | 1.1±0.3 | 1.1±0.4 | 72.2 | 2.0 | 26.3 | 1.9 |
|  | ***cyp-35C1*** | 1±0 | 28.7±11.8 | 45.4±16.6 | 0.2±0.1 | 2.2±1.4 | 6.3±3.6 | 28.7 | 14.5 | 45.4 | 42.1 |
|  | *cyp-36A1* | 1±0 | 2.3±0.8 | 1.3±0.2 | 0.5±0.1 | 0.8±0.2 | 0.6±0.2 | 2.3 | 1.7 | 1.3 | 1.3 |
|  | *cyp-37B1* | 1±0 | 4.5±1.2 | 1.9±0.2 | 0.7±0.2 | 1.4±0.3 | 0.8±0.3 | 4.5 | 2.0 | 1.9 | 1.2 |
| NOT TOXINS RESPONSIVE; **BOLD** = MDT-15 DEPENDENT IN BASAL STATE | *cyp-13A1* | 1±0 | 0.8±0.1 | 1.1±0.2 | 3.8±1.3 | 4.5±1.0 | 4.0±1.7 | 0.8 | 1.2 | 1.1 | 1.1 |
|  | *cyp-13A2* | 1±0 | 1.8±0.3 | 1.2±0.4 | 0.7±0.4 | 0.4±0.1 | 0.6±0.2 | 1.3 | 0.6 | 1.2 | 0.8 |
|  | *cyp-13A3* | 1±0 | 1.3±0.1 | 1.2±0.3 | 1.5±0.1 | 1.4±0.2 | 1.5±0.1 | 1.3 | 0.9 | 1.2 | 1.0 |
|  | ***cyp-13A6*** | 1±0 | 0.6±0.1 | 1.0±0.4 | 0.1±0.1 | 0.1±0.1 | 0.1±0.1 | 0.6 | 2.0 | 0.9 | 2.0 |
|  | ***cyp-13A7*** | 1±0 | 0.8±0.4 | 1.3±0.7 | 0.2±0.1 | 0.2±0.1 | 0.1±0.1 | 0.8 | 1.2 | 1.3 | 0.7 |
|  | *cyp-13A10* | 1±0 | 0.7±0.2 | 0.7±0.1 | 0.7±0.2 | 0.4±0.1 | 0.3±0.1 | 0.7 | 0.6 | 0.8 | 0.5 |
|  | *cyp-14A5* | 1±0 | 0.9±0.2 | 1.0±0.3 | 0.9±0.3 | 1.0±0.3 | 0.8±0.3 | 0.9 | 1.1 | 1.0 | 0.9 |
|  | ***cyp-25A1*** | 1±0 | 0.2±0.1 | 0.3±0.1 | 0.1±0.1 | 0±0 | 0±0 | 0.2 | 0.0 | 0.3 | 0.0 |
|  | ***cyp-25A2*** | 1±0 | 0.4±0.1 | 0.6±0.2 | 0.1±0.1 | 0.2±0.1 | 0.1±0.1 | 0.4 | 1.6 | 0.6 | 0.9 |
|  | *cyp-25A4* | 1±0 | 1.1±0.2 | 1.0±0.1 | 1.2±0.2 | 1.6±0.2 | 1.3±0.2 | 1.1 | 1.3 | 1.0 | 1.0 |
|  | *cyp-25A6* | 1±0 | 1.6±0.5 | 1.2±0.6 | 0.9±0.4 | 1.6±0.6 | 0.7±0.4 | 1.6 | 1.7 | 1.2 | 0.8 |
|  | *cyp-29A1* | 1±0 | 1.2±0.2 | 1.2±0.1 | 1.4±0.4 | 1.4±0.4 | 1.4±0.4 | 1.2 | 1.0 | 1.2 | 1.0 |
|  | *cyp-29A4* | 1±0 | 0.7±0.2 | 0.9±0.4 | 0.8±0.2 | 1.0±0.4 | 0.9±0.4 | 0.7 | 1.3 | 0.9 | 1.1 |
|  | *cyp-31A1* | 1±0 | 1.4±0.4 | 1.2±0.2 | 1.5±0.5 | 1.4±0.3 | 1.3±0.3 | 1.4 | 1.0 | 1.2 | 0.9 |
|  | *cyp-33A1* | 1±0 | 1.1±0.2 | 0.9±0.2 | 0.8±0.1 | 1.0±0.1 | 0.7±0.1 | 1.1 | 1.3 | 0.9 | 1.0 |
|  | *cyp-33C4* | 1±0 | 1.1±0.2 | 1.0±0.3 | 1.0±0.3 | 1.2±0.2 | 1.4±0.6 | 1.1 | 1.1 | 1.0 | 1.3 |
|  | *cyp-33C5* | 1±0 | 1.5±0.3 | 1.2±0.3 | 1.3±0.3 | 2.6±0.4 | 1.9±0.2 | 1.5 | 1.9 | 1.2 | 1.4 |
|  | *cyp-33C6* | 1±0 | 1.7±0.4 | 1.2±0.1 | 0.7±0.2 | 2.3±0.6 | 1.3±0.4 | 1.7 | 3.2 | 1.2 | 1.8 |
|  | *cyp-33C7* | 1±0 | 0.7±0.2 | 1.0±0.3 | 1.6±0.4 | 1.8±0.8 | 1.7±0.6 | 0.7 | 1.2 | 1.0 | 1.1 |
|  | *cyp-33C8* | 1±0 | 1.5±0.4 | 1.6±0.5 | 0.9±0.3 | 1.6±0.5 | 0.9±0.2 | 1.5 | 1.8 | 1.6 | 1.0 |
|  | *cyp-33C11* | 1±0 | 1.5±0.5 | 1.0±0.2 | 1.1±0.2 | 1.1±0.2 | 1.0±0.3 | 1.5 | 1.0 | 1.0 | 0.9 |
|  | *cyp-33E2* | 1±0 | 1.1±0.2 | 1.2±0.2 | 0.7±0.1 | 0.6±0.2 | 0.9±0.1 | 1.1 | 0.8 | 1.2 | 1.2 |
|  | *cyp-33E3a* | 1±0 | 1.9±0.6 | 1.5±0.4 | 1.0±0.3 | 0.7±0.1 | 1.1±0.3 | 1.9 | 0.7 | 1.5 | 1.2 |
|  | *cyp-34A2* | 1±0 | 0.5±0.1 | 0.6±0.1 | 1.0±0.1 | 1.3±0.2 | 0.9±0.1 | 0.5 | 1.3 | 0.6 | 1.0 |
|  | ***cyp-34A4*** | 1±0 | 0.6±0.2 | 0.8±0.2 | 0.4±0.1 | 0.1±0.1 | 0.4±0.1 | 0.6 | 0.4 | 0.8 | 1.3 |
|  | *cyp-34A5* | 1±0 | 0.7±0.1 | 1.3±0.1 | 0.9±0.2 | 0.7±0.1 | 0.8±0.1 | 0.7 | 0.7 | 1.3 | 0.9 |
|  | *cyp-37A1* | 1±0 | 1.6±0.2 | 1.6±0.2 | 1.0±0.3 | 0.8±0.1 | 0.6±0.1 | 1.6 | 0.8 | 1.6 | 0.6 |
|  | *cyp-44A* | 1±0 | 0.8±0.3 | 0.8±0.1 | 1.1±0.2 | 0.7±0.2 | 0.9±0.1 | 0.8 | 0.7 | 0.8 | 0.9 |
| CONTROL GENES | *nhr-23* | 1±0 | 1.1±0.3 | 0.9±0.2 | 1.1±0.2 | 1.1±0.3 | 0.9±0.2 | 1.1 | 1.0 | 0.9 | 0.9 |
|  | *rps-22* | 1±0 | 0.9±0.1 | 0.9±0.1 | 1.0±0.1 | 0.9±0.1 | 0.9±0.1 | 0.9 | 1.0 | 0.9 | 0.9 |
|  | *ctl-1* | 1±0 | 1.1±0.2 | 1.0±0.1 | 1.1±0.1 | 1.4±0.3 | 1.0±0.3 | 1.1 | 1.3 | 1.0 | 0.9 |
|  | *rps-15* | 1±0 | 0.8±0.1 | 0.9±0.1 | 0.9±0.1 | 0.9±0.1 | 0.7±0.9 | 0.8 | 1.0 | 0.9 | 0.8 |
|  | *rps-22* | 1±0 | 0.9±0.1 | 0.9±0.1 | 0.9±0.1 | 0.8±0.1 | 0.8±0.1 | 0.9 | 0.9 | 0.9 | 0.9 |
|  | *rps-11* | 1±0 | 0.9±0.1 | 0.9±0.1 | 0.9±0.1 | 0.9±0.1 | 0.9±0.1 | 0.9 | 1.0 | 0.9 | 1.0 |
|  | *fat-5* | 1±0 | 0.2±0.1 | 0.4±0.1 | 0.1±0.1 | 0.1±0.1 | 0.1±0.1 | 0.2 | 0.6 | 0.4 | 1.1 |
|  | *fat-6* | 1±0 | 0.7±0.2 | 1.0±0.1 | 0.1±0.1 | 0.1±0.1 | 0.1±0.1 | 0.7 | 0.6 | 1.0 | 0.6 |
|  | *mdt-15* | 1±0 | 1.2±0.3 | 1.0±0.2 | 0.3±0.1 | 0.5±0.1 | 0.4±0.1 | 1.2 | 1.4 | 1.0 | 1.0 |
