# Supplementary material for: The Mediator Subunit MDT-15 Confers Metabolic Adaptation to Ingested Material
Source: PLoS Genet. 2008 Feb 29;4(2):e1000021. doi: 10.1371/journal.pgen.1000021 (PMC2265483; doi:10.1371/journal.pgen.1000021)
Supplement: Table S7 — Expression of toxin-induced MDT-15 targets is largely unaffected in mdt-6(RNAi) worms. QPCR quantification of mRNA levels of MDT-15-dependent detoxification genes. Values represent fold changes±SEM in mdt-6(RNAi) worms vs. control(RNAi) worms, calculated from the average relative mRNA levels from three independent biological replicates (mRNA levels normalized to act-1). FLA = fluoranthene; NF = β-naphtoflavone. (0.08 MB DOC) [file pgen.1000021.s011.doc]

*Supporting Table S7:* *Expression of toxin-induced MDT-15 targets is largely unaffected in* mdt-6(RNAi) *worms.*

QPCR quantification of mRNA levels of MDT-15-dependent detoxification genes. Values represent fold changes ± SEM in *mdt-6(RNAi)* worms *vs.* *control(RNAi)* worms, calculated from the average relative mRNA levels from three independent biological replicates (mRNA levels normalized to *act-1*). FLA = fluoranthene; NF = β-naphtoflavone.

| **Gene function** | **RNAi clone** | ***control*** | ***mdt-6*** | ***control*** | ***mdt-6*** | ***control*** | ***mdt-6*** |
| --- | --- | --- | --- | --- | --- | --- | --- |
|  | **Toxin** | **DMSO** | **DMSO** | **FLA** | **FLA** | **NF** | **NF** |
| Hydrolase | F37H8.3 | 1±0 | 1.8±0.69 | 3.97±0.35 | 4.67±0.15 | 0.88±0.12 | 1.04±0.21 |
| ADH | *alh-5* | 1±0 | 0.95±0.46 | 7.53±3.09 | 6.17±2.75 | 2.58±0.87 | 1.91±0.71 |
| UGT | *ugt-58* | 1±0 | 1.5±0.4 | 2.81±0.25 | 3.37±0.27 | 1.41±0.06 | 1.7±0.24 |
| UGT | *ugt-25* | 1±0 | 2.16±0.52 | 5.41±1.67 | 8.86±2.07 | 2.82±0.23 | 4.69±1.1 |
| UGT | *ugt-17* | 1±0 | 0.45±0.11 | 1.71±0.19 | 0.61±0.02 | 1.53±0.26 | 0.56±0.02 |
| UGT | *ugt-13* | 1±0 | 2.8±0.54 | 9.41±1.52 | 8.56±0.51 | 5.45±1.15 | 5.08±0.42 |
| GST | *gst-5* | 1±0 | 4.3±1.17 | 7.26±1.84 | 11.08±2.43 | 1.66±0.25 | 5.29±0.58 |
| UGT | *ugt-8* | 1±0 | 2.46±0.85 | 22.09±2.28 | 21.62±3.79 | 5.8±0.46 | 6.57±0.88 |
| Lipid phosphate phosphatase | T28D9.3 | 1±0 | 1.55±0.3 | 3.35±0.4 | 3.62±0.31 | 1.56±0.13 | 1.89±0.32 |
| CYP450 | *cyp-35C1* | 1±0 | 1.33±0.47 | 24.84±7.32 | 27.36±11.53 | 30.14±8.5 | 44.36±21.5 |
| TAG lipase | F14E5.5 | 1±0 | 1.93±0.65 | 5.08±0.59 | 5.6±0.65 | 2.23±0.37 | 2.68±0.68 |
| CUB-like domain | C29F3.7 | 1±0 | 1.48±0.39 | 4.7±0.47 | 5.2±0.54 | 1.74±0.22 | 2.76±0.39 |
| UDP-N-acetyl-glucosamine transporter | F15B10.1 | 1±0 | 1.27±0.48 | 4.81±0.7 | 4.11±0.82 | 2.09±0.11 | 1.94±0.12 |
| C-type lectin | Y19D10A.9 | 1±0 | 2.1±1.75 | 14.93±4.14 | 10.93±3.91 | 2.44±0.41 | 3.79±1.29 |
| NADH: flavin oxidoreductase/ 12-oxophyto-dienoate reductase | T10B5.8 | 1±0 | 1.16±0.79 | 16.89±1.21 | 12.62±2.75 | 1.37±0.18 | 1.01±0.11 |
| Reductase | F25D1.5 | 1±0 | 3.05±1.7 | 2.35±1.01 | 3.68±1.79 | 1.34±0.27 | 5.37±3.95 |
| Cytochrome b5 | C31E10.7 | 1±0 | 0.76±0.07 | 2.63±0.55 | 2.91±0.58 | 1.66±0.24 | 2.13±0.24 |
| FAD-domain | F32D8.12 | 1±0 | 0.83±0.23 | 2.77±1.05 | 1.73±0.69 | 2.09±0.48 | 1.53±0.68 |
| UGT | *ugt-63* | 1±0 | 0.47±0.21 | 5.59±1.23 | 4.59±0.78 | 2.55±0.49 | 2.29±0.85 |
| Actin | *act-1* | 1±0 | 1±0 | 1±0 | 1±0 | 1±0 | 1±0 |
| NHR | *nhr-23* | 1±0 | 1.6±0.4 | 1.31±0.42 | 1.00±0.22 | 1.4±0.71 | 1.31±0.51 |
|  | *fat-5* | 1±0 | 0.77±0.3 | 0.11±0.1 | 0.05±0.01 | 0.54±0.21 | 0.52±0.1 |
|  | *fat-6* | 1±0 | 0.43±0.19 | 0.67±0.34 | 0.27±0.17 | 1.3±0.31 | 0.71±0.3 |
|  | *fat-7* | 1±0 | 0.08±0.04 | 0.04±0.03 | 0.01±0 | 0.65±0.16 | 0.15±0.09 |
