# Supplementary material for: The Mediator Subunit MDT-15 Confers Metabolic Adaptation to Ingested Material
Source: PLoS Genet. 2008 Feb 29;4(2):e1000021. doi: 10.1371/journal.pgen.1000021 (PMC2265483; doi:10.1371/journal.pgen.1000021)
Supplement: Table S8 — The toxin response is largely unaffected in L4 stage CF512 and nhr-8(ok186) worms. QPCR quantification of mRNA levels of MDT-15 dependent detoxification genes. Values represent fold changes±SEM in CF512 or nhr-8(ok186) worms vs. N2 worms, calculated from the average relative mRNA levels from three independent biological replicates (mRNA levels normalized to act-1). FLA = fluoranthene; NF = β-naphtoflavone. (0.10 MB DOC) [file pgen.1000021.s012.doc]

*Supporting Table S8: The toxin response is largely unaffected in L4 stage CF512 and* nhr-8(ok186) *worms.*

QPCR quantification of mRNA levels of MDT-15 dependent detoxification genes. Values represent fold changes ± SEM in CF512 or *nhr-8(ok186)* worms *vs.* N2 worms, calculated from the average relative mRNA levels from three independent biological replicates (mRNA levels normalized to *act-1*). FLA = fluoranthene; NF = β-naphtoflavone.

| **Gene function** | **Strain** | **N2** | **N2** | **N2** | **CF512** | **CF512** | **CF512** |
| --- | --- | --- | --- | --- | --- | --- | --- |
|  | **Toxin** | **DMSO** | **FLA** | **NF** | **DMSO** | **FLA** | **NF** |
| UGT | *ugt-8* | 1±0 | 44.1±8.3 | 13.9±4.6 | 1.4±0.3 | 29.8±12.9 | 11±9.4 |
| UGT | *ugt-13* | 1±0 | 10.6±4.2 | 4.1±0.5 | 1.5±0.4 | 7±3.3 | 2.4±0 |
| UGT | *ugt-25* | 1±0 | 6.6±0.6 | 3.6±0.5 | 1±0.1 | 5.7±1.3 | 3.1±0.9 |
| UGT | *ugt-58* | 1±0 | 4.9±1.6 | 2.1±0.7 | 2.8±1.9 | 4.2±2.2 | 0.9±0.1 |
| ADH | *alh-5* | 1±0 | 7.4±2.7 | 5.1±1 | 0.6±0.1 | 4.2±1.3 | 2.4±1.1 |
| CYP450 | *cyp-35C1* | 1±0 | 24.6±7.4 | 49.5±24.8 | 1.1±0.2 | 14.5±6.1 | 18±7.6 |
| GST | *gst-5* | 1±0 | 10.9±3.1 | 1.9±0.5 | 0.9±0.3 | 8.6±3.3 | 1±0.2 |
| SMK | T16G1.6 | 1±0 | 7.3±2.7 | 6.2±3.4 | 2.1±1.2 | 5.5±2.1 | 1.5±0.5 |
| Reductase | F25D1.5 | 1±0 | 1±0.3 | 3±0.4 | 0.6±0.2 | 5.7±2.8 | 4.3±1.5 |
| NADH: flavin oxido-reductase/ 12-oxophyto-dienoate reductase | T10B5.8 | 1±0 | 9.6±3.5 | 2.8±0.3 | 1.7±0.9 | 7.2±1.9 | 1.8±0.7 |
| TAG lipase | F14E5.5 | 1±0 | 6±0.8 | 3.1±0.5 | 0.9±0.1 | 4.1±0.9 | 2±0.7 |
| Lipid phosphate phosphatase | T28D9.3 | 1±0 | 2.8±0.1 | 2±0.2 | 0.8±0.1 | 2.7±0.7 | 1.3±0.7 |
| Cytochrome b5 | C31E10.7 | 1±0 | 5.8±0.7 | 4.5±2.2 | 1.6±0.3 | 4.9±1.4 | 3.1±1.8 |
| UDP-N-acetyl-glucosamine transporter | F15B10.1 | 1±0 | 4.8±1 | 2.8±0.7 | 1±0 | 3.6±0.6 | 1.6±0. |
| Actin | *act-1* | 1±0 | 1±0 | 1±0 | 1±0 | 1±0 | 1±0 |

| **Gene function** | ***nhr-8(ok186)*** | ***nhr-8(ok186)*** | ***nhr-8(ok186)*** |
| --- | --- | --- | --- |
|  | **DMSO** | **FLA** | **NF** |
| UGT | 1.4±0.3 | 24.7±13.8 | 12.1±5.5 |
| UGT | 0.9±0.4 | 6.5±5 | 5.3±3 |
| UGT | 0.6±0 | 6.7±0.5 | 6.4±4.9 |
| UGT | 0.9±0.5 | 3.2±1.9 | 2.6±1.3 |
| ADH | 1.1±0.3 | 6.4±3.2 | 2.7±1.9 |
| CYP450 | 0.9±0.3 | 24.6±19 | 56.5±34.3 |
| GST | 1±0.5 | 7.6±3.7 | 3.6±1.7 |
| SMK | 2.4±0.9 | 9.9±5.8 | 11.8±9.4 |
| Reductase | 2.1±1.7 | 1.7±0.6 | 4.8±2.3 |
| NADH: flavin oxido-reductase/ 12-oxophyto-dienoate reductase | 0.4±0.2 | 7.7±6.3 | 1.2±0.8 |
| TAG lipase | 0.6±0.3 | 8.5±4.2 | 4.3±3.1 |
| Lipid phosphate phosphatase | 0.8±0.3 | 2.9±1.1 | 2±1 |
| Cytochrome b5 | 0.9±0.3 | 3±2.3 | 3.7±1.7 |
| UDP-N-acetyl-glucosamine transporter | 0.7±0.2 | 4.7±2.3 | 3.5±1.8 |
| Actin | 1±0 | 1±0 | 1±0 |
