# Supplementary material for: The Mediator Subunit MDT-15 Confers Metabolic Adaptation to Ingested Material
Source: PLoS Genet. 2008 Feb 29;4(2):e1000021. doi: 10.1371/journal.pgen.1000021 (PMC2265483; doi:10.1371/journal.pgen.1000021)
Supplement: Table S9 — Expression of some toxin-induced MDT-15 targets is affected by MDT-15 depletion in adult worms. QPCR quantification of mRNA levels of MDT-15-dependent detoxification genes in conditionally sterile CF512 worms. Values represent fold changes±SEM in mdt-15(RNAi) worms vs. control(RNAi) worms, calculated from the average relative mRNA levels from three independent biological replicates (mRNA levels normalized to act-1). FLA = fluoranthene. Genes whose induction is compromised more than two-fold in mdt-15(RNAi) worms are highlighted bold. (0.08 MB DOC) [file pgen.1000021.s013.doc]

*Supporting Table S9: Expression of some toxin-induced MDT-15 targets is affected by MDT-15 depletion in adult worms.*

QPCR quantification of mRNA levels of MDT-15-dependent detoxification genes in conditionally sterile CF512 worms. Values represent fold changes ± SEM in *mdt-15(RNAi)* worms *vs.* *control(RNAi)* worms, calculated from the average relative mRNA levels from three independent biological replicates (mRNA levels normalized to *act-1*). FLA = fluoranthene. Genes whose induction is compromised more than two-fold in *mdt-15(RNAi)* worms are highlighted **bold**.

| **Function** | **RNAi clone** | ***control*** | ***control*** | ***mdt-15*** | ***mdt-15*** |
| --- | --- | --- | --- | --- | --- |
|  | **Toxin** | **DMSO** | **FLA** | **DMSO** | **FLA** |
| **UGT** | ***ugt-1*** | 1±0 | 7.09±0.65 | 1.43±0.77 | 2.08±0.62 |
| **UGT** | ***ugt-8*** | 1±0 | 18.56±0.98 | 0.66±0.21 | 6.72±1.25 |
| UGT | *ugt-13* | 1±0 | 3.6±0.87 | 0.43±0.21 | 1.52±0.07 |
| UGT | *ugt-25* | 1±0 | 3.7±0.89 | 0.2±0.13 | 1.19±0.09 |
| UGT | *ugt-58* | 1±0 | 2.1±0.19 | 0.74±0.03 | 1.46±0.07 |
| UGT | *ugt-61* | 1±0 | 1.11±0.11 | 0.64±0.11 | 0.61±0.17 |
| UGT | *ugt-63* | 1±0 | 1.72±0.07 | 0.2±0.13 | 0.7±0.08 |
| ADH | *alh-5* | 1±0 | 2.85±1.01 | 0.4±0.11 | 1.45±0.23 |
| CYP450 | C06B3.3 | 1±0 | 6.53±1.6 | 0.1±0.01 | 1.07±0.12 |
| **GST** | ***gst-5*** | 1±0 | 10.85±3.03 | 1.05±0.2 | 4.16±0.14 |
| **Small molecule kinase** | **T16G1.6** | 1±0 | 3.75±1.36 | 0.1±0.05 | 0.23±0.12 |
| Hydrolase | F37H8.3 | 1±0 | 1.4±0.82 | 0.11±0.02 | 0.15±0.02 |
| Reductase | F25D1.5 | 1±0 | 0.75±0.11 | 1.09±0.52 | 1.12±0.42 |
| FAD-domain | F32D8.12 | 1±0 | 0.82±0.23 | 0.35±0.04 | 0.5±0.06 |
| NADH: flavin oxidoreductase/ 12-oxophyto-dienoate reductase | T10B5.8 | 1±0 | 4.54±0.45 | 0.23±0.19 | 1.06±0.45 |
| TAG lipase | F14E5.5 | 1±0 | 2.09±0.49 | 0.28±0.14 | 0.96±0.25 |
| Lipid phosphate phosphatase | T28D9.3 | 1±0 | 1.84±0.16 | 0.56±0.21 | 0.74±0.26 |
| Cytochrome b5 | C31E10.7 | 1±0 | 1.66±0.15 | 0.3±0.02 | 0.48±0.07 |
| CUB-domain | C29F3.7 | 1±0 | 2.67±0.55 | 0.4±0.1 | 0.95±0.18 |
| UDP-N-acetyl-glucosamine transporter | F15B10.1 | 1±0 | 1.91±0.25 | 0.92±0.11 | 1.4±0.07 |
| C-type lectin | Y19D10A.9 | 1±0 | 8.11±0.35 | 0.64±0.08 | 5.33±1.21 |
| Actin | *act-1* | 1±0 | 1±0 | 1±0 | 1±0 |
| Polymerase II | *ama-1* | 1±0 | 1.11±0.08 | 2.96±1.81 | 1.74±0.36 |
| NHR | *nhr-23* | 1±0 | 1.02±0.13 | 1±0.25 | 1.32±0.24 |
