# Supplementary material for: The Mediator Subunit MDT-15 Confers Metabolic Adaptation to Ingested Material
Source: PLoS Genet. 2008 Feb 29;4(2):e1000021. doi: 10.1371/journal.pgen.1000021 (PMC2265483; doi:10.1371/journal.pgen.1000021)
Supplement: Table S10 — Expression of toxin-induced MDT-15 targets is largely unaffected in nhr-49(RNAi) and sbp-1(RNAi) worms. QPCR quantification of mRNA levels of MDT-15-dependent detoxification genes in N2 L4 stage worms fed with nhr-49 or sbp-1 RNAi. Values represent fold changes±SEM in nhr-49(RNAi) or sbp-1(RNAi) worms vs. control(RNAi) worms, calculated from the average relative mRNA levels from three independent biological replicates (mRNA levels normalized to act-1). FLA = fluoranthene, NF = β-napthtoflavone. (0.11 MB DOC) [file pgen.1000021.s014.doc]

*Supporting Table S10:* *Expression of toxin-induced MDT-15 targets is largely unaffected in* nhr-49(RNAi) *and* sbp-1(RNAi) *worms.*

QPCR quantification of mRNA levels of MDT-15-dependent detoxification genes in N2 L4 stage worms fed with *nhr-49* or *sbp-1* RNAi. Values represent fold changes ± SEM in *nhr-49(RNAi)* or *sbp-1(RNAi)* worms *vs.* *control(RNAi)* worms, calculated from the average relative mRNA levels from three independent biological replicates (mRNA levels normalized to *act-1*). FLA = fluoranthene, NF = β-napthtoflavone.

| **Function** | **RNAi clone** | ***control*** | ***nhr-49*** | ***sbp-1*** | ***control*** | ***nhr-49*** | ***sbp-1*** |
| --- | --- | --- | --- | --- | --- | --- | --- |
|  | **Toxin** | **DMSO** | **DMSO** | **DMSO** | **FLA** | **FLA** | **FLA** |
| SMK/DUF227 | T16G1.6 | 1±0 | 1.95±0.5 | 1.4±0.49 | 1.73±0.34 | 2.63±0.52 | 1.67±0.27 |
| Hydrolase | F37H8.3 | 1±0 | 1.12±0.25 | 1.1±0.19 | 3.97±0.35 | 4.15±0.84 | 3.77±0.77 |
| ADH | *alh-5* | 1±0 | 1.1±0.67 | 1.13±0.66 | 7.53±3.09 | 5.29±2.26 | 4.44±1.89 |
| UGT | *ugt-1* | 1±0 | 1.44±0.84 | 1.03±0.42 | 2.01±0.62 | 1.53±0.55 | 1.1±0.35 |
| UGT | *ugt-58* | 1±0 | 1.17±0.06 | 1.14±0.33 | 2.81±0.25 | 3.71±0.5 | 3.02±0.2 |
| UGT | *ugt-25* | 1±0 | 1.41±0.18 | 1.79±0.45 | 5.41±1.67 | 9.05±3.5 | 7.41±2.38 |
| UGT | *ugt-13* | 1±0 | 1.17±0.11 | 1.06±0.21 | 9.41±1.52 | 7.86±1.93 | 8.41±1.8 |
| GST | *gst-5* | 1±0 | 2.38±1.26 | 1.78±0.61 | 7.26±1.84 | 7.66±1.38 | 8.1±0.43 |
| UGT | *ugt-8* | 1±0 | 1.96±0.8 | 1.53±0.34 | 22.09±2.28 | 22.32±1.55 | 17.78±2.89 |
| Lipid-Phosphate-phosphatase | T28D9.3 | 1±0 | 1.14±0.4 | 1.18±0.27 | 3.35±0.4 | 3.19±0.35 | 2.81±0.17 |
| CYP450 | *cyp-35C1* | 1±0 | 2.46±0.72 | 1.81±0.58 | 24.84±7.32 | 35.22±15.53 | 20.66±7.26 |
| TAG Lipase | F14E5.5 | 1±0 | 1.35±0.27 | 1.11±0.31 | 5.08±0.59 | 6.07±1.16 | 5.12±0.71 |
| CUB-domain | C29F3.7 | 1±0 | 1.02±0.11 | 1.47±0.14 | 4.7±0.47 | 7.15±1.87 | 5.09±0.32 |
| UDP-GlcNac transporter | F15B10.1 | 1±0 | 1.31±0.19 | 1.23±0.26 | 4.81±0.7 | 4.46±0.36 | 3.96±0.89 |
| C-type lectin | Y19D10A.9 | 1±0 | 2.27±1.056 | 1.93±0.47 | 14.93±4.14 | 15.98±1.38 | 14.39±5.65 |
| Oxidoreductase | T10B5.8 | 1±0 | 2.97±0.38 | 1.24±0.36 | 16.89±1.21 | 18.12±4.32 | 12.61±2.77 |
| Reductase | F25D1.5 | 1±0 | 1.46±0.34 | 1.31±0.22 | 2.35±1.01 | 1.86±0.35 | 1.65±0.21 |
| Cytochrome b5 | C31E10.7 | 1±0 | 1.06±0.24 | 0.91±0.38 | 2.63±0.55 | 3.95±0.69 | 2.15±0.29 |
| FAD-domain | F32D8.12 | 1±0 | 1.16±0.29 | 0.89±0.36 | 2.77±1.05 | 2.28±0.75 | 1.09±0.24 |
| UGT | *ugt-63* | 1±0 | 2.29±0.7 | 0.93±0.07 | 5.59±1.23 | 7.28±2.75 | 4.54±0.8 |
| Actin | *act-1* | 1±0 | 1±0 | 1±0 | 1±0 | 1±0 | 1±0 |
|  | *fat-5* | 1+0 | 0.19±0.15 | 0.16±0.14 | 0.11±0.06 | 0.13±0.08 | 0.05±0.02 |
|  | *fat-6* | 1+0 | 0.51±0.02 | 0.2±0.1 | 0.67±0.34 | 0.45±0.07 | 0.14±0.04 |
|  | *fat-7* | 1+0 | 0.01±0.01 | 0.17±0.11 | 0.04±0.03 | 0.04±0.04 | 0.02±0.01 |

| **Function** | ***control*** | ***nhr-49*** | ***sbp-1*** |
| --- | --- | --- | --- |
|  | **NF** | **NF** | **NF** |
| SMK/DUF227 | 2±0.4 | 2.87±0.19 | 2.34±0.4 |
| Hydrolase | 0.88±0.12 | 1.15±0.2 | 0.81±0.08 |
| ADH | 2.58±0.87 | 3.34±1.78 | 2.12±0.81 |
| UGT | 1.87±0.7 | 1.97±0.78 | 1.55±0.36 |
| UGT | 1.41±0.06 | 2±0.12 | 1.46±0.15 |
| UGT | 2.82±0.23 | 4.14±0.63 | 2.88±0.27 |
| UGT | 5.45±1.15 | 4.73±1.15 | 4.6±0.14 |
| GST | 1.66±0.25 | 3.12±1.42 | 3.44±1.3 |
| UGT | 5.8±0.46 | 7.15±1.14 | 6.03±1.4 |
| Lipid-Phosphate-phosphatase | 1.56±0.13 | 1.54±0.17 | 1.45±0.26 |
| CYP450 | 30.14±8.57 | 45.54±16.93 | 36.75±9.33 |
| TAG Lipase | 2.23±0.37 | 3.12±0.14 | 2.16±0.51 |
| CUB-domain | 1.74±0.22 | 2.61±0.34 | 2.61±0.15 |
| UDP-GlcNac transporter | 2.09±0.11 | 2.25±0.24 | 2.37±0.2 |
| C-type lectin | 2.44±0.41 | 5.66±1.95 | 5.72±1.47 |
| Oxidoreductase | 1.37±0.18 | 1.92±0.24 | 3.53±2.04 |
| Reductase | 1.34±0.27 | 1.49±0.33 | 1.73±0.48 |
| Cytochrome b5 | 1.66±0.24 | 2.34±0.12 | 1.6±0.61 |
| FAD-domain | 2.09±0.48 | 1.6±0.54 | 1.08±0.17 |
| UGT | 2.55±0.49 | 3.72±1.01 | 2.54±0.22 |
| Actin | 1±0 | 1±0 | 1±0 |
|  | 0.54±0.21 | 0.46±0.28 | 0.13±0.03 |
|  | 1.3±0.31 | 0.73±0.15 | 0.27±0.03 |
|  | 0.65±0.16 | 0.13±0.06 | 0.04±0.01 |
