# Supplementary material for: The Mediator Subunit MDT-15 Confers Metabolic Adaptation to Ingested Material
Source: PLoS Genet. 2008 Feb 29;4(2):e1000021. doi: 10.1371/journal.pgen.1000021 (PMC2265483; doi:10.1371/journal.pgen.1000021)
Supplement: Table S11 — A statistically significant overlap exists between MDT-15 dependent genes and Cd2+-dependent genes. To determine in unbiased fashion whether MDT-15 is involved in regulation of heavy metal detoxification, we determined the overlap between the genes that are deregulated following MDT-15 depletion and genes responsive to Cd2+ (taken from Cui et al. [45]). For further details, see Materials and Methods. (0.04 MB DOC) [file pgen.1000021.s015.doc]

*Supporting Table S11. A statistically significant overlap exists between MDT-15 dependent genes and Cd^2+^-dependent genes.*

To determine in unbiased fashion whether MDT-15 is involved in regulation of heavy metal detoxification, we determined the overlap between the genes that are deregulated following MDT-15 depletion and genes responsive to Cd^2+^ (taken from Cui et al. [45]). For further details, see Materials and Methods.

|  | **Cd^2+^-induced genes (233 total)** | **Cd^2+^-repressed genes (49 total)** |
| --- | --- | --- |
| **MDT-15 activated genes (187 total)** | Expected overlap: 4 Actual overlap: 19 (*P*-value < 1x10^-5^) | Expected overlap: 1 Actual overlap: 15 (*P*-value < 1x10^-5^) |
| **MDT-15 repressed genes (120 total)** | Expected overlap: 7 Actual overlap: 2.6 (*P*-value = 0.03) | Expected overlap: 0.5 Actual overlap: 2 (*P*-value = 0.2) |
